# Predicting Ti‐49 NMR Chemical Shift With New NMR‐DKH Basis Set

**DOI:** 10.1002/jcc.70258

**Published:** 2025-11-02

**Authors:** Matheus Gunar Ramalho Gomes, Catherine Rodrigues Siqueira de Souza, Diego Fernando da Silva Paschoal, Wagner Batista De Almeida

**Affiliations:** ^1^ LQC‐MM—Laboratório de Química Computacional e Modelagem Molecular Instituto de Química, Universidade Federal Fluminense Niterói Brazil; ^2^ NQTCM: Núcleo de Química Teórica e Computacional de Macaé, Polo Ajuda, Instituto Multidisciplinar de Química, Centro Multidisciplinar UFRJ‐Macaé Universidade Federal do Rio de Janeiro Macaé Brazil

**Keywords:** basis set, DFT, nuclear magnetic resonance, Ti(IV) complexes, Titanium‐49

## Abstract

In the present study, a computational protocol for predicting the Ti‐49 NMR chemical shift (δ^49^Ti) was constructed with our NMR‐DKH basis sets for all atoms, including titanium (Ti), which was developed in this work. Thus, computational protocols were proposed considering 55 different DFT functionals using nonrelativistic Hamiltonian. Besides, four‐components (4c) calculations employing the relativistic modified Dirac–Kohn–Sham Hamiltonian at GIAO‐4c‐BLYP/dyall.VDZ level was also considered. In the best protocol, the structures of the 41 Ti(IV) complexes studied, which cover a wide range of δ^49^Ti ranging from −1389 to +1325 ppm, were optimized at the BLYP/def2‐SVP/IEF‐PCM (UFF) level and the δ^49^Ti was calculated at the GIAO‐OLYP/NMR‐DKH/IEF‐PCM (UFF) level, both employing a nonrelativistic Hamiltonian. In this protocol, a mean absolute deviation (MAD) of only 48 ppm and a coefficient of determination (*R* ^2^) of 0.9888 were found, which represents an excellent agreement, and with lower computational cost, with the MAD of 62 ppm and *R* ^2^ of 0.9860 obtained with the relativistic full 4c Hamiltonian at GIAO‐4c‐BLYP/dyall.VDZ, indicating that the proposed protocol with the NMR‐DKH basis set is an excellent alternative for the study of Ti‐49 NMR. Additionally, a predictive model based on linear regression (δTicalc49=−1.0027×σcalc−1000.0) was developed, adjusted from 41 complexes and validated in an external set of nine Ti(IV) complexes, presenting a MAD of 48 ppm and confirming the robustness and extrapolation capacity of the proposed protocol.

## Graphical abstract

A new NMR‐DKH basis set was proposed for Ti atom and the δ^49^Ti was studied in a set of 41 + 9 (calibration‐blue/validation‐red) Ti(IV) complexes, resulting in a mean absolute deviation of only 50 ppm with the GIAO‐OLYP/NMR‐DKH/IEF‐PCM(UFF)//BLYP/def2‐SVP/IEF‐PCM(UFF) protocol.

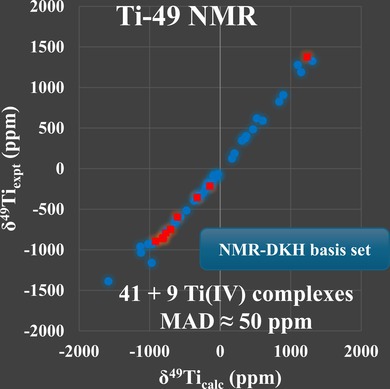

## 1 Introduction

Titanium (Ti), a transition metal with electronic configuration [Ar]4s^2^3d^2^, has been widely studied in several areas and is widely found in the Earth's crust in the oxidation state +IV [1]. Its excellent mechanical properties, such as low density, and corrosion resistance have made it very interesting for industrial and technological processes. In the chemical industry, Ti‐based compounds are used as catalysts in a diverse range of chemical reactions [2, 3, 4, 5, 6, 7]. In recent years, Ti complexes have also been widely studied as effective candidates for anticancer agents. Its biological application is advocated by its low toxicity and biocompatibility, enabling the development of potential drugs [1, 8, 9, 10, 11].

In the biological context, Ti complexes have been extensively investigated, especially after the titanocene dichloride complex, [Ti(Cp_2_)Cl_2_] (Cp = η^5^‐C_5_H_5_), became the first metallocene and the first non‐platinum‐based metal complex to enter clinical trials as an anticancer compound [12]. This complex had its biological activity reported in 1970 by Kopf and Kopf–Maier, reaching clinical trials in 1990. Despite being successful in phase I, this complex did not show activity in patients tested in phase II and was discontinued [13]. However, the success obtained with Ti‐based metal complexes demonstrated that it is possible to develop new non‐platinum‐based drugs that have promising biological activity [11, 14].

In order to overcome the disadvantages and limitations of Ti complexes, several studies have been carried out seeking structural changes that enable greater solubility, stability and molecular specificity, providing improved biological activity [15, 16]. To this end, understanding the electronic, magnetic and structural environment of such molecules is essential.

Nuclear magnetic resonance (NMR) spectroscopy is one of the main techniques used in structural characterization, being essential to providing structural and electronic information about metal complexes [17, 18, 19]. Titanium has two NMR‐active isotopes: ^47^Ti and ^49^Ti. The small difference between their gyromagnetic ratios results in very close resonant frequencies, separated by 271 ppm, with the ^49^Ti resonance always being higher. Both isotopes are quadrupolar; that is, they have a nuclear spin quantum number *I* ≥ 1, being 5/2 for ^47^Ti and 7/2 for ^49^Ti. The ^49^Ti nucleus is the most used in experimental measurements due to its greater relative sensitivity. Experimental measurements use the pure [TiCl_4_] compound as a reference. The NMR chemical shift of Ti‐49 (δ^49^Ti) shows a variation from +1375 ppm for [Ti(CH_2_CMe_2_Ph)_4_] to −1389 ppm for [Ti(CO)_6_]^2−^ [20, 21].

Given the importance of Ti complexes and Ti‐49 NMR, computational studies evaluating δ^49^Ti can be found in the literature. In 2004, Bühl and Mauschick [22] published a study involving the δ^49^Ti of 10 Ti(IV) complexes with structures optimized at the BP86/AE1 level of theory, and with nuclear shielding tensors obtained with the gauge‐including atomic orbital (GIAO) method using the BPW91 and B3LYP density functionals, as well as the Hartree–Fock (HF) method. Considering the 10 Ti(IV) complexes studied, the authors found a mean absolute deviation (MAD) of 127 ppm (GGA BPW91) and 110 ppm (Hybrid B3LYP). Koch and Bruhn [23] address the evaluation of the δ^49^Ti from the extension of the levels of theory tested. Excellent results are found when applying the B3LYP/6‐31G(d)//B3LYP/6‐31G(d) protocol, with an average error of 67 ppm, considering a set of 14 Ti(IV) complexes. The MP2/6‐31G(d)//MP2/6‐31G(d) level of theory also stands out for the good results found. The evaluation of the influence of the solvent effect was done using the implicit solvation model PCM. Furthermore, the authors attribute the good results with a small basis set to error cancellation. Thus, the authors conclude that further studies on the subject are necessary. Recently, Schattenberg et al. [24] evaluated modern DFT functionals for predicting NMR chemical shifts of 3d transition metal nuclei. The authors studied 12 Ti(IV) complexes with optimized geometries at the BP86‐D3BJ/def2‐TZVPD/def2‐TZVP and TPSSh‐D3BJ/def2‐TZVPD/def2‐TZVP levels and evaluated δ^49^Ti for these structures considering different DFT functionals with the pcSseg‐3 basis sets, which for titanium, for example, presents 117 CGTO‐[7s10p6d3f2g1h], the role of the solvent and relativistic effects. The authors obtained a good result at the GIAO‐cM06L/pcSseg‐3 level, but with a high computational cost and for a small set of complexes only.

In previous papers, Paschoal et al. developed the NMR‐DKH basis sets for the atoms of H–He, Li–Ne, Na–Ar, K–Ca, Ga–Kr, Rb–Sr, In–Xe, and Pt [25], Tc [26], and Co [27] and described in excellent agreement with the experimental data and with low computational cost the δ^195^Pt [25, 28], δ^99^Tc [26], δ^59^Co [27], ^1^J(^195^Pt‐^15^N) [29], ^1^J(^195^Pt‐^31^P) [28], and ^1^J(^129^Xe‐^19^F) [30]. Thus, in the present work the NMR‐DKH basis set was developed for the Ti atom and a robust computational protocol for predicting the Ti‐49 NMR chemical shift was proposed.

## 2 Theoretical Methodology

### 2.1 Development of NMR‐DKH Basis Set

The NMR‐DKH basis set, a segmented all‐electron Gaussian basis set relativistically contracted using the second‐order Douglas–Kroll–Hess (DKH2) approach, for the Ti atom was constructed using the same methodology used previously by Paschoal et al. for H–He, Li–Ne, Na–Ar, K–Ca, Ga–Kr, Rb–Sr, In–Xe and Pt [25], Tc [26], and Co [27] atoms.

Initially, the maximum exponents per angular momentum (*α*_l_, l = *s*, *p*, *d*, *f*) were obtained with the following equation:

(1) $$\alpha_{l}=k_{l}\frac{2f_{l}^{2}}{\pi {\left\langle r_{l}\right\rangle }^{2}}$$

where *k*_1_ is a scaling factor, used to generate exponents for each angular momentum aiming to describe properly the core electrons and it assumes the values 1, 4/3, and 8/5 values for the angular momenta *s*, *p*, and *d*, respectively, while the *f*_1_ assumes the values 33 (*s*), 100 (*p*) and 1000 (*d*). The *r*_1_ is the innermost radial value, that it was obtained with multiconfigurational Dirac–Fock (MCDF) numerical calculations for the Ti atom in its ground state (F23). The MCDF calculations were realized in GRASP90 software [31], resulting. The following α_l_ values were obtained: *α*_*s*_ = 131685.903249881; *α*_*p*_ = 1375.44747973736; *α*_*d*_ = 24.0073133461147.

Subsequently, descending series of primitive functions were generated according to following equation:

(2) $$\zeta =\alpha_{l}\chi^{-i}\;\left(\text{where}\;i\;\text{is}\;a\;\text{positive integer}\right)$$

where *i* is a positive integer, *ζ* is the exponent for Gaussian basis functions and *χ* is a parameter that describes the spacing and number of primitives. The considered values of *χ* were 2.50 (*s*), 2.75 (*p*), and 3.00 (*d*). A set of 18*s*, 11*p*, and 6*d* primitive functions were obtained and contracted as a triple‐zeta basis set in which only the first function for each angular momentum was contracted. The contraction coefficients were generated using an unrestricted Hartree–Fock (UHF) calculation with inclusion of scalar relativistic corrections through the second‐order Douglas–Kroll–Hess (DKH2) approximation [32, 33, 34, 35, 36, 37, 38]. These calculations were performed using GAUSSIAN 16 Rev. C.01 program [39].

Finally, the obtained triple‐zeta basis sets were augmented with three sets of *f*‐polarization functions, which had their exponents adjusted to minimize energy at the UHF‐DKH2 level considering the effects of an electric field (*z* = 0.01 a.u.), with the two *f*‐polarizations functions with highest exponents being contracted [40]. These calculations were also performed in the GAUSSIAN 16 Rev. C.01 program [39].

Then, the obtained NMR‐DKH basis set, which presents a triple‐zeta‐doubly polarized character (TZ2P), has the following contraction scheme: (18s11p6d3f) → [12s6p3d2f], with a set of 102 GTO (Gaussian‐type orbitals) and 59 CGTO (Contracted Gaussian‐type orbitals). The NMR‐DKH basis set for Ti atom can be obtained in Supporting Information or downloaded in Basis Set Exchange Portal (https://www.basissetexchange.org/) [41].

### 2.2 Assessment of DFT Functionals in Predicting the Ti‐49 NMR Chemical Shift

After the development of NMR‐DKH basis set, computational protocols (NMR/Geometry) employing nonrelativistic Hamiltonians for predicting the Ti‐49 chemical shifts were proposed.

**FIGURE 1:**
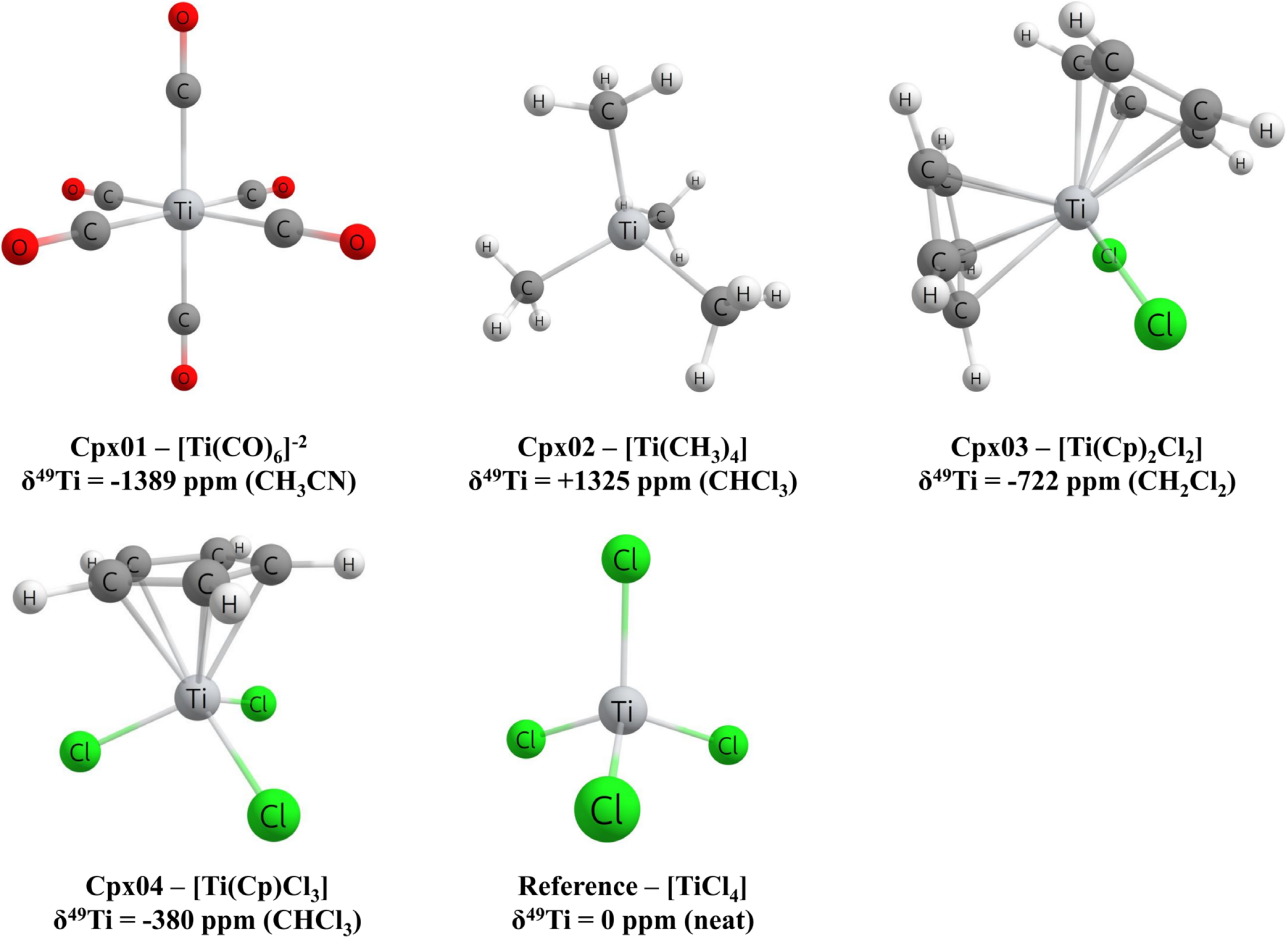
Ti(IV) complexes selected for the construction of computational protocol for predicting the δ^49^Ti.

Initially, a set of four Ti(IV) complexes, [Ti(CO)_6_]^−2^ in CH_3_CN [42], [Ti(CH_3_)_4_] in CHCl_3_ [43], [Ti(Cp)_2_Cl_2_] in CH_2_Cl_2_ [44], and [Ti(Cp)Cl_3_] in CHCl_3_ [45], with distinct experimental Ti‐49 NMR chemical shift available in the literature, and the reference [TiCl_4_] neat that is used as an internal reference in the experimental measurement, were selected (Figure 1). Furthermore, for the [Ti(CO)_6_]^−2^ [46], [Ti(Cp)_2_Cl_2_] [47], [Ti(Cp)Cl_3_] [48], and reference [TiCl_4_] [49] experimental structure data obtained by X‐ray diffraction are also found. For the complex [Ti(CH_3_)_4_], the bond length of the Ti–Me obtained from the X‐ray structure of the complex [Ti(MePh)_4_] [50] was used as reference.

The structures of the selected complexes were optimized and characterized as stationary points on the potential energy surface (PES) through harmonic frequency calculations (all frequencies are real) at the BLYP/def2‐SVP/IEF‐PCM(UFF) level. This protocol is the same used in a previous paper [27] to describe the structure of cobalt(III) complexes in predicting Co‐59 NMR chemical shift. The solvent effects, in both structure and NMR calculations, were included implicitly using the Integral Equation Formalism for Polarizable Continuum Model (IEF‐PCM) with Radii of Universal Force Field (UFF) [51], considering the same solvent used in the experimental data.

Subsequently, the Ti‐49 NMR shielding constant tensor (σ^49^Ti) was calculated using the Gauge Independent Atomic Orbital (GIAO) approach [52, 53, 54, 55, 56] at the DFT‐Functional/NMR‐DKH/IEF‐PCM(UFF) level, considering a set of 55 DFT functionals (Table 1). Then, the Ti‐49 NMR chemical shift (δ^49^Ti) was calculated with the following equation:

(3) δTi49=σref−σcalc

**TABLE 1:** DFT functionals used in the construction of the computational protocol for predicting the δ^49^Ti.

LDA	GGA	meta‐GGA	Hybrid	Hybrid meta‐GGA	LRC	Dispersion
SVWN [57, 58, 59]	mPWLYP [60, 61, 62]	VSXC [63]	mPW1LYP [60, 61, 62]	B1B95 [64]	ωB97 [65]	B97‐D3BJ [66]
	mPWPBE [60, 67, 68]	BB95 [64, 69]	mPW3PBE [60, 70]	TPSSh [71, 72, 73]	ωB97x [65]	ωB97xD [74]
	mPWPW91 [60, 70, 75, 76]	TPSS [71]	mPW1PW91 [60, 77]	BMK [78]	LC‐BLYP [61, 62, 69, 79]	
	OLYP [61, 62, 80, 81]	revTPSS [82, 83]	O3LYP [84]	PW6B95 [85]	LC‐wPBE [86, 87, 88]	
	BLYP [61, 62, 69]	M06L [89]	B3LYP [62, 90, 91]	M06HF [92, 93]	CAM‐B3LYP [94]	
	XLYP [57, 58, 61, 62]	M11L [95]	X3LYP [96]	M06 [97]	M11 [98]	
	PW91 [68, 75, 76]	MN12L [99]	B3PW91 [75, 90]	M062X [97]	MN12SX [100]	
	BPW91 [69, 70, 75, 76]	MN15L [101]	B3P86 [90, 102]	MN15 [103]		
	BP86 [69, 102]	τHCTH [104]	PBE0 [105, 106]	τHCTHhyb [104]		
	PBE [67, 68]		PBEh1PBE [107]			
	PBEhPBE [67, 70, 107]		BHandH [108]			
	OPBE [67, 70, 80, 81]		BHandHLYP [108]			
	SOGGA11 [98]		SOGGA11X [109]			
			B97‐2 [110]			

With *σ*_ref_ representing the Ti‐49 shielding constant tensor for the internal reference, [TiCl_4_] neat, and *σ*_calc_ represents the Ti‐49 shielding constant tensor for each complex of interest.

It is important to highlight that the relativistic DKH Hamiltonian was not used in the NMR calculations, being only the basis sets relativistically contracted within the framework of the DKH2 approximation aiming at a better description of the shielding constants, in the same way that was done for the Pt‐195 [25, 28], Tc‐99 [26], and Co‐59 [27] nuclei.

The quality of each computational protocol was evaluated according to the mean absolute deviation (MAD) in relation to the experimental values [27].

(4) $$\mathrm{MAD}=\frac{1}{n}\sum_{i=1}^{n}\left|\mathrm{AD}\right|∴\mathrm{AD}=\left|s_{i}^{\exp}-s_{i,j}^{\text{calc}}\right|$$

where AD corresponds to the absolute deviation in relation to the experimental value, *i* is the property evaluated (Ti‐49 NMR chemical shift), and *j* is the considered computational protocol.

The calculations were carried out with the GAUSSIAN 16 Rev. C.01 program [39].

### 2.3 Validation of the Best Computational Protocols

The best computational protocols (labeled as Model 1) were applied for predicting the Ti‐49 NMR chemical shift (Equation 3) of other 37 Ti(IV) complexes, totally, a set of 41 Ti(IV) complexes was studied, with the δ^49^Ti ranging from −1389 to +1325 ppm. Finally, the best model was validated with a linear regression model and with respect to its ability to describe the experimental behavior of δ^49^Ti.

### 2.4 The Role of the Relativistic Effects

The Ti‐49 shielding constants for the 41 Ti(IV) complexes studied were also obtained from relativistic full four‐component (4c) calculations using the modified Dirac–Kohn–Sham (mDKS) Hamiltonian with the GGA BLYP functional and the dyall.VDZ basis sets, protocol named as GIAO‐4c‐BLYP/dyall.VDZ. Furthermore, one‐component (nonrelativistic) calculations at the GIAO‐BLYP/dyall.VDZ level were also performed to evaluate the role of relativistic effects in describing the Ti‐49 NMR chemical shift. For both protocols the δ^49^Ti was calculated according to Equation (3).

The calculations with the 4c‐BLYP/dyall.VDZ and BLYP/dyall.VDZ protocols were performed in ReSpect 5.3.0 program [111].

### 2.5 Predictive Modeling and External Validation

In order to evaluate the robustness of the best computational protocol employing a nonrelativistic Hamiltonian, a statistical linear regression model was proposed for calculating the δ^49^Ti. In this model, the calculated nuclear shielding constant tensor was considered the independent variable (σ^49^Ti_calc_), while the experimental chemical shift was used as the dependent variable (δ^49^Ti_expt_), defining the relationship σ^49^Ti_calc_ × δ^49^Ti_expt_. Thus, δ^49^Ti_calc_ was calculated according to Equation (5), in which parameters “*a*” and “*b*” were adjusted considering the 41 complexes previously studied and validating for another nine complexes.

(5) δTicalc49=a×σcalc+b

It is interesting to note that Equation (5) reduces to Equation (3) if *a* = −1 and *b* = *σ*_ref_, indicating that the predictive model is physically consistent. Furthermore, the model has the advantage of recovering, at least partially, aspects that were not considered in the quantum mechanics (QM) protocol, such as relativistic effects in Hamiltonian.

The *xyz* coordinates of the optimized structures at the BLYP/def2‐SVP/IEF‐PCM(UFF) level of all 50 Ti(IV) complexes and the reference compound in Ti‐49 NMR can be found in the Supporting Information, as well as a figure with the respective 3D structures.

## 3 Results and Discussion

### 3.1 The Role of the DFT Functional in Predicting the Ti‐49 NMR Chemical Shift

#### 3.1.1 Structure of Ti(IV) Complexes

Geometry optimization is a fundamental step in computational studies using Quantum Chemistry methods, especially when the interest is in the NMR properties. In a previous work [27] involving the computational prediction of δ^59^Co in Co(III) complexes, also a 3d transition metal like Ti, it was shown that the BLYP/def2‐SVP/IEF‐PCM(UFF) protocol presents a good description of the geometry of the complexes. In addition, other studies have also shown that the def2‐SVP basis sets are a good alternative for the description of the geometry of transition metal complexes [25, 26, 28, 29].

Thus, the geometry of the complexes [Ti(CO)_6_]^−2^, [Ti(CH_3_)_4_], [Ti(Cp)_2_Cl_2_], [Ti(Cp)Cl_3_], and Ref—[TiCl_4_], selected for the construction of the computational protocol for predicting the δ^49^Ti, was optimized at the BLYP/def2‐SVP/IEF‐PCM(UFF) level. In order to validate the protocol for the description of the geometry of Ti(IV) complexes, a comparison with the experimental data obtained by X‐ray diffraction was performed.

From the optimized structures of the complexes, an analysis of the Ti−Ligands bond lengths in the complexes [Ti(CO)_6_]^−2^, [Ti(Cp)_2_Cl_2_], [Ti(Cp)Cl_3_], and Ref—[TiCl_4_] was performed. For the complex [Ti(CH_3_)_4_], the Ti–C bond lengths in the complex [Ti(MePh)_4_] was used as reference. According to the calculated values (Table 2), for [Ti(CO)_6_]^−2^ a relative deviation (RD) of 1.5% in relation to the experimental value was found for the Ti–C bond lengths, while for the complex [Ti(CH_3_)_4_] a RD of 1.9% was found for the Ti–C. For the two complexes with the cyclopentadienyl (Cp) ligand with η^5^ binding mode with the Ti metal center, the average of the Ti–C distances were considered. Thus, for [Ti(Cp)_2_Cl_2_] an RD of 3.6% was found for the Ti–C (Cp) distances and 0.4% for the Ti–Cl distances, while for the [Ti(Cp)Cl_3_] complex an RD of 3.9% and 1.2% was found for the Ti–C (Cp) and Ti‐Cl distances, respectively. Finally, for [TiCl_4_] an RD of 1.4% was found for the Ti–Cl bond length.

**TABLE 2:** Calculated bond lengths at BLYP/def2‐SVP/IEF‐PCM(UFF) level for the Ti(IV) complexes used in the construction of the computational protocol for predicting the δ^49^Ti.

Ti(IV) complexes	Bond lengths (Å)	Calc.	Expt.	RD
Cpx01—[Ti(CO)_6_]^−2^	Ti‐C	2.07	2.04 [46]	1.5%
Cpx02—[Ti(CH_3_)_4_]	Ti‐C	2.10	2.14 [50]	1.9%
Cpx03—[Ti(Cp)_2_Cl_2_]	Ti‐Cl	2.37	2.36 [47]	0.4%
	Ti‐C (Cp)	2.45	2.36 [47]	3.6%
Cpx04—[Ti(Cp)Cl_3_]	Ti‐Cl	2.25	2.22 [48]	1.2%
	Ti‐C (Cp)	2.41	2.32 [48]	3.9%
Ref—[TiCl_4_]	Ti‐Cl	2.20	2.17 [49]	1.4%
Mean relative deviation (MRD)				2.0%

*Note:* References to experimental values obtained by X‐ray diffraction are given in square brackets.

Therefore, the calculated values for the Ti–Ligands bond lengths show that the BLYP/def2‐SVP/IEF‐PCM(UFF) protocol is a good alternative for the description of the structure of Ti(IV) complexes. Overall, considering all bond lengths evaluated, a mean relative deviation (MRD) of only 2.0% (Table 2) was found, with all calculated bonds being overestimated in relation to the X‐ray structures. It should be taken into account that these results are expected since in the experimental values the structures are in the solid state, and the calculated values are in solution.

#### 3.1.2 Protocol for Predicting the δ^49^Ti

Considering that the theoretical structures for the five Ti(IV) complexes (Figure 1) selected for the construction of the computational protocol present a good agreement with the experimental data, an assessment of the role of the DFT functional in the description of δ^49^Ti was performed. Thus, computational protocols employing nonrelativistic Hamiltonian at the GIAO‐DFT‐Functional/NMR‐DKH/IEF‐PCM(UFF) level, considering 55 distinct DFT functionals (Table 1), were proposed.

**FIGURE 2:**
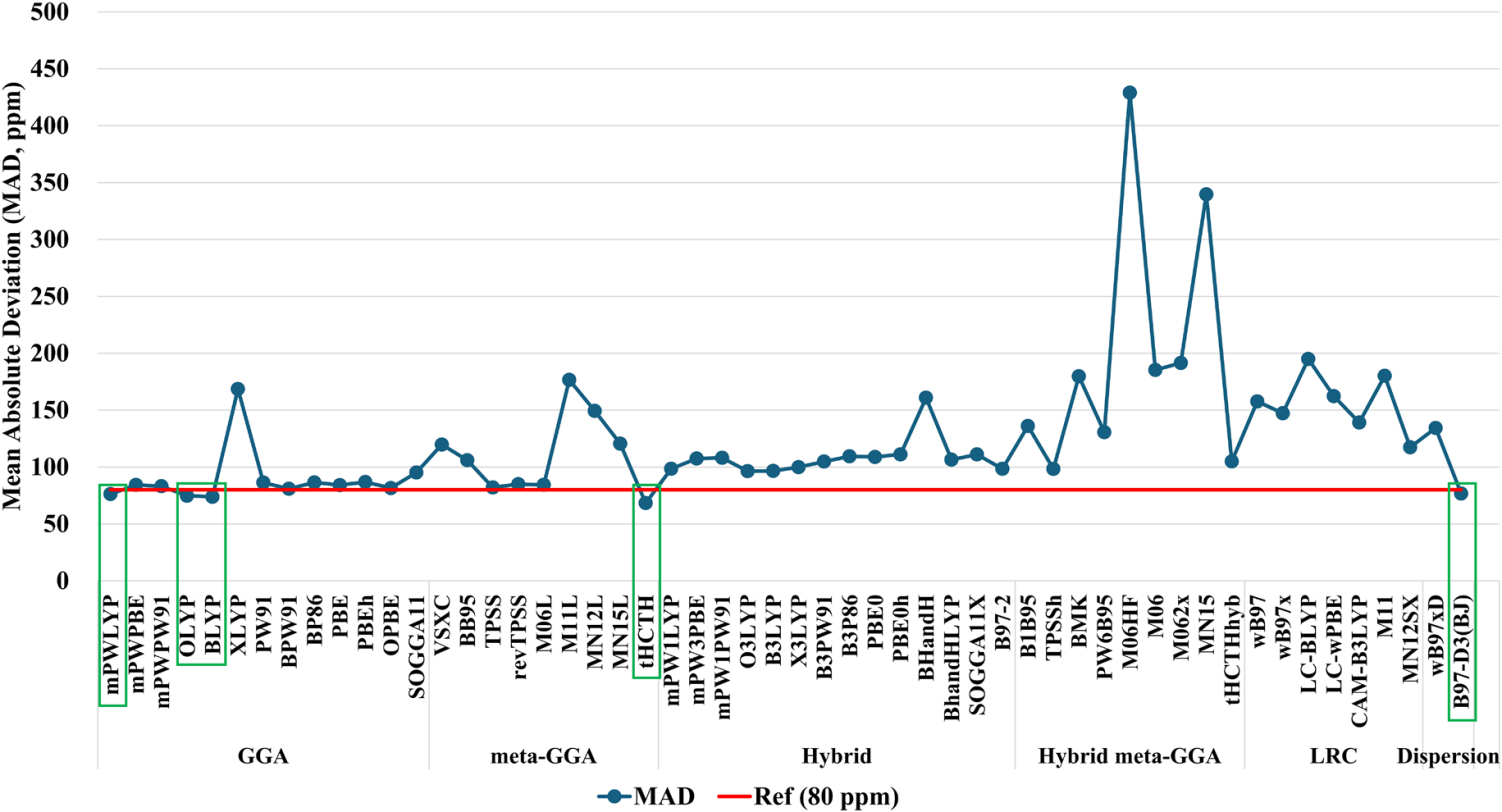
Calculated mean absolute deviation (MAD, ppm)—considering the Ti(IV) complexes [Ti(CO)_6_]^−2^, [Ti(CH_3_)_4_], [Ti(Cp)_2_Cl_2_], and [Ti(Cp)Cl_3_]—for δ^49^Ti at GIAO‐DFT‐Functional/NMR‐DKH/IEF‐PCM(UFF) levels, for the set of 55 DFT functionals.

From the results obtained for the mean absolute deviation (MAD; Equation 4) considering the four selected complexes (Figure 2), it is observed that the δ^49^Ti is very sensitive to the DFT functional employed. The calculated MAD values showed a variation between 68 ppm (meta‐GGA τHCTH) and 429 ppm (Hybrid meta‐GGA M06‐HF). The calculated values for δ^49^Ti are presented in Table S1.

Considering the results obtained for each complex separately, it is observed that the δ^49^Ti for the [Ti(CO)_6_]^−2^, which presented the largest absolute deviations (AD; Equation 4) in relation to the experimental value, did not show a great sensitivity to the DFT functional employed. In general, the calculated AD was ~210 ppm; an exception was observed when the Minnesota meta‐GGA functionals were considered, where AD < 100 ppm was observed. It is interesting to highlight that for this complex, experimental values are found in the literature for the chemical shift of the ^49^Ti nuclei (−1389 ppm) and also for the ^47^Ti nucleus (−1655 ppm) [42]. If the value found for the ^47^Ti nucleus is considered, the AD decreases from ~210 to ~61 ppm.

For the complexes [Ti(CH_3_)_4_], [Ti(Cp)_2_Cl_2_], and [Ti(Cp)Cl_3_], the variations in the calculated values of δ^49^Ti are more pronounced. However, it is observed that the GGA functionals, the meta‐GGA τHCTH, and the GGA including dispersion corrections B97‐D3BJ presented the best description. It is worth noting that the Ti(IV) complexes have Ti with electronic configuration *d* ^0^. The GGA functionals, with the inclusion of the electron density gradient, present a good description of the regions close to the Ti nucleus [112, 113], which is mainly important for the diamagnetic term of the shielding constant tensor [114]. When the complexes with the Cp ligand are considered, it is also important to take into account the need for an adequate description of the regions with π bonds and the greater asymmetry in the electron density. The inclusion of the density kinetic energy term in meta‐GGA functionals, such as τHCTH, allows a better fit of the density near the nucleus. On the other hand, hybrid and LRC functionals may present density distortions [112, 113, 115]. The B97‐D3BJ functional, in addition to the advantages presented above for GGA functionals, with the inclusion of the dispersion term can improve the description of weak interactions such as Cp⋯Cp and Cl⋯H–Cp, which can be observed by the better description of δ^49^Ti in the complexes [Ti(Cp)_2_Cl_2_] and [Ti(Cp)Cl_3_].

Thus, considering what was discussed, the DFT functionals that presented a MAD < 80 ppm, that is, mPWLYP (76 ppm), OLYP (75 ppm), BLYP (74 ppm), τHCTH (68 ppm), and B97‐D3BJ (77 ppm), were selected for the study of δ^49^Ti in another 37 Ti(IV) complexes.

#### 3.1.3 Validation of Computational Protocol for Predicting the δ^49^Ti

Aiming at the validation of the best computational protocols obtained in the previous step, that is, those with MAD < 80 ppm, a set of 37 Ti(IV) complexes, with experimental data of δ^49^Ti available in the literature, were selected, totaling 41 Ti(IV) complexes to be studied. The geometry of all complexes was also optimized at the BLYP/def2‐SVP/IEF‐PCM(UFF) level, with the δ^49^Ti being calculated with the GIAO‐DFT‐Functional/NMR‐DKH/IEF‐PCM(UFF) protocols, with the DFT functionals mPWLYP, OLYP, BLYP, τHCTH, and B97‐D3BJ being evaluated.

**FIGURE 3:**
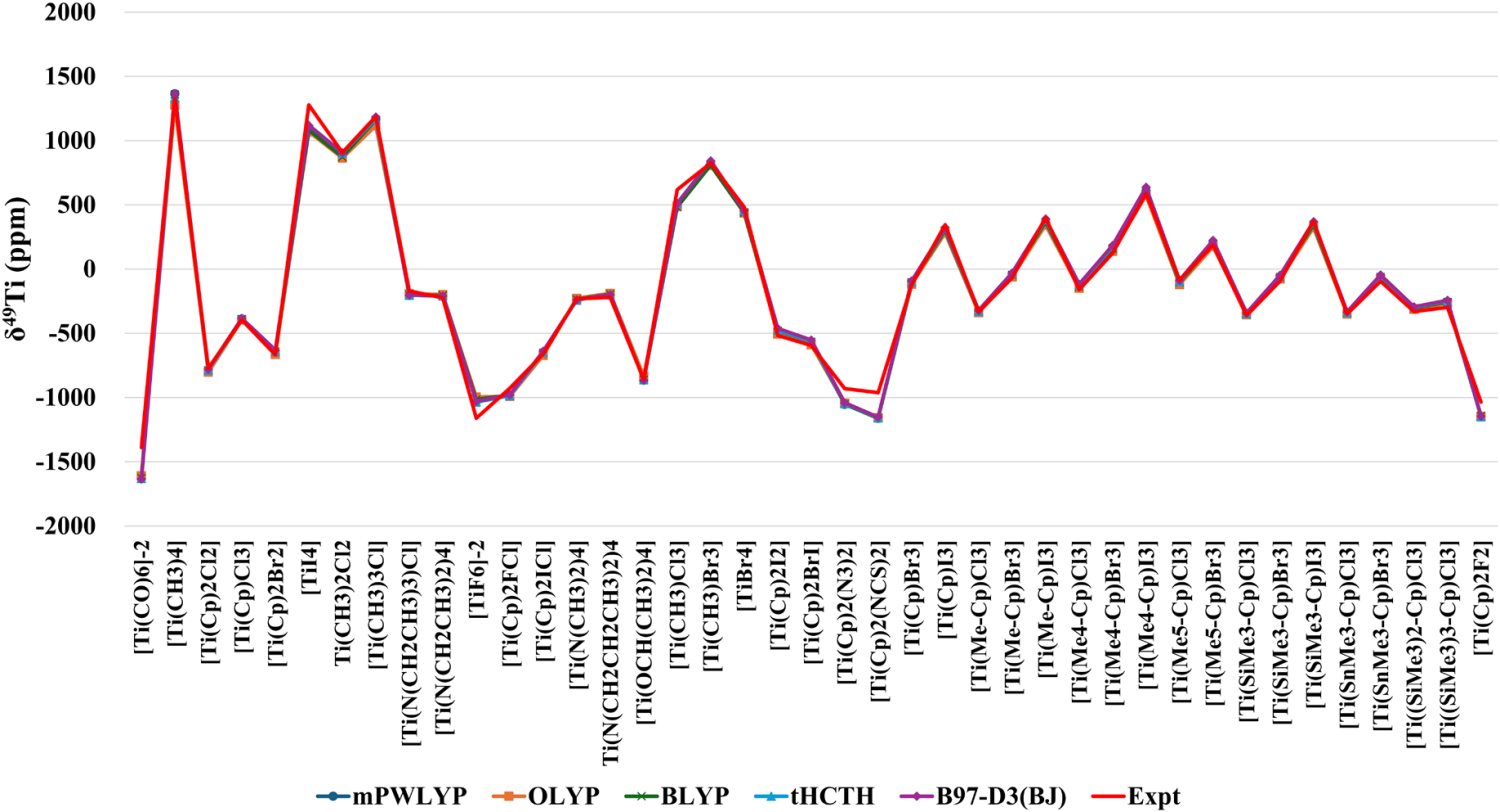
Comparison between experimental and calculated δ^49^Ti at GIAO‐DFT‐Functional/NMR‐DKH/IEF‐PCM(UFF), considering the mPWLYP, OLYP, BLYP, τHCTH, and B97‐D3(BJ) functionals, for a set of 41 Ti(IV) complexes.

From the calculated values for δ^49^Ti of the 41 Ti(IV) complexes studied (Table 3), it can be observed that a good agreement with the experimental data was obtained. The calculated MAD was lower than 50 ppm, being 48 ppm (mPWLYP, OLYP, BLYP, and B97‐D3(BJ)) and 45 ppm (τHCTH). In addition to the MAD, an analysis of the standard deviation (SD) of the calculated absolute deviations can also be considered in order to evaluate the consistency of the protocols, that is, whether the calculated MAD is a consequence of some very high and others very low AD, or whether they are a consequence of more constant AD. The calculated SD values (Table 3) show a good consistency of all protocols, with SD ranging from 53 ppm (τHCTH and B97‐D3BJ) to 58 ppm (OLYP). In Figure 3 it can be observed that the protocols with the five DFT functionals adequately describe the experimental trend of δ^49^Ti for the 41 Ti(IV) complexes studied.

**TABLE 3:** Calculated δ^49^Ti (ppm) at GIAO‐DFT‐Functional/NMR‐DKH/IEF‐PCM(UFF) levels for a set of 41 Ti(IV) complexes.

Ti(IV) complexes	Solvent	mPWLYP	OLYP	BLYP	τHCTH	B97‐D3(BJ)	Expt.
[Ti(CO)_6_]^−2^	CH_3_CN	−1630	−1608	−1626	−1631	−1635	−1389 [42]
[Ti(CH_3_)_4_]	CHCl_3_	1365	1277	1360	1333	1366	1325 [43]
[Ti(Cp)_2_Cl_2_]	CH_2_Cl_2_	−787	−803	−787	−788	−779	−772 [44]
[Ti(Cp)Cl_3_]	CHCl_3_	−388	−396	−389	−389	−384	−396.5 [45]
[Ti(Cp)_2_Br_2_]	CH_3_CN	−642	−664	−643	−642	−631	−668.3 [44]
[TiI_4_]	C_6_H_6_	1082	1069	1081	1121	1117	1278.3 [44]
[Ti(CH_3_)_2_Cl_2_]	CH_2_Cl_2_	877	865	874	904	906	907 [43]
[Ti(CH_3_)_3_Cl]	CH_2_Cl_2_	1167	1117	1163	1168	1184	1188 [44]
[Ti(N(CH_2_CH_3_)_3_)Cl]	CHCl_3_	−204	−192	−204	−203	−202	−171 [116]
[Ti(N(CH_2_CH_3_)_2_)_4_]	CH_2_Cl_2_	−205	−197	−205	−210	−208	−224 [116]
[TiF_6_]^−2^	H_2_O	−1016	−997	−1015	−1036	−1030	−1160.9 [117]
[Ti(Cp)_2_FCl]	CH_2_Cl_2_	−980	−991	−981	−986	−978	−928.3 [44]
[Ti(Cp)_2_ICl]	CH_2_Cl_2_	−650	−671	−651	−649	−639	−661.2 [44]
[Ti(N(CH_3_)_2_)_4_]	CH_2_Cl_2_	−236	−227	−236	−241	−238	−231 [116]
Ti(N(CH_2_CH_2_CH_3_)_2_)_4_	CH_2_Cl_2_	−197	−189	−197	−202	−199	−221 [116]
[Ti(OCH(CH_3_)_2_)_4_]	CH_2_Cl_2_	−865	−835	−863	−862	−865	−859 [116]
[Ti(CH_3_)Cl_3_]	CH_2_Cl_2_	484	491	483	514	509	618 [116]
[Ti(CH_3_)Br_3_]	CH_2_Cl_2_	811	808	809	843	842	825 [116]
[TiBr_4_]	CH_2_Cl_2_	441	437	441	456	455	482.9 [44]
[Ti(Cp)_2_I_2_]	CH_2_Cl_2_	−479	−506	−480	−476	−463	−517.2 [44]
[Ti(Cp)_2_BrI]	CH_2_Cl_2_	−565	−589	−566	−564	−553	−595.1 [44]
[Ti(Cp)_2_(N_3_)_2_]	CHCl_3_	−1052	−1045	−1051	−1044	−1041	−930.9 [44]
[Ti(Cp)_2_(NCS)_2_]	CH_2_Cl_2_	−1162	−1158	−1161	−1157	−1152	−962.3 [44]
[Ti(Cp)Br_3_]	CHCl_3_	−102	−117	−103	−98	−92	−123.8 [45]
[Ti(Cp)I_3_]	CH_2_Cl_2_	303	280	302	316	323	345.2 [45]
[Ti(Me‐Cp)Cl_3_]	CH_2_Cl_2_	−329	−339	−330	−329	−323	−332 [45]
[Ti(Me‐Cp)Br_3_]	CH_2_Cl_2_	−43	−60	−44	−37	−30	−68.8 [45]
[Ti(Me‐Cp)I_3_]	CH_2_Cl_2_	369	343	367	383	391	399.5 [45]
[Ti(Me_4_‐Cp)Cl_3_]	CH_2_Cl_2_	−133	−149	−135	−125	−118	−161.1 [45]
[Ti(Me_4_‐Cp)Br_3_]	CH_2_Cl_2_	163	139	160	175	184	123.5 [45]
[Ti(Me_4_‐Cp)I_3_]	CH_2_Cl_2_	605	574	602	627	637	589.4 [45]
[Ti(Me_5_‐Cp)Cl_3_]	CHCl_3_	−101	−120	−103	−96	−87	−85 [45]
[Ti(Me_5_‐Cp)Br_3_]	CHCl_3_	203	175	199	213	223	186.7 [45]
[Ti(SiMe_3_‐Cp)Cl_3_]	CH_2_Cl_2_	−347	−355	−348	−346	−342	−361.4 [45]
[Ti(SiMe_3_‐Cp)Br_3_]	CH_2_Cl_2_	−61	−77	−62	−54	−48	−93.2 [45]
[Ti(SiMe_3_‐Cp)I_3_]	CH_2_Cl_2_	347	323	345	362	369	373.5 [45]
[Ti(SnMe_3_‐Cp)Cl_3_]	CH_2_Cl_2_	−340	−350	−341	−338	−333	−350.1 [45]
[Ti(SnMe_3_‐Cp)Br_3_]	CH_2_Cl_2_	−59	−76	−60	−53	−46	−95.4 [45]
[Ti((SiMe_3_)_2_‐Cp)Cl_3_]	CH_2_Cl_2_	−304	−313	−305	−299	−294	−332 [118]
[Ti((SiMe_3_)_3_‐Cp)Cl_3_]	CH_2_Cl_2_	−255	−265	−256	−248	−243	−298 [118]
[Ti(Cp)_2_F_2_]	CH_2_Cl_2_	−1143	−1146	−1143	−1150	−1144	−1036.5 [44]
MAD (ppm)	—	48	48	48	45	48	—
SD_AD_ (ppm)	—	57	58	56	53	53	—
MRD	—	11.3%	9.5%	11.1%	11.7%	12.9%	—
SD_RD_	—	10.1%	8.1%	9.7%	12.1%	14.5%	—

*Note:* References to experimental values are given in square brackets.

**FIGURE 4:**
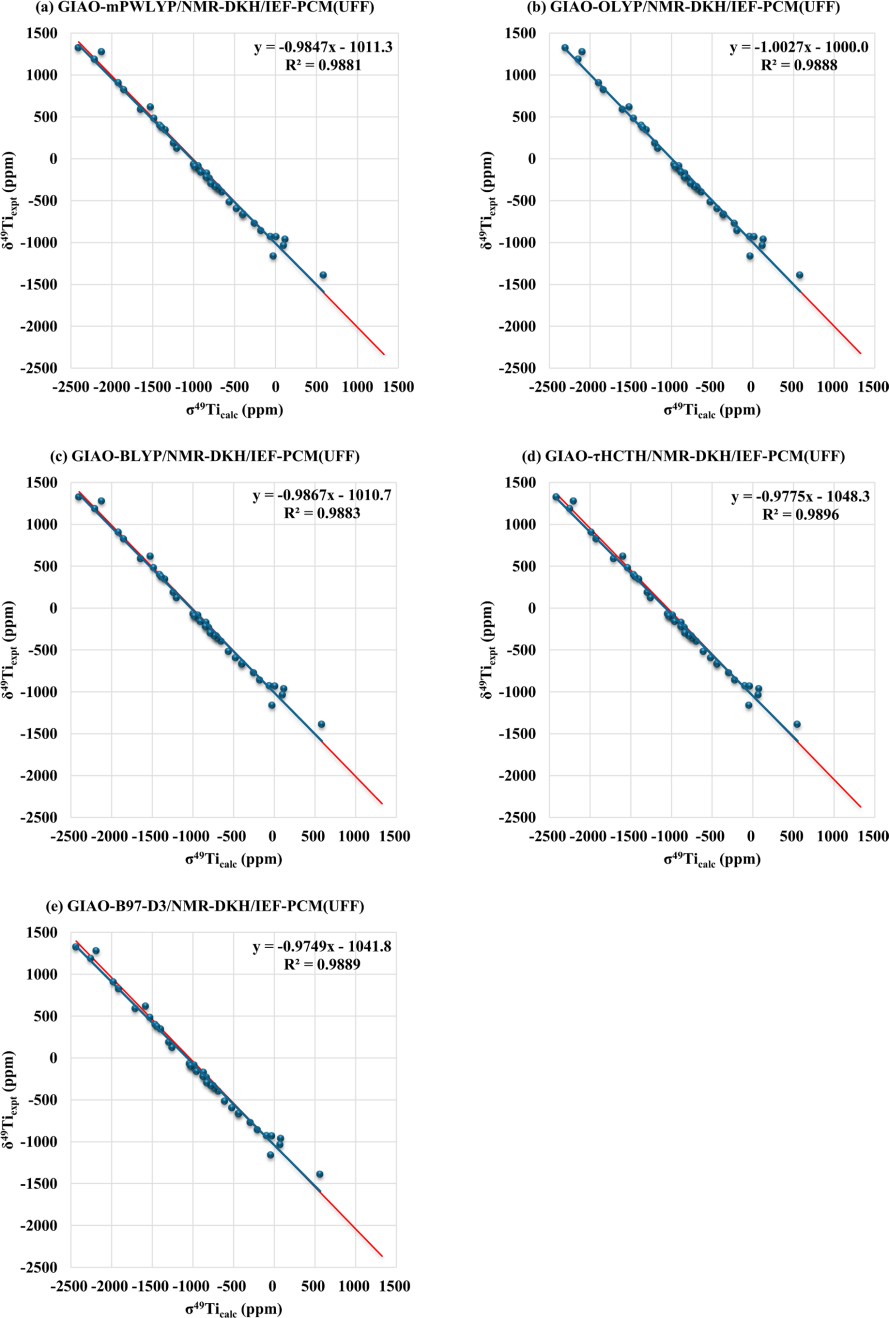
Linear regression models obtained from the correlation between σ^49^Ti_calc_ x δ^49^Ti_expt_ at GIAO‐DFT‐Functional/NMR‐DKH/IEF‐PCM(UFF), considering the mPWLYP, OLYP, BLYP, τHCTH, and B97‐D3(BJ) functionals, for a set of 41 Ti(IV) complexes. In red, the line *y* = *ax* + *b* with a slope of 45° is shown for better visualization of the errors obtained with the proposed computational protocols.

Furthermore, from the correlation between the calculated σ^49^Ti and experimental δ^49^Ti (σ^49^Ti_calc_ × δ^49^Ti_expt_), a coefficient of determination (*R* ^2^) greater than 0.99 with an angular coefficient close to 1 was found (Figure 4), showing that the proposed protocols are statistically consistent.

The range of experimental chemical shift values for the 41 Ti(IV) complexes studied varies between −1389 and + 1325 ppm, that is, a very wide range. In general, the best way to evaluate the quality of the computational protocols is through the absolute deviations, since the relative deviations can generate a distortion due to δ^49^Ti values close to zero, that is, in the complexes where the ^49^Ti nucleus is more shielded. Since the MAD for all the protocols considered is very similar, the mean relative deviation (MRD) can be a way to rank the protocols. Thus, from the calculated values, MRD varies between 9.5% (OLYP) and 12.9% (B97‐D3BJ). Therefore, considering that the OLYP functional presented a MAD and a SD of the absolute deviations similar to the other protocols, the lowest MRD, and presents a lower computational cost when compared to the τHCTH functional (lower MAD), the GIAO‐OLYP/NMR‐DKH/IEF‐PCM(UFF) protocol can be considered a good alternative for the description of δ^49^Ti in Ti(IV) complexes. In addition to this, it is also found in the literature that the OLYP functional represents an excellent choice combining quality and low computational cost [119].

**FIGURE 5:**
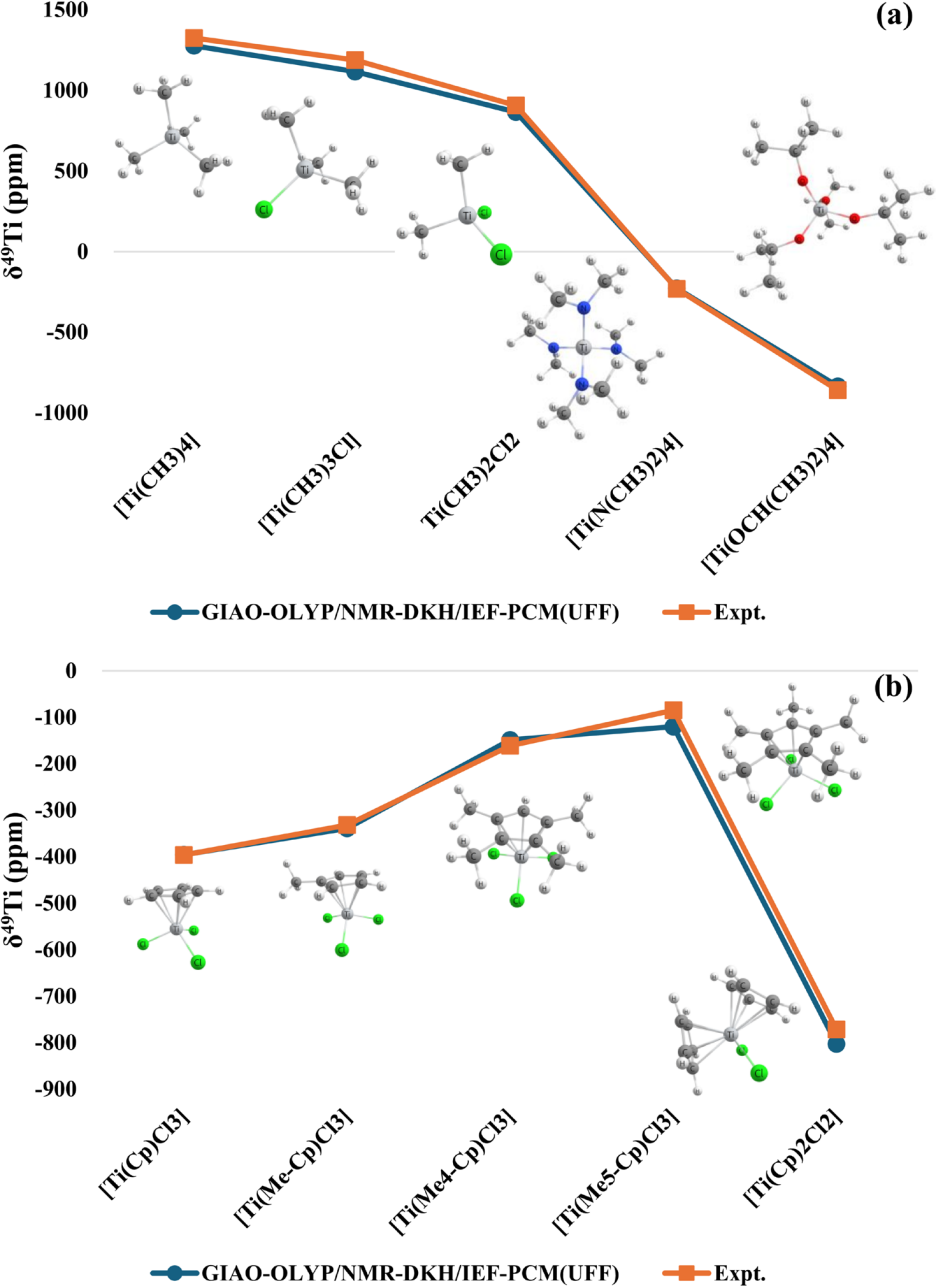
Calculated δ^49^Ti at GIAO‐OLYP/NMR‐DKH/IEF‐PCM(UFF) level for two groups of Ti(IV) complexes studied in the present paper.

An analysis of the trend of the calculated values in relation to the experimental values was performed with the GIAO‐OLYP/NMR‐DKH/IEF‐PCM(UFF) protocol for two sets of selected complexes. In the first set, the complexes [Ti(CH_3_)_4_], [Ti(CH_3_)_3_Cl], [Ti(CH_3_)_2_Cl_2_], [Ti(N(CH_3_)_2_)_4_], and [Ti(OCH(CH_3_)_2_)_4_] were analyzed, that is, it is possible to evaluate whether the computational protocol is capable of describing the variation of four Ti–C bonds, passing through the replacement of these bonds by one Ti–Cl bond, two Ti–Cl bonds, four Ti–N bonds, and four Ti–O bonds. The experimental values of δ^49^Ti for this group range from +1325 to −859. From the results obtained (Figure 5a) it can be observed that the protocol was able to adequately describe the trend observed experimentally for these complexes.

The second set analyzed involves a series of complexes with the Cp ligand; the complexes [Ti(Cp)Cl_3_], [Ti(Me‐Cp)Cl_3_], [Ti(Me_4_‐Cp)Cl_3_], [Ti(Me_5_‐Cp)Cl_3_], and [Ti(Cp)_2_Cl_2_] were selected, which present δ^49^Ti values ranging from −85 ppm to −772 ppm. In this group, Ti is observed coordinated to a Cp ligand without substitution and substituted by one, four, and five methyl groups, in addition to considering the complex with two Cp ligands. Again, it can be observed (Figure 5b) that the trend of the experimental values was adequately followed by the calculated values.

Finally, in the literature, we find the papers of Bühl and Mauschick [22], who studied the δ^49^Ti of 10 Ti(IV) complexes, having in their best protocol a MAD of 110 ppm, and the paper of Koch and Bruhn [23], who studied 14 Ti(IV) complexes, having in their best protocol a MAD of 67 ppm. Finally, Schattenberg et al. [24] at GIAO‐cM06L/pcSseg‐3//BP86‐D3(BJ)/def2‐TZVPD/def2‐TZVP found a MAD of 45 ppm for a set of 12 Ti(IV) complexes. In the present study, we show for a larger set of 41 Ti(IV) complexes, a MAD of only 45 ppm with the GIAO‐OLYP/NMR‐DKH/IEF‐PCM(UFF) protocol and 48 ppm with the GIAO‐τHCTH/NMR‐DKH/IEF‐PCM(UFF) protocol, confirming the robustness and applicability of the protocols developed with the NMR‐DKH basis sets, with a lower computational cost. However, an interesting result in both Bühl and Mauschick [22] and Koch and Bruhn [23] papers was obtained for the [TiI_4_] complex, with both studies presenting an AD lower than 100 ppm for this complex when a hybrid functional was employed. The presence of ligand I, a ligand with high polarizability, may require inclusion of the Hartree–Fock exchange term, justifying the best result found in the literature. Thus, for this complex we calculated the δ^49^Ti in the [TiI_4_] complex with the value of 1219 ppm (AD = 59 ppm) using the GIAO‐O3LYP/NMR‐DKH/IEF‐PCM(UFF) protocol. Thus, we can conclude that for complexes that have the Ti–I bond, the use of the O3LYP hybrid functional is the best option.

Therefore, the results of the present paper indicate that the GIAO‐OLYP/NMR‐DKH/IEF‐PCM(UFF) protocol is an excellent alternative for the study of δ^49^Ti in Ti(IV) complexes, balancing a good description of the property with a low computational cost, with the hybrid O3LYP functional being a good alternative for complexes with Ti–I bonds.

#### 3.1.4 The Role of the Relativistic Effects

Calculations with a one‐component (1c) nonrelativistic Hamiltonian at the BLYP/dyall.VDZ level and with a 4c relativistic Hamiltonian at the 4c‐BLYP/dyall.VDZ level were performed with the ReSpect 5.3.0 program [111] to describe the δ^49^Ti of the 41 Ti(IV) complexes studied in the development of computational protocols (Table S2).

**FIGURE 6:**
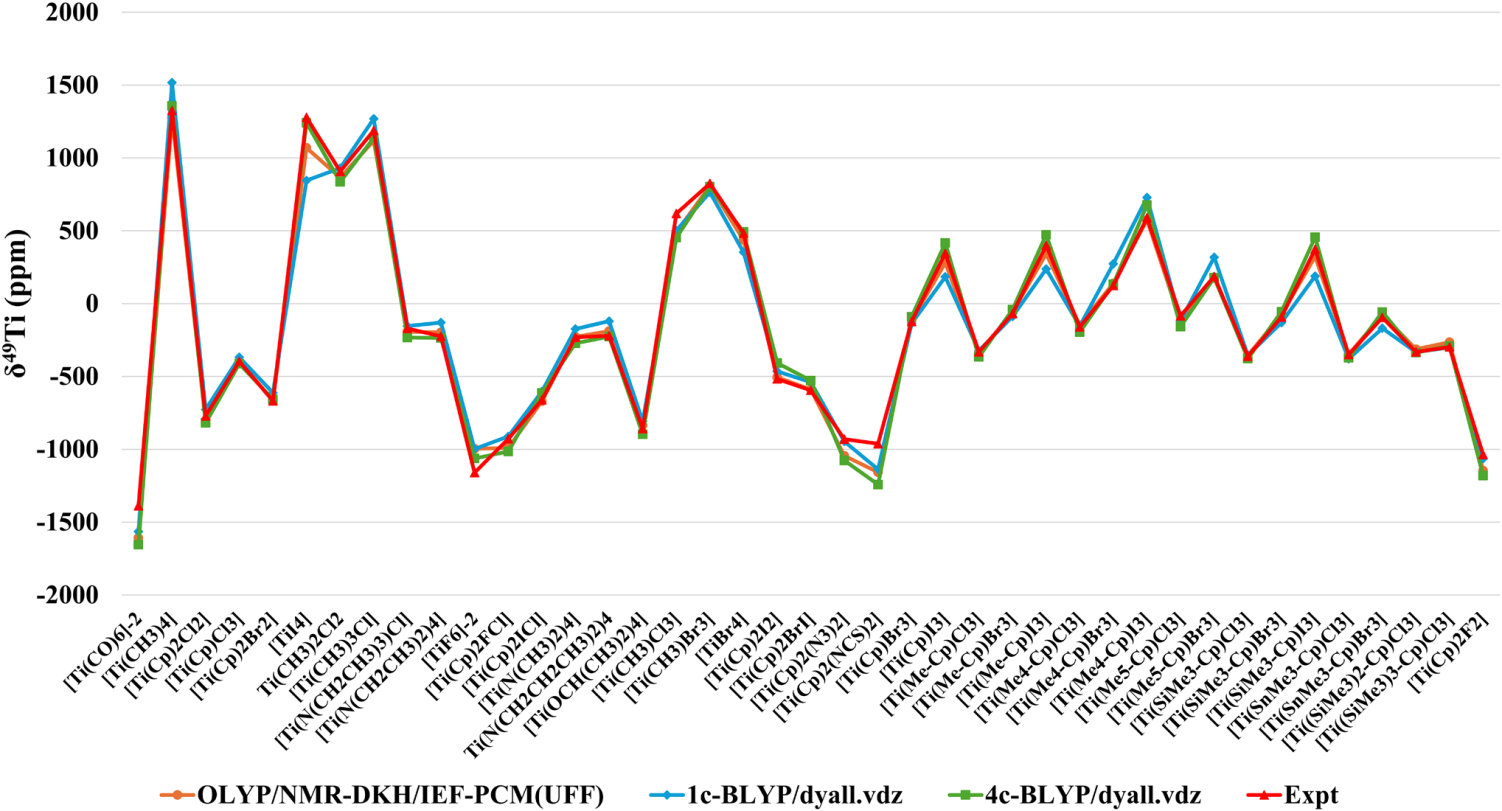
Assessment of the role of the relativistic effects in predicting the δ^49^Ti for 41 Ti(IV) complexes.

From the results obtained (Figure 6), it is observed that the inclusion of relativistic effects results in a better description of the δ^49^Ti, with the MAD decreasing from 82 ppm (1c nonrelativistic Hamiltonian) to 62 ppm (4c relativistic Hamiltonian). Relativistic effects, mainly those arising from spin–orbit coupling, played a more significant role in the complexes with three and four heavy nuclei coordinated to Ti. For example, for the complexes [TiI_4_], [TiBr_4_], and [Ti(Cp)I_3_], the AD decreased from 433, 130, and 161 ppm (nonrelativistic—1c) to 37, 9, and 71 ppm (relativistic—4c), respectively.

However, it is observed that when the protocol with the nonrelativistic Hamiltonian GIAO‐OLYP/NMR‐DKH/IEF‐PCM(UFF) is employed (Figure 6), MAD of 48 ppm is found. The AD for the [TiI_4_], [TiBr_4_], and [Ti(Cp)I_3_] complexes was 209, 46, and 65 ppm, respectively. Only for the [TiI_4_] complex was a higher AD found (209 ppm), which can also be justified, in part, by the need to include a percentage of HF exchange, as observed for the AD of only 59 ppm found with the GIAO‐O3LYP/NMR‐DKH/IEF‐PCM(UFF) protocol. The results show that although full 4c relativistic Hamiltonian calculations provide the most complete description of relativistic effects, the nonrelativistic protocol based on NMR‐DKH basis sets achieves comparable results for predicting the δ^49^Ti, being an efficient and lower computational cost alternative, mainly in complexes where spin–orbit effects are not very relevant or tend to cancel each other out.

Then, the protocol with the nonrelativistic Hamiltonian at the GIAO‐OLYP/NMR‐DKH/IEF‐PCM(UFF) level, with a MAD of only 48 ppm, presents itself as an excellent option for predicting δ^49^Ti, combining good agreement with experimental data and low computational cost.

#### 3.1.5 Predictive Model Evaluation and External Validation

The calculated results obtained with the computational protocol employing a nonrelativistic Hamiltonian showed good agreement with the experimental values. However, it can be argued that this performance is partly due to fortuitous error cancellation. To evaluate this possibility and verify whether the quality of the calculated results remains in a more robust scenario, a predictive model based on linear regression, Equation (6), was developed, considering only the protocol at the GIAO‐OLYP/NMR‐DKH/IEF‐PCM(UFF) level. The model was built from the set of 41 studied molecules (4 from the initial protocol and 37 additional ones) and allows systematic correction for effects not considered in the QM protocol, functioning as a statistical refinement step. However, it is important to emphasize that such regression does not increase the intrinsic theoretical precision of the QM method; it only improves the apparent agreement with the experimental values. The validity of the model was then tested on an external set of nine Ti(IV) complexes, allowing for the evaluation of its extrapolation capacity and the actual consistency of the proposed protocol.

From the linear regression between σ^49^Ti_calc_ × δ^49^Ti_expt_, Figure 4b, Equation (6) was found, which can be used for predicting the δ^49^Ti.

(6) δTicalc49=−1.0027×σcalc−1000.0

As stated previously, the slope close to −1.0 indicates that the model is physically consistent; from Equation (6) *a* = −1.0027, indicating a good model.

From the calculated values of δ^49^Ti with Equation (6), Table S2, a MAD of only 50 ppm is observed, similar to the value of 48 ppm obtained considering Equation (3), indicating that the predictive model does not distort the results obtained from the direct calculation of the chemical shift, reinforcing that the linear regression model does not distort the raw results, but neither does it make them intrinsically more accurate from a theoretical point of view.

**FIGURE 7:**
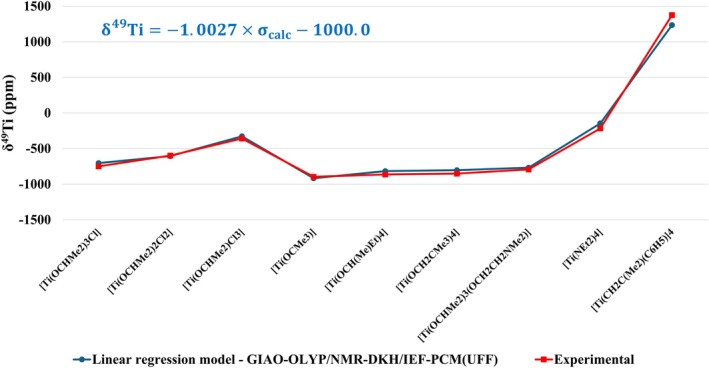
Calculated δ^49^Ti at GIAO‐OLYP/NMR‐DKH/IEF‐PCM(UFF) level with the linear regression model, δTi49=−1.0027×σcalc−1000.0, for the set of nine Ti(IV) complexes used for external validation of predictive model.

Subsequently, the predictive model was applied to a set of nine external Ti(IV) complexes not used in the model development. The calculated results (Figure 7) showed a MAD of only 48 ppm, demonstrating that the model maintained the quality of its estimates, confirming its robustness, and that the good agreement obtained between the calculated and experimental values is not due solely to error cancellation. In other words, the model was able to recover at least partially aspects not explicitly included in the nonrelativistic Hamiltonian.

It is also important to highlight that the δ^49^Ti directly calculated (Equation 3) also showed good agreement with the experimental data (Table 4). Thus, predictive modeling can be seen as a complement that reinforces the consistency and extrapolation capacity of the protocol, but not as an essential requirement for obtaining good results or as a means of increasing the intrinsic theoretical accuracy of the method.

**TABLE 4:** Calculated δ^49^Ti (ppm) at GIAO‐OLYP/NMR‐DKH/IEF‐PCM(UFF) level considering Equations (3) and (6) for the set of nine Ti(IV) complexes used in the external validation.

Ti(IV) complexes	Solvent	Equation (3)	Equation (6)	Expt.
[Ti(OCHMe_2_)_3_Cl]	CHCl_3_	−730	−703	−749 [117]
[Ti(OCHMe_2_)_2_Cl_2_]	CHCl_3_	−632	−605	−598 [117]
[Ti(OCHMe_2_)Cl_3_]	CHCl_3_	−354	−327	−358 [117]
[Ti(OCMe_3_)]	CHCl_3_	−943	−916	−896 [117]
[Ti(OCH(Me)Et)_4_]	CHCl_3_	−842	−816	−863 [117]
[Ti(OCH_2_CMe_3_)_4_]	CHCl_3_	−830	−803	−851 [117]
[Ti(OCHMe_2_)_3_(OCH_2_CH_2_NMe_2_)]	CHCl_3_	−795	−768	−792 [117]
[Ti(NEt_2_)_4_]	C_6_H_6_	−174	−146	−216 [117]
[Ti(CH_2_C(Me_2_) (C_6_H_5_))]_4_	C_6_H_6_	1203	1235	1375 [117]
MAD (ppm)	—	40	48	—
SD_AD_ (ppm)	—	48	37	—

*Note:* References to experimental values are given in square brackets. Equation (3) = δTi49=σref−σcalc. Equation (6) = δTi49=−1.0027×σcalc−1000.

Thus, the use of the predictive model, Equation (6), expands the applicability of the proposed protocol at the GIAO‐OLYP/NMR‐DKH/IEF‐PCM(UFF) level, proving to be a robust protocol that can be applied in future studies involving Ti(IV) complexes.

## 4 Concluding Remarks

The present study aimed to propose computational protocols based on the DFT level with the NMR‐DKH basis sets to calculate the Ti‐49 NMR chemical shift (δ^49^Ti) in Ti(IV) complexes.

Initially, a new NMR‐DKH basis set, with a TZ2P character, was proposed for the Ti atom. Then, a set of four Ti(IV) complexes, with δ^49^Ti ranging from −1389 to +1325 ppm, and the internal reference in Ti‐49 NMR were selected for the DFT benchmarking, which included 55 DFT functionals for predicting the nuclear shielding tensor. All geometries were optimized at the BLYP/def2‐SVP/IEF‐PCM(UFF) level and the NMR prediction was carried out considering the GIAO‐DFT‐Functional/NMR‐DKH/IEF‐PCM(UFF) level, both employing a nonrelativistic Hamiltonian.

From the calculated results, it was observed that overall, the GGA functionals presented the best description of δ^49^Ti. The functionals that presented a mean absolute deviation (MAD) from the experimental values lower than 80 ppm—GGA mPWLYP, BLYP, and OLYP, meta‐GGA τHCTH, and GGA with dispersion B97‐D3BJ—were selected for application in other 37 Ti(IV) complexes.

Considering the calculated values with the five best computational protocols for the set of 41 Ti(IV) complexes (4 initials +37 validation), an excellent agreement was observed with the experimental data. All protocols were able to adequately describe the trends observed in the experimental values, presenting MAD of 45 ppm (τHCTH) and 48 ppm (OLYP, mPWLYP, BLYP, and B97‐D3(BJ)) and a standard deviation lower than ~50 ppm, indicating that the calculated deviations are homogeneous. These results also reinforce the quality of the NMR‐DKH basis sets, which presented an excellent description regardless of the DFT functional considered.

The OLYP functional, which also exhibits the lowest mean relative deviation (MRD = 9.5%), indicating that it presents the best description of δ^49^Ti close to zero, and a lower computational cost, was indicated as an excellent choice for the study of ^49^Ti NMR.

Calculations with a full 4c relativistic Hamiltonian at the GIAO‐4c‐BLYP/dyall.VDZ level were also performed to evaluate the role of relativistic effects in predicting δ^49^Ti. The results showed that, overall, the inclusion of relativistic effects results in a better description of the property. In complexes where spin–orbit contributions are small or tend to cancel, protocols based on nonrelativistic Hamiltonians with the NMR‐DKH basis sets have shown comparable performance, presenting themselves as an efficient and lower computational cost alternative for predicting the δ^49^Ti in Ti(IV) complexes. However, for complexes containing ligands or heavy atoms (e.g., [TiI_4_] and analogues), the explicit inclusion of spin–orbit interactions is essential for a reliable quantitative description.

Predictive modeling using linear regression confirmed statistical consistency between the calculated and experimental values, maintaining good agreement in both the developing set (41 complexes) and the external set of nine complexes. These results reinforce the robustness of the protocol and expand its extrapolation capability, without attributing the good performance solely to error cancellation. However, it is important to highlight that linear regression constitutes only a statistical calibration; that is, it does not increase the intrinsic theoretical precision of the QM computational protocol.

Thus, the NMR computational protocol: GIAO‐OLYP/NMR‐DKH/IEF‐PCM(UFF)//Opt‐BLYP/def2‐SVP/IEF‐PCM(UFF) was recommended as an excellent alternative for the study of δ^49^Ti in Ti(IV) complexes. Additionally, the proposed linear regression, δTicalc49=−1.0027×σcalc−1000.0, provides a complementary predictive model, capable of expanding the applicability of the protocol and reinforcing its robustness for extrapolation to new Ti(IV) complexes.

## Conflicts of Interest

The authors declare no conflicts of interest.

## Supporting information


**Data S1:** jcc70258‐sup‐0001‐supinfo.docx.
**Table S1:** Calculated δ^49^Ti (ppm) at GIAO‐DFT‐Functional/NMR‐DKH/IEF‐PCM(UFF) levels for the Ti(IV) complexes selected for the construction of the computational protocol. The mean absolute deviation (MAD, ppm) in relation to the experiment values obtained with each DFT Functional also are included.
**Table S2:** Calculated δ^49^Ti (ppm) considering nonrelativistic and relativistic Hamiltonians for a set of 41 Ti(IV) complexes.

## Data Availability

The data that support the findings of this study are available from the corresponding author upon reasonable request.
