# Supplementary material for: Predicting Ti‐49 NMR Chemical Shift With New NMR‐DKH Basis Set
Source: J Comput Chem. 2025 Nov 2;46(29):e70258. doi: 10.1002/jcc.70258 (PMC12580593; doi:10.1002/jcc.70258)
Supplement: Supplementary file 1 — Data S1: jcc70258‐sup‐0001‐supinfo.docx. Table S1: Calculated δ49Ti (ppm) at GIAO‐DFT‐Functional/NMR‐DKH/IEF‐PCM(UFF) levels for the Ti(IV) complexes selected for the construction of the computational protocol. The mean absolute deviation (MAD, ppm) in relation to the experiment values obtained with each DFT Functional also are included. Table S2: Calculated δ49Ti (ppm) considering nonrelativistic and relativistic Hamiltonians for a set of 41 Ti(IV) complexes. [file JCC-46-0-s001.docx]

**Predicting Ti-49 NMR chemical shift with new NMR-DKH basis set**

Matheus Gunar Ramalho Gomes^1,2^, Catherine Rodrigues Siqueira de Souza^2^, Diego Fernando da Silva Paschoal^2*^, Wagner Batista De Almeida^1^

^1^LQC-MM – Laboratório de Química Computacional e Modelagem Molecular - Instituto de Química, Universidade Federal Fluminense - Outeiro de São João Batista, S/N, Niterói, RJ, Brazil.

^2^NQTCM: Núcleo de Química Teórica e Computacional de Macaé, Polo Ajuda, Instituto Multidisciplinar de Química, Centro Multidisciplinar UFRJ-Macaé, Universidade Federal do Rio de Janeiro, 27.971-525, Macaé, RJ, Brazil.

_____________________

*Corresponding author:

Diego F. S. Paschoal, e-mail: diegopaschoal01@gmail.com or diegofspaschoal@macae.ufrj.br

**Supplementary Material**

**NMR-DKH basis set for the Ti atom (GAUSSIAN PROGRAM FORMAT)**

Ti 0

S 7 1.00

131685.903249881 0.00144

52674.3612999524 0.00018

21069.7445199809 0.00392

8427.89780799240 0.00658

3371.15912319695 0.02036

1348.46364927878 0.05393

539.385459711512 0.14253

S 1 1.00

215.754183884605 1.00000

S 1 1.00

86.3016735538420 1.00000

S 1 1.00

34.5206694215368 1.00000

S 1 1.00

13.8082677686147 1.00000

S 1 1.00

5.52330710744588 1.00000

S 1 1.00

2.20932284297835 1.00000

S 1 1.00

0.88372913719134 1.00000

S 1 1.00

0.35349165487654 1.00000

S 1 1.00

0.14139666195062 1.00000

S 1 1.00

0.05655866478025 1.00000

S 1 1.00

0.02262346591210 1.00000

P 6 1.00

1375.44747973736 0.00184

500.162719904494 0.00608

181.877352692543 0.03293

66.1372191609249 0.13264

24.0498978766999 0.38028

8.74541740970910 0.49933

P 1 1.00

3.18015178534875 1.00000

P 1 1.00

1.15641883103591 1.00000

P 1 1.00

0.42051593855851 1.00000

P 1 1.00

0.15291488674855 1.00000

P 1 1.00

0.05560541336311 1.00000

D 4 1.00

24.0073133461147 0.02123

8.00243778203820 0.09300

2.66747926067941 0.29907

0.88915975355980 0.46515

D 1 1.00

0.29638658451994 1.00000

D 1 1.00

0.09879552817331 1.00000

F 2 1.00

4.57900000000000 0.00253

1.30020000000000 0.01002

F 1 1.00

0.42630000000000 1.00000

****

**Table S1.** Calculated δ^49^Ti (ppm) at GIAO-DFT-Functional/NMR-DKH/IEF-PCM(UFF) levels for the Ti(IV) complexes selected for the construction of the computational protocol. The mean absolute deviation (MAD, ppm) in relation to the experiment values obtained with each DFT Functional also are included.

| **DFT-Functionals** | | **[Ti(CO)_6_]^-2^** | **[Ti(CH_3_)_4_]** | **[Ti(Cp)_2_Cl_2_]** | **[Ti(Cp)Cl_3_]** | **MAD** |
| --- | --- | --- | --- | --- | --- | --- |
| LDA | SVWN | -1657 | 1559 | -843 | -417 | 148 |
| GGA | mPWLYP | -1630 | 1365 | -787 | -388 | 76 |
|  | mPWPBE | -1618 | 1381 | -816 | -403 | 84 |
|  | mPWPW91 | -1619 | 1380 | -814 | -402 | 83 |
|  | OLYP | -1608 | 1277 | -803 | -396 | 75 |
|  | BLYP | -1626 | 1360 | -787 | -389 | 74 |
|  | XLYP | -1669 | 1622 | -847 | -418 | 169 |
|  | PW91 | -1622 | 1389 | -814 | -402 | 86 |
|  | BPW91 | -1615 | 1374 | -814 | -402 | 81 |
|  | BP86 | -1619 | 1396 | -812 | -402 | 86 |
|  | PBE | -1616 | 1384 | -815 | -403 | 84 |
|  | PBEh | -1617 | 1400 | -811 | -402 | 87 |
|  | OPBE | -1598 | 1285 | -833 | -411 | 82 |
|  | SOGGA11 | -1633 | 1223 | -796 | -386 | 95 |
| meta-GGA | VSXC | -1500 | 1213 | -620 | -293 | 120 |
|  | BB95 | -1628 | 1472 | -809 | -398 | 106 |
|  | TPSS | -1581 | 1229 | -808 | -400 | 82 |
|  | revTPSS | -1578 | 1233 | -820 | -407 | 85 |
|  | M06L | -1474 | 1156 | -729 | -355 | 84 |
|  | M11L | -1389 | 843 | -632 | -313 | 177 |
|  | MN12L | -1418 | 788 | -753 | -384 | 149 |
|  | MN15L | -1400 | 932 | -728 | -362 | 121 |
|  | τHCTH | -1631 | 1333 | -788 | -389 | 68 |
| Hybrid | mPW1LYP | -1634 | 1330 | -874 | -439 | 98 |
|  | mPW3PBE | -1630 | 1352 | -886 | -444 | 107 |
|  | mPW1PW91 | -1620 | 1339 | -902 | -453 | 108 |
|  | O3LYP | -1623 | 1271 | -845 | -420 | 96 |
|  | B3LYP | -1638 | 1338 | -861 | -431 | 97 |
|  | X3LYP | -1638 | 1341 | -867 | -435 | 100 |
|  | B3PW91 | -1628 | 1347 | -884 | -443 | 105 |
|  | B3P86 | -1631 | 1365 | -882 | -442 | 109 |
|  | PBE0 | -1617 | 1344 | -903 | -454 | 109 |
|  | PBE0h | -1618 | 1355 | -900 | -453 | 111 |
|  | BHandH | -1584 | 1457 | -981 | -502 | 161 |
|  | BhandHLYP | -1552 | 1312 | -936 | -482 | 106 |
|  | SOGGA11X | -1570 | 1339 | -938 | -479 | 111 |
|  | B97-2 | -1626 | 1313 | -875 | -438 | 98 |
| Hybrid meta-GGA | B1B95 | -1633 | 1429 | -910 | -455 | 136 |
|  | TPSSh | -1589 | 1226 | -842 | -420 | 98 |
|  | BMK | -1608 | 1463 | -1022 | -508 | 180 |
|  | PW6B95 | -1636 | 1419 | -900 | -449 | 131 |
|  | M06HF | -1634 | 2081 | -1218 | -664 | 429 |
|  | M06 | -1614 | 1689 | -881 | -439 | 185 |
|  | M062x | -1572 | 1651 | -945 | -480 | 191 |
|  | MN15 | -1772 | 1986 | -994 | -489 | 340 |
|  | τHCTHhyb | -1648 | 1352 | -870 | -432 | 105 |
| LRC | ωB97 | -1573 | 1379 | -1026 | -535 | 158 |
|  | ωB97x | -1583 | 1378 | -994 | -516 | 147 |
|  | LC-BLYP | -1608 | 1419 | -1075 | -559 | 195 |
|  | LC-ωPBE | -1591 | 1354 | -1046 | -540 | 162 |
|  | CAM-B3LYP | -1622 | 1380 | -952 | -485 | 139 |
|  | M11 | -1601 | 1390 | -1054 | -558 | 180 |
|  | MN12SX | -1457 | 986 | -813 | -417 | 117 |
| Dispersion | ωB97xD | -1602 | 1358 | -963 | -496 | 134 |
|  | B97-D3(BJ) | -1635 | 1366 | -779 | -384 | 77 |
|  | Experimental | -1389 [42] | 1325 [43] | -772 [44] | - | - |

**Table S2.** Calculated δ^49^Ti (ppm) considering nonrelativistic and relativistic Hamiltonians for a set of 41 Ti(IV) complexes.

| **Ti(IV) complexes** | **Solvent** | **Model 1 NMR-DKH** | **Model 2 Predictive** | **Model 3**  **1c** | **Model 4**  **4c** | **Expt.** |
| --- | --- | --- | --- | --- | --- | --- |
| [Ti(CO)_6_]^-2^ | CH_3_CN | -1608 | -1584 | -1565 | -1655 | -1389 [42] |
| [Ti(CH_3_)_4_] | CHCl_3_ | 1277 | 1309 | 1517 | 1357 | 1325 [43] |
| [Ti(Cp)_2_Cl_2_] | CH_2_Cl_2_ | -803 | -776 | -728 | -819 | -772 [44] |
| [Ti(Cp)Cl_3_] | CHCl_3_ | -396 | -369 | -368 | -412 | -396.5 [45] |
| [Ti(Cp)_2_Br_2_] | CH_3_CN | -664 | -637 | -612 | -657 | -668.3 [44] |
| [TiI_4_] | C_6_H_6_ | 1069 | 1100 | 845 | 1241 | 1278.3 [44] |
| [Ti(CH_3_)_2_Cl_2_] | CH_2_Cl_2_ | 865 | 896 | 930 | 835 | 907 [43] |
| [Ti(CH_3_)_3_Cl] | CH_2_Cl_2_ | 1117 | 1148 | 1269 | 1136 | 1188 [44] |
| [Ti(N(CH_2_CH_3_)_3_)Cl] | CHCl_3_ | -192 | -164 | -154 | -233 | -171 [117] |
| [Ti(N(CH_2_CH_3_)_2_)_4_] | CH_2_Cl_2_ | -197 | -169 | -130 | -236 | -224 [117] |
| [TiF_6_]^-2^ | H_2_O | -997 | -971 | -998 | -1063 | -1160.9 [118] |
| [Ti(Cp)_2_FCl] | CH_2_Cl_2_ | -991 | -965 | -912 | -1015 | -928.3 [44] |
| [Ti(Cp)_2_ICl] | CH_2_Cl_2_ | -671 | -644 | -603 | -614 | -661.2 [44] |
| [Ti(N(CH_3_)_2_)_4_] | CH_2_Cl_2_ | -227 | -199 | -174 | -272 | -231 [117] |
| Ti(N(CH_2_CH_2_CH_3_)_2_)_4_ | CH_2_Cl_2_ | -189 | -161 | -121 | -226 | -221 [117] |
| [Ti(OCH(CH_3_)_2_)_4_] | CH_2_Cl_2_ | -835 | -808 | -808 | -897 | -859 [117] |
| [Ti(CH_3_)Cl_3_] | CH_2_Cl_2_ | 491 | 521 | 502 | 453 | 618 [117] |
| [Ti(CH_3_)Br_3_] | CH_2_Cl_2_ | 808 | 839 | 764 | 802 | 825 [117] |
| [TiBr_4_] | CH_2_Cl_2_ | 437 | 467 | 353 | 492 | 482.9 [44] |
| [Ti(Cp)_2_I_2_] | CH_2_Cl_2_ | -506 | -479 | -464 | -407 | -517.2 [44] |
| [Ti(Cp)_2_BrI] | CH_2_Cl_2_ | -589 | -562 | -539 | -529 | -595.1 [44] |
| [Ti(Cp)_2_(N_3_)_2_] | CHCl_3_ | -1045 | -1019 | -946 | -1077 | -930.9 [44] |
| [Ti(Cp)_2_(NCS)_2_] | CH_2_Cl_2_ | -1158 | -1132 | -1138 | -1242 | -962.3 [44] |
| [Ti(Cp)Br_3_] | CHCl_3_ | -117 | -88 | -136 | -91 | -123.8 [45] |
| [Ti(Cp)I_3_] | CH_2_Cl_2_ | 280 | 310 | 184 | 416 | 345.2 [45] |
| [Ti(Me-Cp)Cl_3_] | CH_2_Cl_2_ | -339 | -311 | -323 | -366 | -332 [45] |
| [Ti(Me-Cp)Br_3_] | CH_2_Cl_2_ | -60 | -32 | -89 | -42 | -68.8 [45] |
| [Ti(Me-Cp)I_3_] | CH_2_Cl_2_ | 343 | 372 | 238 | 472 | 399.5 [45] |
| [Ti(Me_4_-Cp)Cl_3_] | CH_2_Cl_2_ | -149 | -120 | -155 | -195 | -161.1 [45] |
| [Ti(Me_4_-Cp)Br_3_] | CH_2_Cl_2_ | 139 | 168 | 273 | 133 | 123.5 [45] |
| [Ti(Me_4_-Cp)I_3_] | CH_2_Cl_2_ | 574 | 604 | 728 | 678 | 589.4 [45] |
| [Ti(Me_5_-Cp)Cl_3_] | CHCl_3_ | -120 | -92 | -118 | -158 | -85 [45] |
| [Ti(Me_5_-Cp)Br_3_] | CHCl_3_ | 175 | 204 | 318 | 178 | 186.7 [45] |
| [Ti(SiMe_3_-Cp)Cl_3_] | CH_2_Cl_2_ | -355 | -328 | -355 | -378 | -361.4 [45] |
| [Ti(SiMe_3_-Cp)Br_3_] | CH_2_Cl_2_ | -77 | -48 | -130 | -56 | -93.2 [45] |
| [Ti(SiMe_3_-Cp)I_3_] | CH_2_Cl_2_ | 323 | 353 | 188 | 456 | 373.5 [45] |
| [Ti(SnMe_3_-Cp)Cl_3_] | CH_2_Cl_2_ | -350 | -322 | -379 | -373 | -350.1 [45] |
| [Ti(SnMe_3_-Cp)Br_3_] | CH_2_Cl_2_ | -76 | -48 | -169 | -58 | -95.4 [45] |
| [Ti((SiMe_3_)_2_-Cp)Cl_3_] | CH_2_Cl_2_ | -313 | -285 | -333 | -336 | -332 [119] |
| [Ti((SiMe_3_)_3_-Cp)Cl_3_] | CH_2_Cl_2_ | -265 | -237 | -302 | -291 | -298 [119] |
| [Ti(Cp)_2_F_2_] | CH_2_Cl_2_ | -1146 | -1121 | -1065 | -1182 | -1036.5 [44] |
|  |  |  |  |  |  |  |
| MAD (ppm) | - | 48 | 50 | 82 | 62 | - |
| SD_AD_ (ppm) | - | 58 | 48 | 81 | 62 | - |

Model 1: GIAO-OLYP/NMR-DKH/IEF-PCM(UFF) – GAUSSIAN 16 Rev. C.01 - $\delta{}^{49}\mathrm{Ti}=\sigma_{\mathrm{ref}}-\sigma_{\mathrm{calc}}$

Model 2: Predictive model with the protocol GIAO-OLYP/NMR-DKH/IEF-PCM(UFF) – GAUSSIAN 16 Rev. C.01 - $\delta{}^{49}\mathrm{Ti}=-1.0027\times\sigma_{\mathrm{calc}}-1000$

Model 3: GIAO-1c-BLYP/dyall.VDZ – ReSpect 5.3.0 - $\delta{}^{49}\mathrm{Ti}=\sigma_{\mathrm{ref}}-\sigma_{\mathrm{calc}}$

Model 4: GIAO-4c-BLYP/dyall.VDZ – ReSpect 5.3.0 - $\delta{}^{49}\mathrm{Ti}=\sigma_{\mathrm{ref}}-\sigma_{\mathrm{calc}}$

**References:**

[42] Chi, K. M., Frerichs, S. R., Philson, S. B., Ellis, J. E., *J. Am. Chem. Soc.*, **1988**, 110, 303–304.

[43] Berger, S., Bock, W., Frenking, G., Jonas, V., Mueller, F., *J. Am. Chem. Soc.*, **1995**, 117, 3820–3829.

[44] Hao, N., Sayer, B. G., Dénès, G., Bickley, D. G., Detellier, C., McGlinchey, M. J., J. *Magn. Reson.*, **1982**, 50, 50–63.

[45] Erben, M., Růžička, A., Picka, M., Pavlík, I., *Magn. Reson. Chem.*, **2004**, 42, 414–417.

[117] Berger, S., Bock, W., Marth, C. F., Raguse, B., Reetz, M. T., *Magn. Reson. Chem.*, **1990**, *28*, 559–560.

[118] Foris, A., *Magn. Reson. Chem.*, **2000**, *38*, 1044–1046.

[119] Hafner, A., Okuda, J., *Organometallics*, **1993**, *12*, 949–950.

**Optimized structures at BLYP/def2-SVP/IEF-PCM(UFF) level**

Ref: [TiCl_4_] - Neat

**
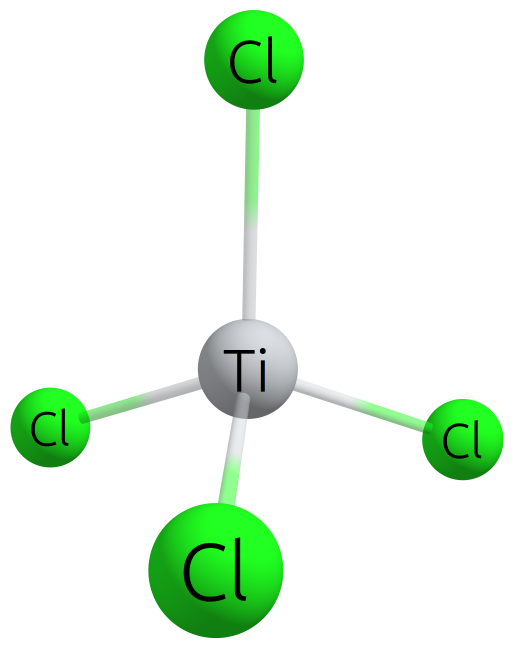
**

5

Ti 0.000000 0.000000 0.000000

Cl 1.269166 1.269166 1.269166

Cl -1.269166 -1.269166 1.269166

Cl 1.269166 -1.269166 -1.269166

Cl -1.269166 1.269166 -1.269166

[Ti(CO)_6_]^-2^ – Acetonitrile (CH_3_CN)


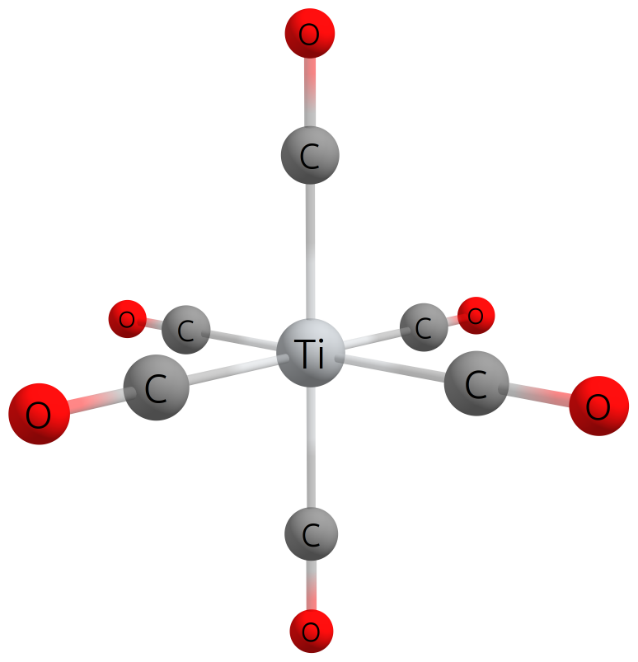


13

Ti 0.000000 0.000003 -0.000002

C -0.034519 -2.051588 0.300429

C 0.034493 2.051593 -0.300432

C -1.558444 0.223401 1.349449

C 1.558458 -0.223569 -1.349406

O -0.053155 -3.228397 0.472506

O -2.452907 0.351987 2.122934

O 2.452919 -0.352313 -2.122868

O 0.053112 3.228404 -0.472499

C 1.369348 0.203780 1.542698

C -1.369329 -0.203613 -1.542741

O 2.155847 0.320765 2.427358

O -2.155822 -0.320458 -2.427425

[Ti(CH_3_)_4_] – CHCl_3_


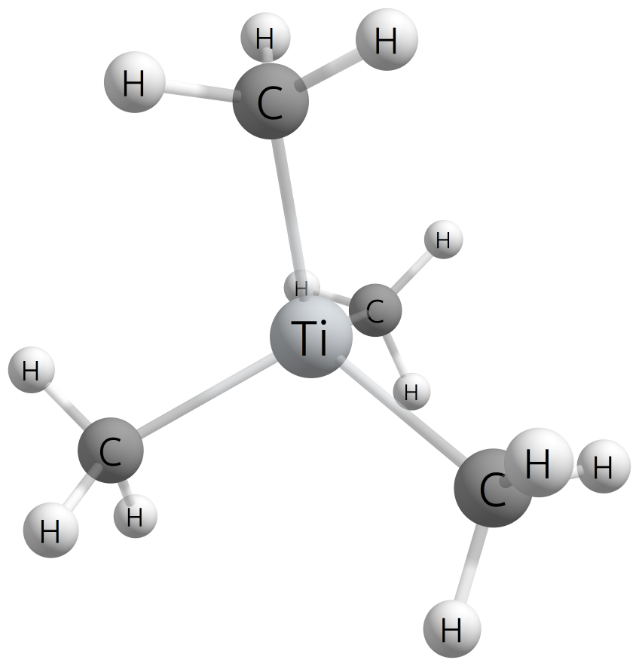


17

Ti 0.00063 -0.01541 0.00455

C -1.74905 -1.02869 0.55415

C 0.34377 1.57950 1.32863

C -0.24586 0.78247 -1.91376

C 1.64872 -1.30393 0.02404

H -2.38035 -0.36338 1.18324

H -1.49701 -1.94720 1.12890

H -2.31550 -1.31109 -0.36044

H 2.22538 -1.16511 0.96528

H 2.30618 -1.07218 -0.84221

H 1.30957 -2.36032 -0.05026

H -0.29999 2.44463 1.06029

H 1.41157 1.88527 1.26231

H 0.11773 1.26606 2.37166

H -1.26786 1.20914 -2.01263

H -0.11007 -0.01086 -2.68128

H 0.50110 1.58792 -2.08329

[Ti(Cp)_2_Cl_2_] – CH_2_Cl_2_


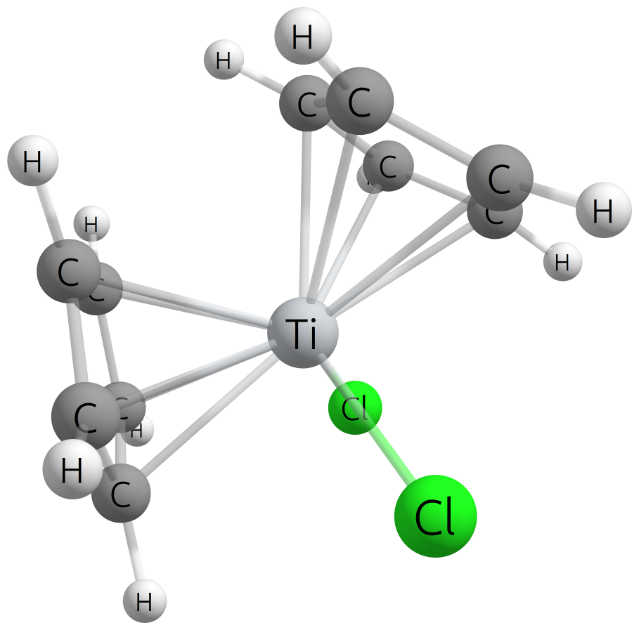


23

H -1.063543 2.740016 0.912787

H 1.063534 2.740044 -0.912840

H 1.251458 2.192220 1.755043

H -1.251212 2.192311 -1.755147

C -1.464383 1.823425 0.469135

C 1.464385 1.823420 -0.469267

C 1.563309 1.536677 0.935045

C -1.563147 1.536734 -0.935209

C -1.997232 0.714146 1.181372

C 1.997087 0.714134 -1.181618

H -2.029132 0.602620 2.269800

H 2.028842 0.602638 -2.270057

C 2.198329 0.266015 1.076336

C -2.198173 0.266093 -1.076625

Ti 0.000001 -0.057886 -0.000069

C -2.443577 -0.255308 0.223886

C 2.443561 -0.255348 -0.224215

H 2.406284 -0.242771 2.021439

H -2.406018 -0.242658 -2.021773

H -2.863060 -1.239369 0.455307

H 2.863005 -1.239406 -0.455717

Cl -0.053056 -1.642361 1.757350

Cl 0.052988 -1.643056 -1.756783

[Ti(Cp)Cl_3_] – CHCl_3_


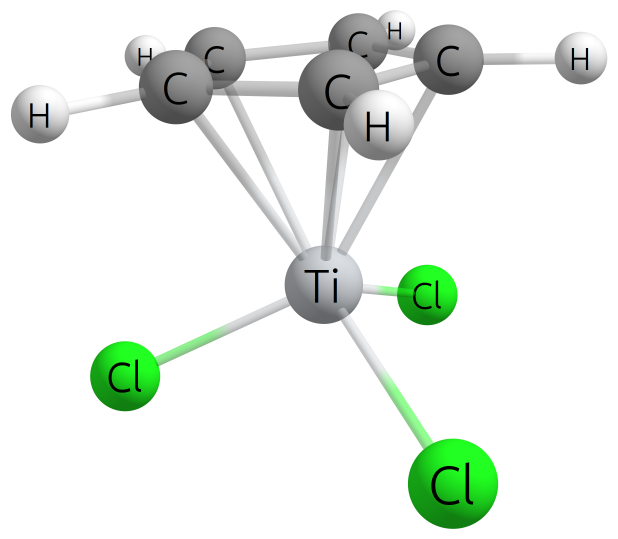


14

Ti 0.227746 0.010589 -0.006373

Cl 1.246022 -0.582265 1.914163

Cl 1.192612 -1.353320 -1.520759

C -1.815582 -0.232680 1.240739

C -1.784788 -1.262085 0.246833

C -1.850286 -0.636393 -1.039590

C -1.903686 0.775987 -0.841345

C -1.880459 1.026039 0.569729

H -1.757966 -0.382824 2.323233

H -1.692821 -2.337999 0.435521

H -1.822498 -1.150501 -2.005535

H -1.915640 1.538334 -1.627829

H -1.872576 2.012268 1.047044

Cl 1.059006 2.056912 -0.457547

[Ti(Cp)_2_Br_2_] – Acetonitrile (CH_3_CN)


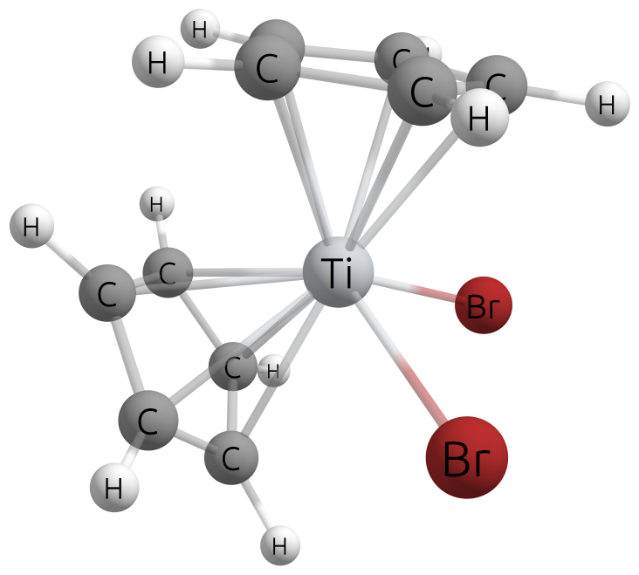


23

C 1.31164 1.10110 1.92776

Br 1.88071 -1.36062 -0.08272

C 0.35128 0.15782 2.42029

C 0.60518 2.23266 1.43402

C -0.94535 0.71984 2.24671

Ti -0.00026 0.37616 0.00035

C -0.79974 1.98833 1.60897

C 0.80142 1.99136 -1.60456

C 0.94503 0.72416 -2.24533

Br -1.88108 -1.36034 0.07953

C -0.60308 2.23726 -1.42866

C -0.35243 0.16432 -2.41977

C -1.31132 1.10781 -1.92474

H 1.61761 2.67217 -1.34228

H -1.05084 3.14206 -1.00679

H 1.89113 0.25167 -2.52170

H -0.57754 -0.82155 -2.83843

H -2.39723 0.97305 -1.91185

H 2.39731 0.96485 1.91526

H 1.05430 3.13780 1.01428

H -1.89217 0.24831 2.52230

H -1.61484 2.67103 1.34817

H 0.57507 -0.82929 2.83669

[TiI_4_] – Benzene (C_6_H_6_)


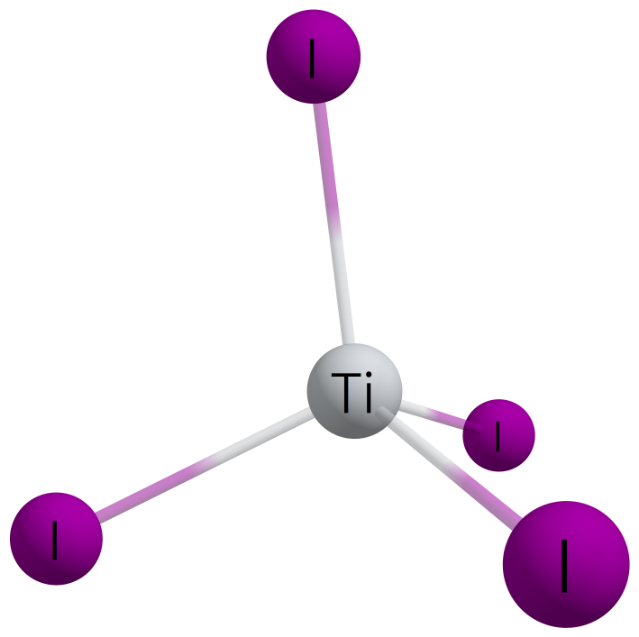


5

Ti -0.00391 0.00047 -0.00236

I 1.36222 -1.73561 1.35316

I 0.12355 2.29970 1.17689

I -2.47320 -0.76201 -0.14808

I 0.98906 0.19772 -2.38099

[Ti(CH_3_)_2_Cl_2_] – CH_2_Cl_2_


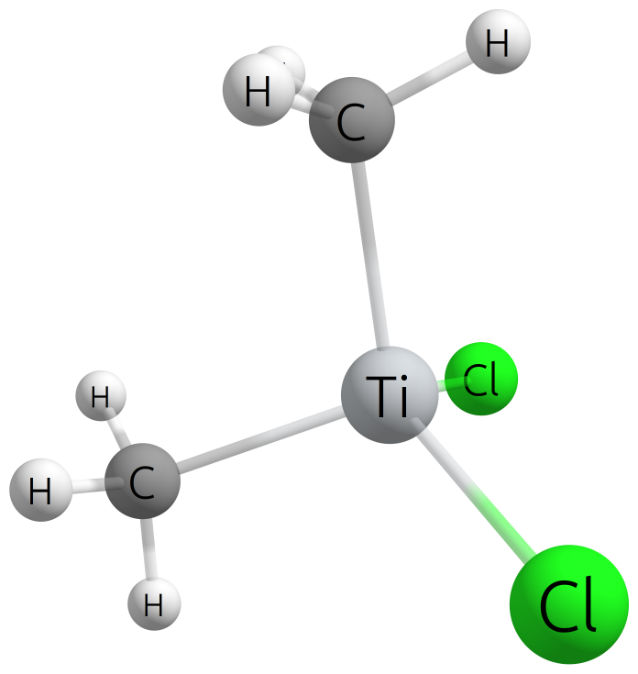


11

Ti 0.000000 0.183714 0.000000

C -0.000000 1.477437 -1.613731

C 0.000000 1.477439 1.613730

Cl 1.935050 -0.940499 0.000000

Cl -1.935050 -0.940500 0.000000

H 0.912402 2.108218 -1.557327

H -0.912400 2.108221 -1.557324

H -0.000003 0.886564 -2.554187

H 0.912401 2.108222 1.557322

H 0.000001 0.886569 2.554187

H -0.912402 2.108221 1.557323

[Ti(CH_3_)_3_Cl] – CH_2_Cl_2_


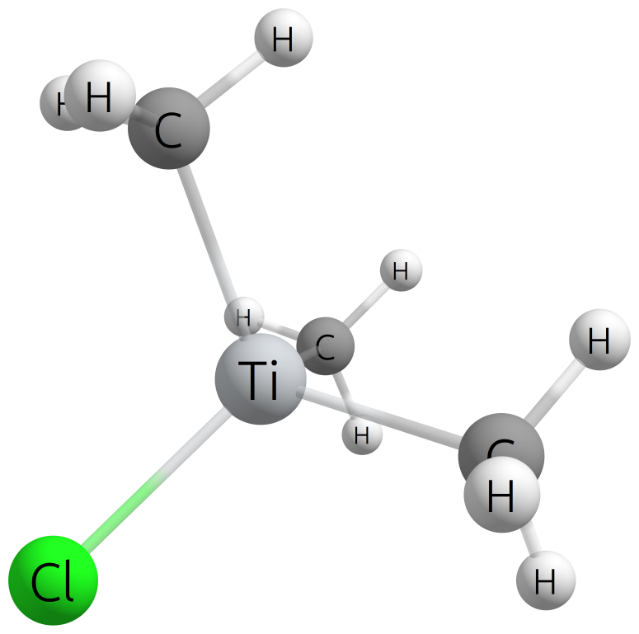


14

Ti -0.241421 -0.002195 -0.002616

Cl 2.007262 -0.004300 0.003305

C -1.008875 1.385588 -1.350841

C -1.025985 -1.863914 -0.513767

C -1.021154 0.490256 1.864146

H -0.675184 1.503863 2.160281

H -0.690275 -0.253451 2.620459

H -2.130592 0.480003 1.787895

H -0.696429 -2.147633 -1.536331

H -2.135037 -1.784028 -0.487082

H -0.688960 -2.631724 0.215264

H -0.679910 1.136363 -2.382585

H -0.661186 2.406393 -1.083222

H -2.118540 1.340028 -1.290555

[Ti(N(CH_2_CH_3_)_3_)Cl] – CHCl_3_


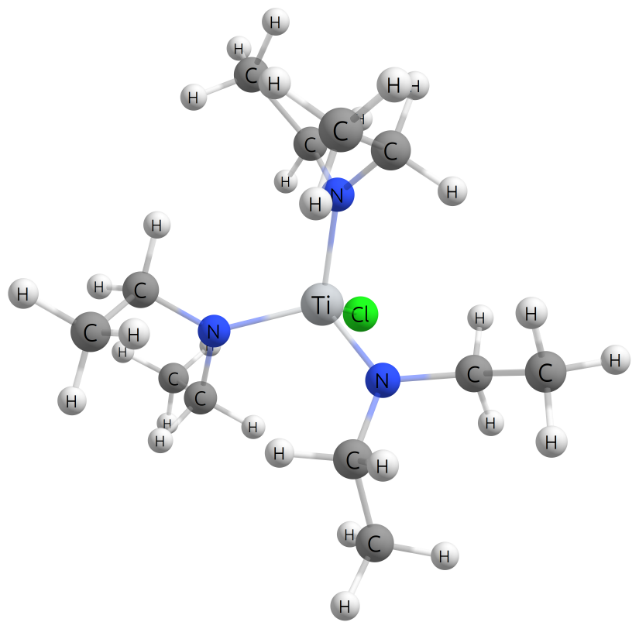


47

H 2.184008 -1.684727 -3.653428

H 0.672429 -0.808008 -3.235377

H -2.837451 0.405719 -2.016038

C 1.320581 -1.663417 -2.956473

H -2.420369 2.992762 -0.723962

H -3.961209 2.085626 -0.831092

H -1.754314 -0.986273 -1.829556

C -3.172300 2.431352 -0.133684

C -2.480718 -0.354898 -1.282396

H 2.383599 -0.582238 -1.403349

H 0.742780 -2.596783 -3.113916

H -4.119485 -1.737800 -1.720290

C 1.812640 -1.526041 -1.497651

H -3.646854 3.135534 0.579684

C -3.681135 -1.222589 -0.840964

N -1.751680 0.307226 -0.187619

C -2.525891 1.259501 0.637424

H 2.529599 -2.350342 -1.275466

H -4.487178 -0.621151 -0.374720

H -0.267256 3.070784 -0.066213

N 0.731616 -1.506267 -0.500042

H 0.368803 2.177798 -2.338480

H -0.148065 -3.358413 -1.097419

H 0.754549 3.923420 -2.177035

H -3.368188 -1.995477 -0.109534

H -1.841551 1.673066 1.411343

C 1.012196 2.908385 -1.807835

Ti -0.005531 -0.056428 0.507813

C 0.791299 2.815394 -0.283033

H -3.317769 0.723301 1.211937

C 0.140299 -2.820308 -0.164696

N 1.100581 1.494918 0.293859

H 1.997088 -3.966638 0.207181

H 1.398965 3.595572 0.229978

C 1.038432 -3.723786 0.708313

H -0.811643 -2.644483 0.382883

H 2.065023 2.699039 -2.082793

H 3.771188 0.938886 -0.561190

H 3.697815 2.680844 -0.141684

C 3.659559 1.655290 0.277119

H 0.524026 -4.682535 0.926110

C 2.356864 1.407770 1.068400

H 1.262899 -3.222347 1.670533

H 2.406478 0.399498 1.535966

H 2.314522 2.124847 1.924668

H 4.535651 1.538651 0.947974

Cl -0.158882 -0.632809 2.762405

[Ti(N(CH_2_CH_3_)_2_)_4_] – CH_2_Cl_2_


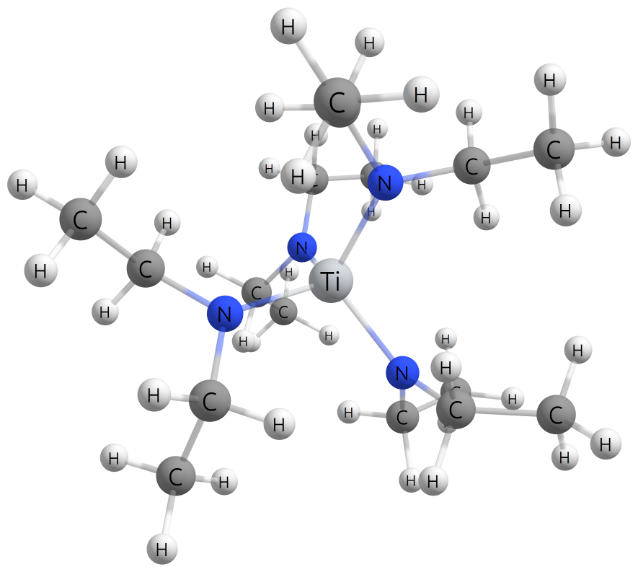


61

H -1.071965 3.445507 -3.228847

H 3.019681 3.198213 0.495361

H 3.356531 1.559339 -0.147105

C 3.220257 2.147708 0.782166

H 4.177552 2.123690 1.345407

C -1.567383 2.701782 -2.569912

H 0.340853 1.837973 -2.019342

H -0.455840 1.023133 -3.384056

C -0.646651 1.473099 -2.376686

H 0.459685 3.995032 -0.336013

H -1.793139 3.195112 -1.603181

H -2.528716 2.426999 -3.047612

H 1.193287 4.359773 1.259558

C 0.273380 3.999894 0.757027

C 2.076227 1.548783 1.633246

N 1.574333 -1.126344 -0.692718

N 0.760515 1.483848 0.977173

H -3.854033 1.306749 -1.582766

H -0.527584 4.739918 0.966372

H 1.986865 2.124258 2.586012

H 2.379947 0.522458 1.936923

N -1.110787 0.431968 -1.446512

Ti 0.084032 -0.071450 0.015842

C -0.153981 2.601485 1.263392

H -2.403994 -0.305300 -2.959760

C -3.656474 0.265682 -1.262014

H -1.141653 2.355265 0.818740

C -2.333154 -0.284171 -1.845324

H -3.627685 0.251768 -0.153949

H -0.326303 2.671308 2.366982

H -4.513568 -0.360744 -1.589266

H -2.243198 -1.348036 -1.536414

H -0.113820 -0.211627 3.088813

N -0.818916 -1.173358 1.347752

H -0.857170 -2.689448 -0.108226

H -0.544370 -3.276387 1.537422

C -1.163051 -2.550269 0.951882

C -0.950984 -0.874485 2.784345

H -0.828386 -1.815831 3.372687

H -2.398566 0.777051 2.672054

C -2.273924 -0.188938 3.201417

C -2.646707 -2.964118 1.092662

H -2.274366 0.018725 4.292928

H -2.792414 -3.992289 0.699421

H -3.315043 -2.281614 0.530483

H -2.973660 -2.966923 2.151413

H -3.158298 -0.815408 2.973849

C 1.931959 -1.162719 -2.121124

C 2.288880 -2.102260 0.152336

C 1.352710 -2.360479 -2.911320

H 3.040857 -1.158164 -2.242761

H 1.581425 -0.224371 -2.597381

H 1.688151 -3.333226 -2.497073

H 1.679086 -2.321435 -3.971990

H 0.243867 -2.345028 -2.892086

C 3.806416 -1.852255 0.321699

H 2.148309 -3.140101 -0.237295

H 1.816867 -2.094194 1.156638

H 4.351227 -1.914653 -0.641981

H 4.244624 -2.617357 0.996096

H 4.005107 -0.853457 0.758814

[TiF_6_]^-2^ – H_2_O


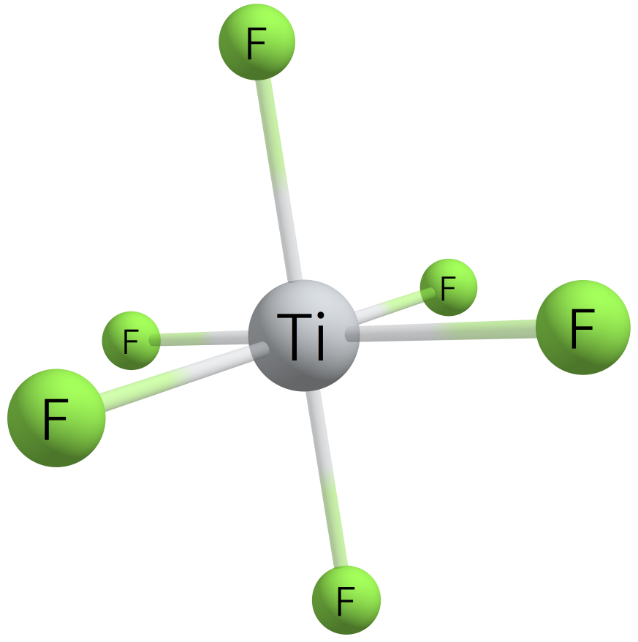


7

Ti 0.000000 0.000001 0.000000

F 1.330555 0.338693 1.291219

F -1.330573 -0.338711 -1.291198

F 0.971046 1.009227 -1.261129

F -0.971043 -1.009228 1.261129

F -0.919223 1.556386 0.536768

F 0.919237 -1.556370 -0.536790

[Ti(Cp)_2_FCl] – CH_2_Cl_2_


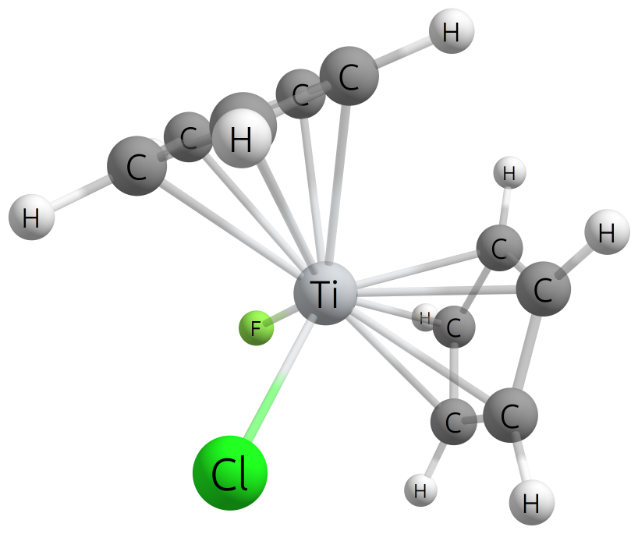


23

H 1.118434 -2.786285 -0.321361

H -1.045280 -1.848869 -2.024328

H -1.326696 -2.717876 0.545817

H 1.237202 -1.049344 -2.427296

C 1.501311 -1.760897 -0.291798

C -1.460537 -1.271808 -1.192481

C -1.611621 -1.732296 0.161755

C 1.565394 -0.848872 -1.402305

C 2.069171 -1.110860 0.842708

C -1.952207 0.058330 -1.258820

H 2.123784 -1.522678 1.856288

H -1.941003 0.709430 -2.139404

C -2.259412 -0.698821 0.904510

C 2.160421 0.357148 -0.939030

Ti 0.002225 0.074030 0.225397

C 2.455865 0.204493 0.452540

C -2.442589 0.415011 0.045609

H -2.479177 -0.725355 1.976873

H 2.324755 1.260779 -1.532948

H 2.866271 0.978493 1.110162

H -2.850073 1.390270 0.329681

Cl -0.017852 2.395614 -0.270447

F 0.007950 0.254307 2.036584

[Ti(Cp)_2_ICl] – CH_2_Cl_2_


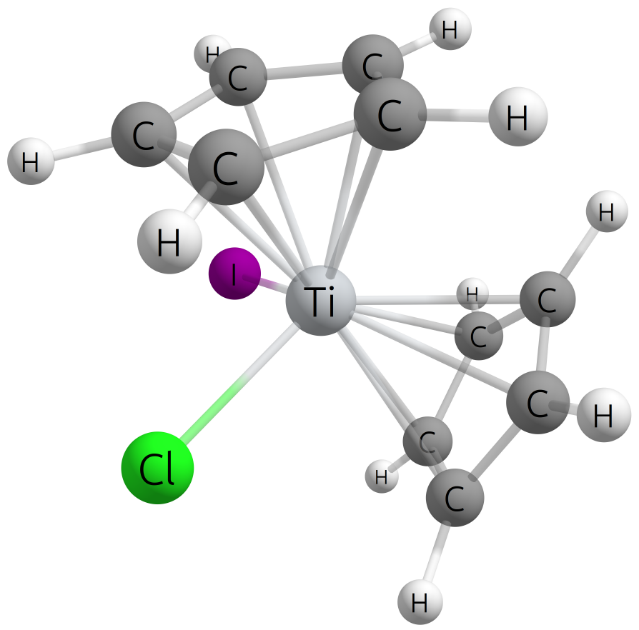


23

H -1.952687 1.129306 -2.546036

H -3.344024 -0.969185 -1.190039

H -1.123550 -1.220796 -2.755952

H -3.446241 1.330020 -0.270069

C -1.607021 1.509188 -1.579829

C -2.384309 -1.396936 -0.884196

C -1.217675 -1.529787 -1.709705

C -2.393452 1.609930 -0.380814

C -0.306174 2.004306 -1.293397

C -2.082674 -1.945810 0.394760

H 0.534362 2.047859 -1.992787

H -2.751389 -1.963476 1.261344

C -0.213184 -2.192664 -0.941301

C -1.580660 2.217790 0.623655

Ti -0.665221 0.004278 0.074181

C -0.286358 2.434857 0.076665

C -0.735925 -2.430017 0.363123

H 0.786162 -2.461328 -1.292415

H -1.885461 2.424299 1.653171

H 0.576213 2.848151 0.608605

H -0.194551 -2.876517 1.202964

I 2.186159 -0.025015 -0.104396

Cl -0.681519 -0.043636 2.424025

[Ti(N(CH_3_)_2_)_4_] – CH_2_Cl_2_


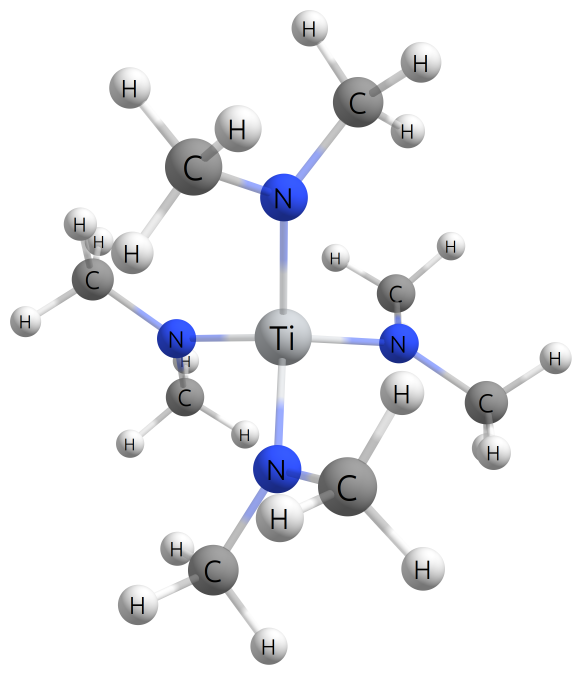


37

H 0.305583000 1.929930000 -1.897833000

H -0.571260000 1.221036000 -3.297615000

C -0.669174000 1.511177000 -2.221610000

C 2.194936000 1.610756000 1.408311000

N 1.623053000 -1.205732000 -0.673579000

N 0.823086000 1.372103000 0.977642000

H 2.261524000 1.805188000 2.508252000

H 2.836891000 0.738671000 1.178147000

N -1.053349000 0.381590000 -1.378888000

Ti 0.130553000 -0.190537000 0.054097000

C -0.060392000 2.501728000 1.257441000

H -2.402663000 -0.507488000 -2.788776000

H -1.083208000 2.306791000 0.875241000

C -2.361247000 -0.159154000 -1.726138000

H -0.144036000 2.708885000 2.353449000

H -2.619411000 -1.020363000 -1.079721000

H -1.008826000 0.107501000 2.914766000

N -0.842562000 -1.287092000 1.326389000

H -0.784986000 -2.913937000 -0.043963000

H -0.732697000 -3.402112000 1.682869000

C -1.171627000 -2.664164000 0.965623000

C -1.282322000 -0.937101000 2.670818000

H -0.822513000 -1.598493000 3.448189000

C 2.095545000 -1.191835000 -2.052593000

C 2.380689000 -2.128235000 0.168749000

H 3.168487000 -0.882179000 -2.125192000

H 1.500302000 -0.491454000 -2.670180000

H 2.352727000 -3.176097000 -0.222988000

H 1.973006000 -2.145088000 1.199401000

H 2.028921000 -2.201845000 -2.531528000

H 3.460633000 -1.843783000 0.237054000

H -2.275371000 -2.845840000 0.956053000

H -3.175594000 0.601405000 -1.614895000

H -2.390447000 -1.038594000 2.789782000

H -1.419792000 2.340269000 -2.181732000

H 0.302095000 3.447409000 0.781298000

H 2.644361000 2.504813000 0.905694000

[Ti(N(CH_2_CH_2_CH_3_)_2_)_4_] – CH_2_Cl_2_


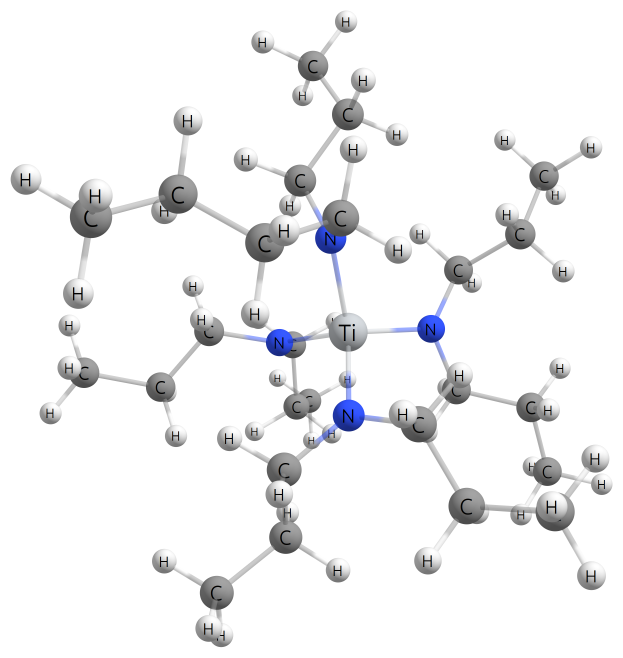


88

H -3.563605 2.629689 -0.928880

H -1.877389 3.154653 -1.106194

C -2.713094 3.080479 -0.378269

C -1.794248 -1.989437 -3.055674

H -1.228952 0.015140 -2.460309

H 0.026173 -0.850816 -3.377490

C -0.730490 -0.974659 -2.560615

H -3.858279 -0.035608 -1.489452

H -2.548181 -2.149293 -2.256193

H -1.314389 -2.974458 -3.234255

H -4.720100 0.879224 -0.232238

C -4.208694 -0.084660 -0.436327

C -2.275987 2.134870 0.772896

N 1.063776 1.615206 -0.529235

N -1.908700 0.765694 0.383593

H -0.850141 -3.977622 -1.240796

H -3.092011 2.097462 1.535520

H -1.412454 2.611401 1.288906

N -0.050961 -1.272557 -1.289611

Ti -0.086402 0.139387 0.060365

C -2.981624 -0.235713 0.501472

H 1.112526 -2.725756 -2.310202

C 0.083660 -3.759900 -0.683149

H -2.538399 -1.239044 0.321941

C 0.764417 -2.496822 -1.273552

H -0.224655 -3.535543 0.360185

H -3.360882 -0.255243 1.555222

H 1.693117 -2.312905 -0.688603

H -0.926233 0.337512 3.023540

N 0.576701 -0.485885 1.787048

H 2.477810 -0.528693 0.889035

H 2.486000 0.041589 2.570970

C 2.026443 -0.718676 1.888614

C -0.152449 -0.459398 3.065612

H 0.545786 -0.166447 3.887691

H -1.562590 -2.061746 2.649722

C -0.863569 -1.779940 3.465138

C 2.484549 -2.120599 2.368334

H 2.012681 -2.896223 1.729192

H 2.112397 -2.298207 3.398949

H -0.120866 -2.601498 3.532121

C 1.499984 1.804676 -1.926586

C 1.662699 2.536511 0.455990

C 2.702624 0.947127 -2.408300

H 1.735765 2.878361 -2.110362

H 0.647811 1.575797 -2.601816

H 2.794669 1.102744 -3.508109

H 2.444369 -0.124502 -2.269308

C 1.228862 4.022791 0.350538

H 2.777266 2.503223 0.406202

H 1.397070 2.173603 1.472130

H 1.483668 4.419640 -0.655041

H 0.124218 4.085839 0.435401

C -1.631927 -1.661900 4.795404

H -2.145533 -2.610191 5.054088

H -0.952001 -1.411055 5.637089

H -2.405209 -0.865993 4.747959

C 1.002025 -4.997026 -0.704212

H 0.498852 -5.883681 -0.267541

H 1.304645 -5.261708 -1.739525

H 1.932546 -4.823204 -0.123445

C -2.488772 -1.521368 -4.349413

H -1.756695 -1.369831 -5.170705

H -3.235023 -2.262437 -4.701063

H -3.019678 -0.557719 -4.198517

C 4.017551 -2.275662 2.346046

H 4.510772 -1.520143 2.993568

H 4.330074 -3.277119 2.705469

H 4.423146 -2.146490 1.320365

C 1.889956 4.904601 1.427374

H 2.997385 4.883843 1.345975

H 1.569589 5.962775 1.339875

H 1.628615 4.560092 2.450477

C -3.103886 4.485295 0.119673

H -3.400678 5.146552 -0.719788

H -3.958088 4.441636 0.828255

H -2.262128 4.978935 0.649872

C 4.071646 1.233081 -1.755141

H 4.301646 2.318753 -1.838672

H 4.020112 1.013357 -0.667663

C 5.220864 0.416645 -2.379189

H 5.337095 0.642568 -3.460385

H 6.191206 0.631949 -1.886228

H 5.034991 -0.674594 -2.289679

C -5.216886 -1.237947 -0.267485

H -5.592127 -1.297347 0.776104

H -6.096325 -1.109872 -0.930775

H -4.756171 -2.219263 -0.508267

[Ti(OCH(CH_3_)_2_)_4_] – CH_2_Cl_2_


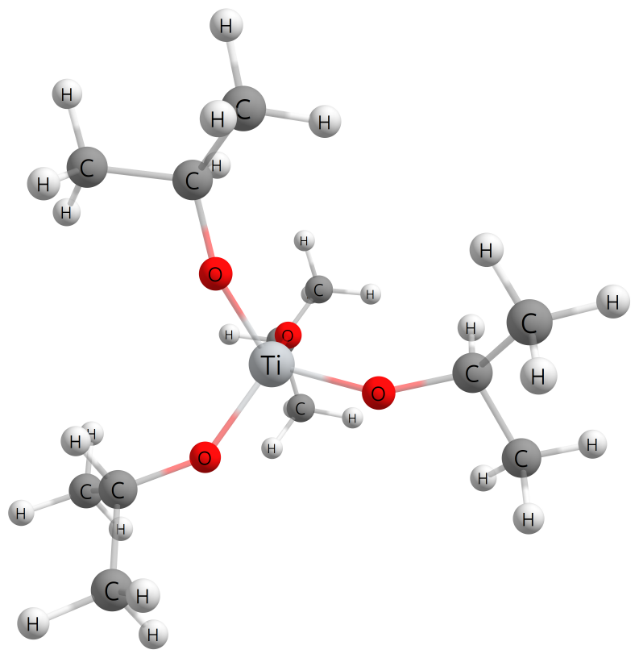


45

Ti 0.622235000 0.011427000 -0.360925000

O -1.098699000 0.023246000 0.257567000

O 1.717625000 0.614948000 0.944281000

O 1.019759000 -1.698169000 -0.849063000

O 0.815174000 1.084514000 -1.811424000

C -2.005258000 0.685596000 1.139049000

H -1.434861000 1.434955000 1.739865000

C 1.578303000 -2.483246000 -1.906623000

H 1.649475000 -1.839807000 -2.816182000

C 2.790296000 0.520413000 1.883567000

H 3.734395000 0.385504000 1.304640000

C 1.234478000 2.327192000 -2.375686000

H 1.808709000 2.882486000 -1.595628000

C -3.075083000 1.430047000 0.318216000

H -3.659788000 0.713029000 -0.294515000

H -3.776253000 1.975934000 0.982249000

H -2.602202000 2.163009000 -0.365556000

C -2.615437000 -0.346771000 2.106007000

H -3.191668000 -1.110286000 1.543394000

H -1.818202000 -0.864724000 2.675497000

H -3.298283000 0.143755000 2.829795000

C 2.887128000 1.837682000 2.673900000

H 1.963962000 2.004092000 3.266725000

H 3.749671000 1.814565000 3.370964000

H 3.018783000 2.697061000 1.986702000

C 2.591978000 -0.707993000 2.789912000

H 1.662385000 -0.604333000 3.387014000

H 2.514382000 -1.631014000 2.181860000

H 3.445228000 -0.822294000 3.489196000

C 2.996939000 -2.942154000 -1.522089000

H 2.968391000 -3.576707000 -0.612196000

H 3.456329000 -3.530172000 -2.342924000

H 3.648117000 -2.068858000 -1.316820000

C 0.635361000 -3.660687000 -2.213112000

H 0.536793000 -4.320953000 -1.326514000

H -0.373610000 -3.291037000 -2.484270000

H 1.022654000 -4.265589000 -3.058477000

C 0.000387000 3.161995000 -2.763757000

H 0.302789000 4.147589000 -3.173277000

H -0.599350000 2.632762000 -3.532799000

H -0.645413000 3.338456000 -1.880570000

C 2.168404000 2.058651000 -3.569929000

H 1.631131000 1.501953000 -4.365450000

H 2.539458000 3.011474000 -3.999697000

H 3.043266000 1.456785000 -3.253016000

[Ti(CH_3_)Cl_3_] – CH_2_Cl_2_


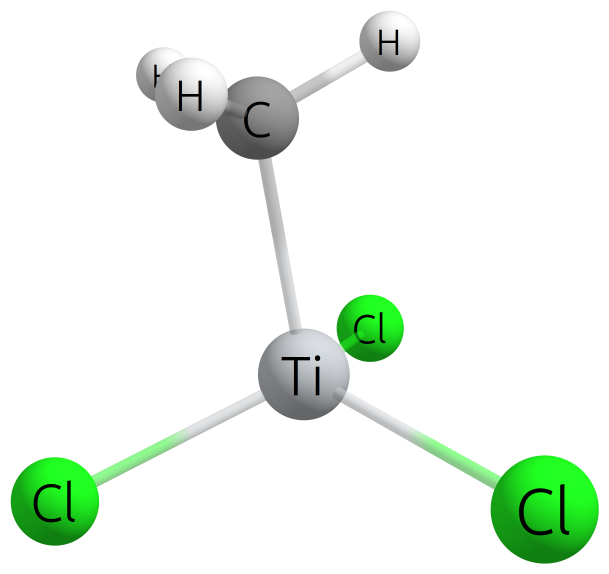


8

Ti 0.000010 -0.006652 0.083168

C -0.000111 0.035909 2.143957

Cl -1.871658 -1.080124 -0.426949

Cl 1.872919 -1.078010 -0.426879

H -0.913416 0.572504 2.475106

H 0.912486 0.573644 2.475207

H 0.000515 -1.008780 2.517867

Cl -0.001210 2.145988 -0.449797

[Ti(CH_3_)Br_3_] – CH_2_Cl_2_


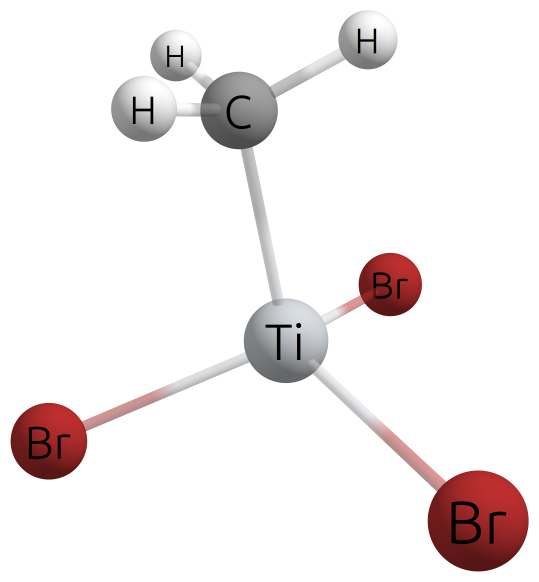


8

Ti -0.000399000 0.248171000 0.086692000

C 0.002896000 1.462577000 -1.574493000

H 0.893892000 2.122433000 -1.525097000

H -0.934432000 2.056447000 -1.576594000

H 0.049913000 0.803227000 -2.466875000

Br 1.981567000 -1.050679000 -0.139732000

Br -2.008784000 -1.017333000 -0.098957000

Br 0.015346000 1.757638000 1.931832000

[TiBr_4_] – CH_2_Cl_2_


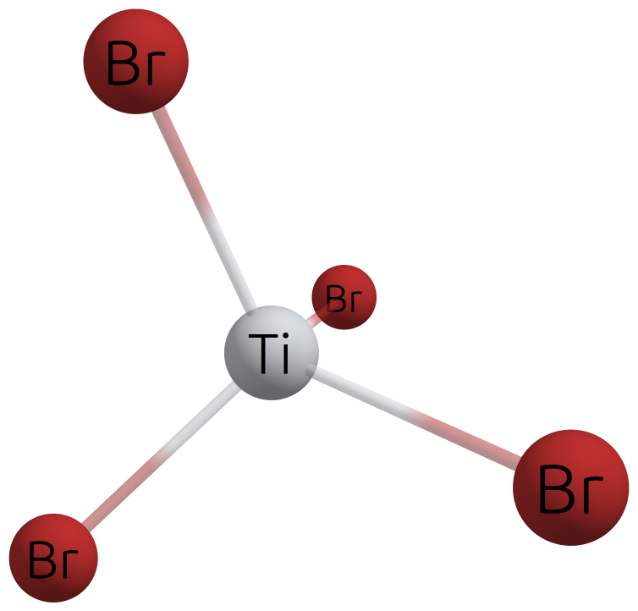


5

Ti 0.017197000 -0.010554000 -0.014970000

Br -2.248306000 -0.680093000 -0.137775000

Br 1.237839000 -1.577211000 1.266129000

Br 0.885063000 0.191955000 -2.199174000

Br 0.104640000 2.079788000 1.079845000

[Ti(Cp)_2_I_2_] – CH_2_Cl_2_


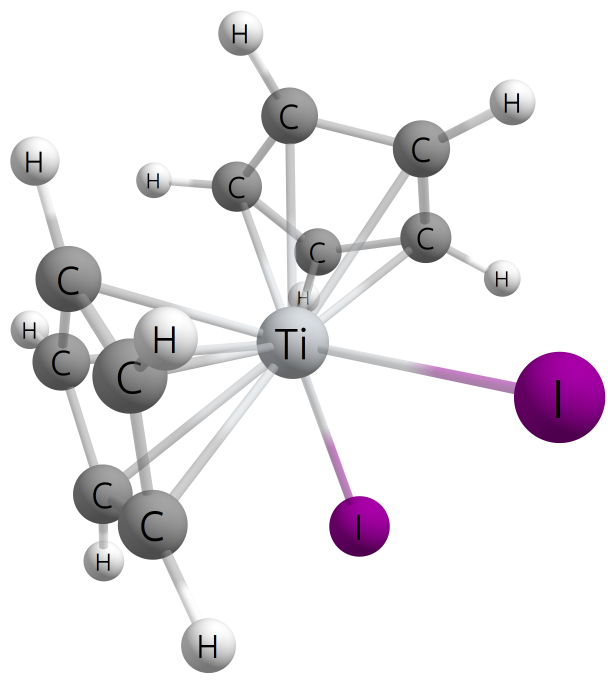


23

H 1.115237 3.448792 1.084527

H -1.115004 3.449499 -1.083469

H 1.586080 3.105935 -1.260475

H -1.585904 3.105359 1.260966

C 0.602079 2.561791 1.467841

C -0.601975 2.562535 -1.467042

C 0.820535 2.383053 -1.559155

C -0.820463 2.382400 1.559708

C 1.225867 1.400862 2.005294

C -1.225942 1.401771 -2.004607

H 2.303370 1.224914 2.074557

H -2.303466 1.225853 -2.073601

C 1.062458 1.121047 -2.179011

C -1.062554 1.120296 2.179264

Ti 0.000146 0.742708 -0.000046

C 0.196528 0.498737 2.428420

C -0.196726 0.499638 -2.428129

H 2.044390 0.700549 -2.410577

H -2.044547 0.699766 2.410505

H 0.350267 -0.493429 2.863952

H -0.350633 -0.492451 -2.863772

I -2.062968 -1.206655 0.012695

I 2.062933 -1.206687 -0.013018

[Ti(Cp)_2_BrI] – CH_2_Cl_2_


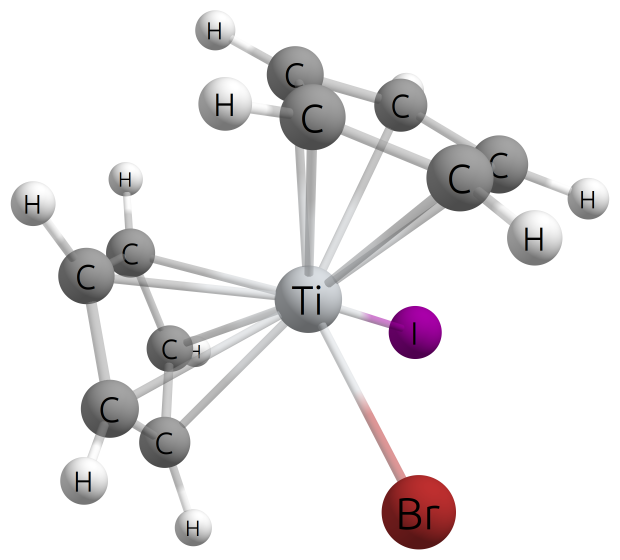


23

H -1.298285 -3.217261 -0.889092

H -2.918099 -2.025093 1.093143

H -0.426181 -3.077009 1.454952

H -3.191550 -1.287807 -1.272414

C -1.136819 -2.237173 -1.348281

C -2.046335 -1.496525 1.490641

C -0.736081 -2.051390 1.680777

C -2.133329 -1.221072 -1.545841

C 0.093482 -1.752569 -1.870368

C -2.019393 -0.144493 1.933859

H 1.050653 -2.282823 -1.857571

H -2.855094 0.561332 1.894785

C 0.081199 -1.044877 2.280161

C -1.516033 -0.133480 -2.235477

Ti -0.539608 -0.323363 0.009667

C -0.138776 -0.443735 -2.412022

C -0.699187 0.139181 2.413376

H 1.126607 -1.162360 2.576719

H -2.010751 0.791705 -2.542218

H 0.613505 0.204414 -2.872031

H -0.348348 1.101917 2.797315

I 2.281934 0.075044 0.031428

Br -1.065884 2.167041 -0.130940

[Ti(Cp)_2_(N_3_)_2_] – CHCl_3_


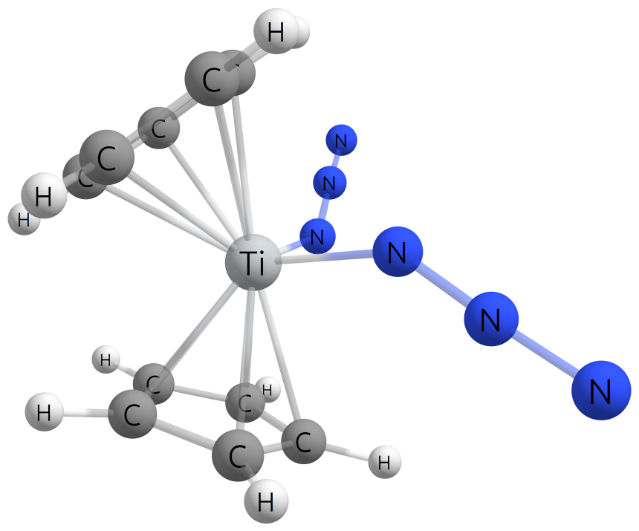


27

H 1.364844 -2.966289 -0.098653

H -1.377828 -2.960808 0.084461

H 0.265552 -2.369877 2.181961

H -0.274177 -2.355615 -2.194015

C 1.378699 -2.038454 -0.679199

C -1.386790 -2.036184 0.670220

C -0.520052 -1.725168 1.772909

C 0.514029 -1.716927 -1.780534

C 2.261077 -0.941832 -0.479140

C -2.263941 -0.934174 0.476938

H 3.031949 -0.868123 0.296406

H -3.034564 -0.852203 -0.298027

C -0.907111 -0.450725 2.284943

C 0.907751 -0.441513 -2.285164

Ti 0.000158 -0.151178 0.000607

C 1.963604 0.052859 -1.472202

C -1.961128 0.053391 1.475442

H -0.427718 0.078311 3.113866

H 0.431392 0.094597 -3.111287

H 2.458771 1.024573 -1.578180

H -2.451250 1.027025 1.587060

N -0.998259 1.201574 -1.097879

N 1.003035 1.193428 1.105191

N -2.064856 1.776388 -1.148055

N 2.069117 1.769523 1.152408

N -3.072762 2.357531 -1.248167

N 3.076969 2.351083 1.250469

[Ti(Cp)_2_(NCS)_2_] – CH_2_Cl_2_


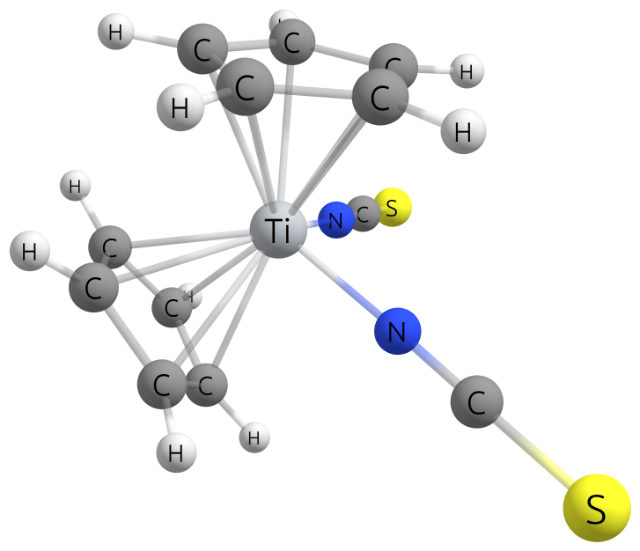


27

H -0.979285 -3.373294 1.062607

H 0.972783 -3.378033 -1.057258

H -1.691001 -2.858037 -1.337496

H 1.687077 -2.862045 1.336486

C -0.536763 -2.456618 1.464816

C 0.534399 -2.460204 -1.461301

C -0.868152 -2.188604 -1.609888

C 0.867104 -2.189481 1.609765

C -1.251918 -1.327646 1.955682

C 1.254510 -1.334027 -1.951322

H -2.340575 -1.208068 1.960502

H 2.343547 -1.217769 -1.953160

C -1.001421 -0.906922 -2.223629

C 1.006125 -0.907513 2.221358

Ti -0.000041 -0.601682 0.000038

C -0.296191 -0.363699 2.417219

C 0.303053 -0.367648 -2.416807

H -1.945006 -0.409295 -2.467616

H 1.951976 -0.412552 2.461976

H -0.525486 0.626032 2.825713

H 0.536517 0.620910 -2.825757

N -1.468979 0.767712 -0.028335

N 1.467646 0.769309 0.026554

C 2.363798 1.574431 0.023436

C -2.365870 1.572055 -0.024982

S 3.558860 2.660778 0.023963

S -3.562131 2.657052 -0.025240

[Ti(Cp)Br_3_] – CHCl_3_


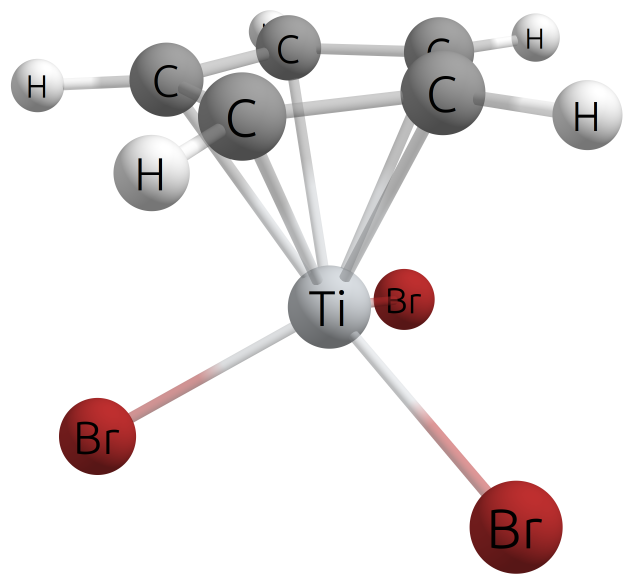


14

Ti -0.018695 -0.002568 0.217626

C 0.231887 1.117548 2.333743

C 1.043652 -0.060919 2.363719

C 0.175436 -1.197571 2.298378

C -1.167475 -0.722028 2.208843

C -1.133035 0.710662 2.229241

H 0.595175 2.149653 2.361629

H 2.138664 -0.088353 2.403119

H 0.488178 -2.246516 2.293820

H -2.064895 -1.342465 2.112013

H -1.999221 1.376428 2.148624

Br 1.178553 -1.867847 -0.756447

Br 1.141248 1.908643 -0.715094

Br -2.138355 -0.008750 -0.948760

[Ti(Cp)I_3_] – CH_2_Cl_2_


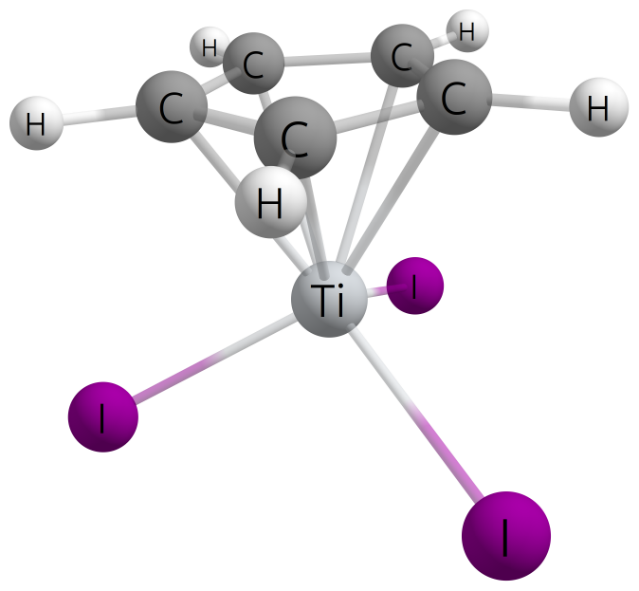


14

Ti -0.011845 -0.007348 0.505220

C 0.271639 1.145659 2.596646

C 1.168300 0.030730 2.588255

C 0.384723 -1.168062 2.579609

C -0.992565 -0.793586 2.560636

C -1.063240 0.638004 2.566799

H 0.557981 2.200950 2.615057

H 2.262805 0.084841 2.576576

H 0.772837 -2.191228 2.581689

H -1.845449 -1.479544 2.530586

H -1.979647 1.237514 2.538550

I 1.471806 -1.894278 -0.634882

I 0.944586 2.210411 -0.611245

I -2.380941 -0.293630 -0.665364

[Ti(Me-Cp)Cl_3_] – CH_2_Cl_2_


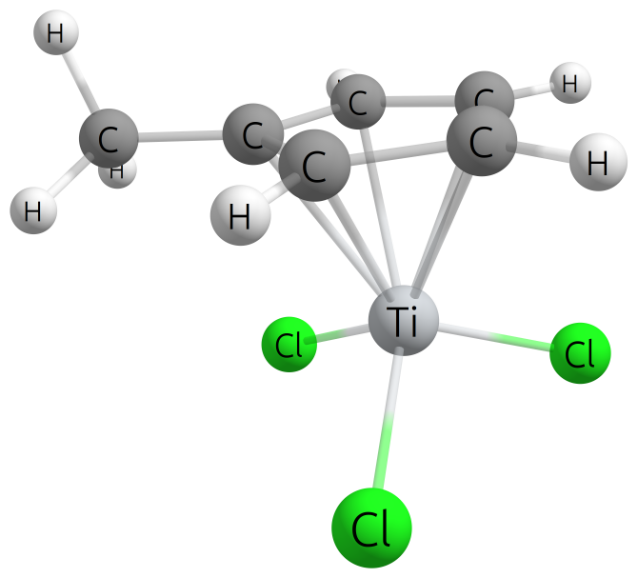


17

Ti -0.414888 0.075635 0.006106

C 0.528704 -1.963444 -0.796521

C 0.507771 -2.020971 0.632804

C 1.414624 -1.028323 1.124046

C 2.002273 -0.347770 0.008969

C 1.431976 -0.926072 -1.179067

H -0.070583 -2.577847 -1.475723

H -0.119625 -2.680679 1.243505

H 1.606687 -0.803380 2.178767

H 1.641110 -0.603244 -2.205442

C 3.048628 0.733934 0.068924

H 4.060014 0.286166 -0.036831

H 2.918973 1.468389 -0.748236

H 3.020532 1.271815 1.035783

Cl -1.050520 0.732809 2.067477

Cl 0.006265 1.993603 -1.113059

Cl -2.340064 -0.650489 -0.912129

[Ti(Me-Cp)Br_3_] – CH_2_Cl_2_


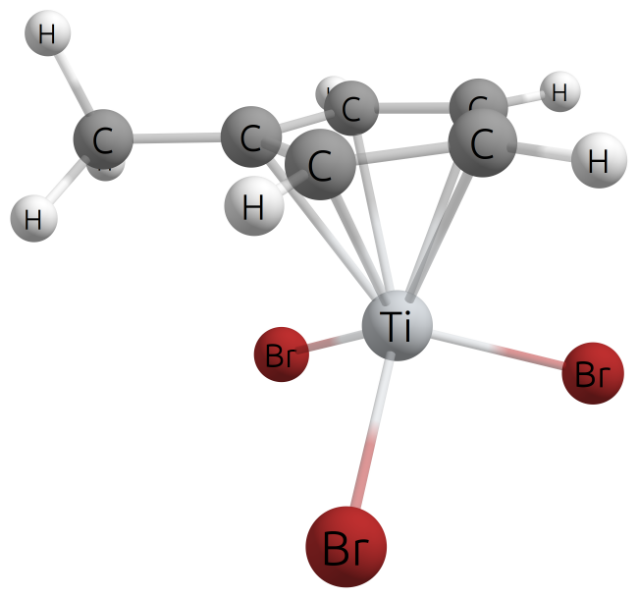


17

Ti -0.056277 -0.008514 0.166948

C 0.290241 -0.779451 2.400312

C 0.214998 0.649327 2.431226

C 1.349044 1.162863 1.726507

C 2.137255 0.060484 1.255325

C 1.460990 -1.140408 1.667040

H -0.434074 -1.473334 2.838128

H -0.585845 1.244191 2.885315

H 1.580045 2.221131 1.566720

H 1.787660 -2.161642 1.441954

C 3.448679 0.149059 0.519105

H 4.284138 0.210380 1.249350

H 3.620978 -0.741298 -0.114240

H 3.499171 1.052418 -0.118614

Br -0.400858 2.155194 -0.874058

Br 0.846937 -1.362815 -1.630021

Br -2.329542 -0.814545 0.406406

[Ti(Me-Cp)I_3_] – CH_2_Cl_2_


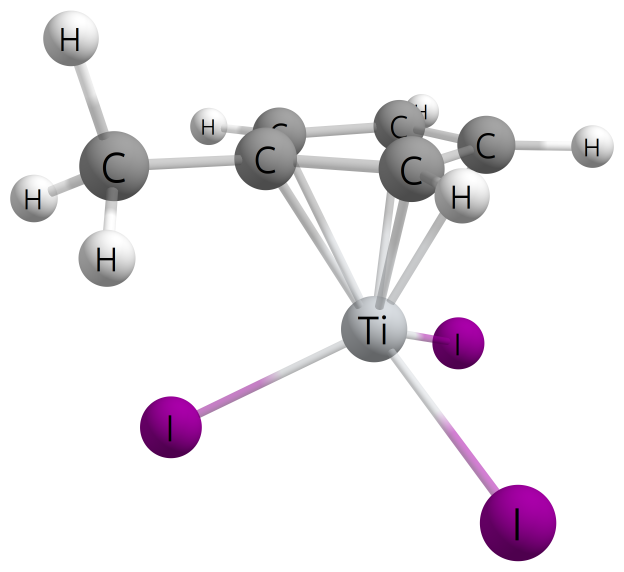


17

Ti 0.043061 0.008282 0.419146

C -0.177564 -0.681813 2.689473

C -0.319997 0.740630 2.643094

C 0.939253 1.294469 2.247234

C 1.872605 0.224051 2.050310

C 1.161078 -1.000090 2.303874

H -0.955674 -1.399576 2.963450

H -1.235241 1.306237 2.852374

H 1.164328 2.359227 2.129829

H 1.582319 -2.008486 2.223868

I -0.394386 2.348081 -0.773636

I -2.286756 -1.220391 0.022545

I 1.669945 -1.265980 -1.258789

C 3.340904 0.367139 1.745870

H 3.898547 0.518408 2.695446

H 3.749601 -0.537482 1.258402

H 3.544544 1.242527 1.099918

[Ti(Me_4_-Cp)Cl_3_] – CH_2_Cl_2_


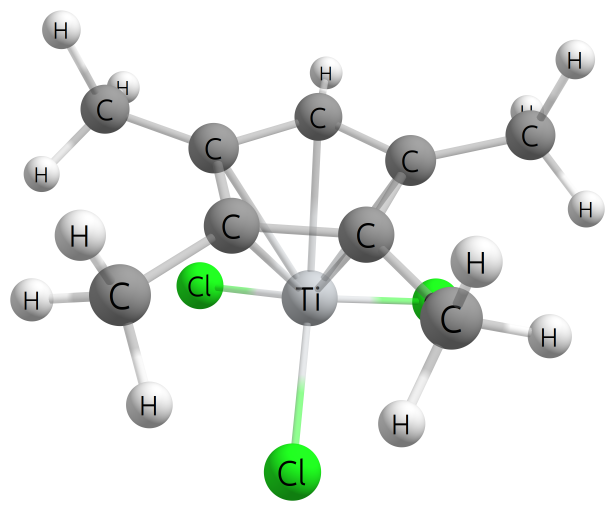


26

Ti -0.681265 0.001748 0.178135

C 0.968090 1.169540 -1.151033

C 0.531611 0.000196 -1.857553

C 0.962288 -1.172245 -1.152646

C 1.642830 -0.727226 0.036864

C 1.646568 0.719570 0.037774

H -0.037576 0.002251 -2.795733

C 2.320979 -1.610065 1.052863

H 3.402719 -1.706384 0.814794

H 2.241399 -1.196641 2.075682

H 1.893761 -2.630561 1.062390

Cl -2.046740 -1.754223 -0.244920

Cl -0.464100 0.001412 2.438655

Cl -2.041272 1.761494 -0.247170

C 0.829272 -2.592929 -1.637365

H 0.007589 -2.689761 -2.370966

H 1.770805 -2.909239 -2.136222

H 0.635154 -3.303063 -0.811559

C 2.329516 1.597654 1.054670

H 2.247256 1.183952 2.077167

H 3.411865 1.687778 0.816946

H 1.908321 2.620638 1.064812

C 0.841621 2.591530 -1.633790

H 1.784309 2.903931 -2.132938

H 0.019887 2.693264 -2.366658

H 0.651578 3.301602 -0.806976

[Ti(Me_4_-Cp)Br_3_] – CH_2_Cl_2_


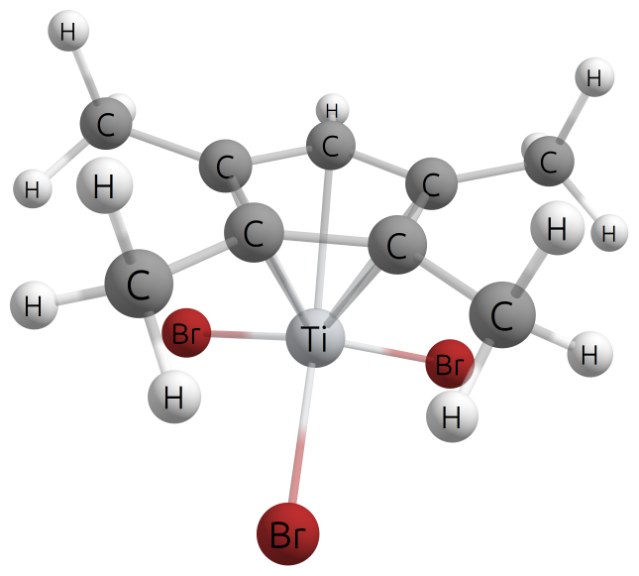


26

Ti -0.397764000 6.292435000 5.589117000

C -1.605898000 8.164881000 4.648660000

C -0.341607000 8.619856000 5.151704000

C -0.327414000 8.504370000 6.581189000

C -1.583750000 7.914924000 6.973291000

C -2.376987000 7.712659000 5.780983000

H 0.478122000 9.015602000 4.538638000

C -2.033573000 7.677805000 8.392310000

H -2.505535000 8.599138000 8.799281000

H -2.779931000 6.865263000 8.460974000

H -1.186217000 7.422751000 9.056841000

C 0.728243000 9.056255000 7.503924000

H 1.702198000 9.157709000 6.990377000

H 0.421297000 10.067426000 7.849767000

H 0.875875000 8.428044000 8.402460000

C -3.809755000 7.246202000 5.733776000

H -4.046096000 6.538339000 6.549358000

H -4.492550000 8.117938000 5.839532000

H -4.053739000 6.754619000 4.772974000

C -2.089366000 8.286809000 3.226369000

H -2.724780000 9.193591000 3.127247000

H -1.244018000 8.385422000 2.520340000

H -2.698672000 7.418148000 2.912544000

Br 1.806562000 5.975366000 6.551710000

Br -0.051356000 5.537080000 3.305597000

Br -1.666258000 4.457903000 6.563337000

[Ti(Me_4_-Cp)I_3_] – CH_2_Cl_2_


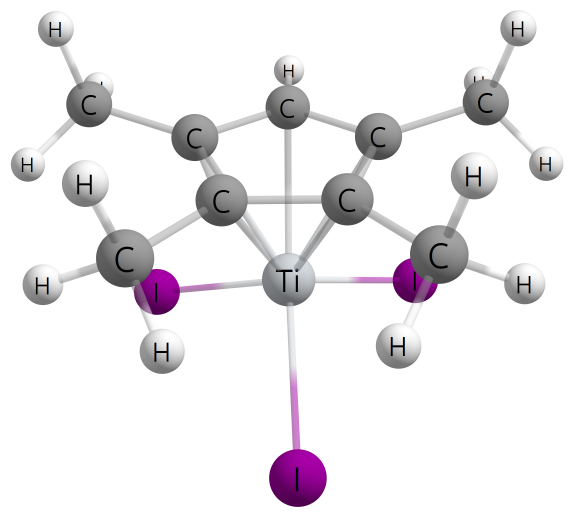


26

Ti -0.362055000 6.289087000 5.560515000

C -1.586624000 8.179643000 4.656637000

C -0.332992000 8.631209000 5.187636000

C -0.338390000 8.479983000 6.614321000

C -1.592202000 7.866739000 6.974261000

C -2.361345000 7.676039000 5.764314000

H 0.484170000 9.067739000 4.599234000

C -2.076326000 7.638314000 8.384215000

H -2.520759000 8.577506000 8.781914000

H -2.855007000 6.855540000 8.436096000

H -1.252336000 7.351676000 9.065037000

C 0.677342000 9.051870000 7.569798000

H 1.641033000 9.248541000 7.064525000

H 0.298570000 10.019653000 7.965958000

H 0.868188000 8.391724000 8.436983000

C -3.787137000 7.193040000 5.679245000

H -4.041813000 6.495474000 6.498578000

H -4.482224000 8.059056000 5.748919000

H -3.995198000 6.685870000 4.717727000

C -2.080265000 8.394545000 3.247905000

H -2.687777000 9.325692000 3.215540000

H -1.241613000 8.513839000 2.536391000

H -2.722400000 7.568488000 2.888720000

I 2.177513000 6.010482000 6.340065000

I -1.561228000 4.242340000 6.798029000

I -0.183040000 5.382173000 3.058835000

[Ti(Me_5_-Cp)Cl_3_] – CHCl_3_


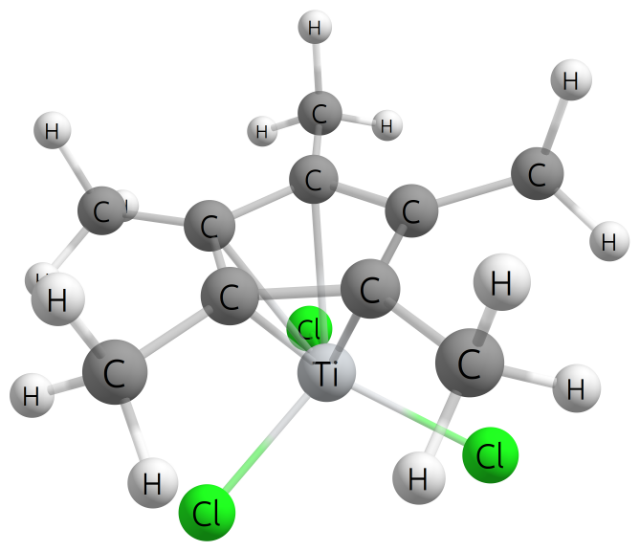


29

Ti -0.251485 -0.015123 0.709835

C 0.098472 1.201086 -1.349668

C -0.716920 0.047258 -1.641657

C 0.079551 -1.133489 -1.406163

C 1.374490 -0.710054 -0.938402

C 1.385113 0.732445 -0.900507

C 2.542486 -1.613767 -0.634800

H 3.151015 -1.773599 -1.551648

H 3.207782 -1.183075 0.135988

H 2.213549 -2.609636 -0.281561

C -0.321539 -2.551316 -1.722404

H -1.415119 -2.696349 -1.657345

H -0.008753 -2.807402 -2.758232

H 0.156157 -3.280070 -1.040916

C 2.566602 1.600112 -0.546462

H 3.207774 1.128882 0.221098

H 3.196118 1.778227 -1.445605

H 2.252080 2.589987 -0.165259

C -0.275694 2.640126 -1.598300

H 0.051809 2.941994 -2.617026

H -1.367004 2.802543 -1.536036

H 0.207643 3.325251 -0.876927

Cl -1.599804 -1.769190 1.222121

Cl -1.508391 1.770528 1.324670

Cl 1.379112 -0.106299 2.293305

C -2.132855 0.068001 -2.161476

H -2.141305 0.054923 -3.273276

H -2.707166 -0.812273 -1.815988

H -2.675744 0.975242 -1.836231

[Ti(Me_5_-Cp)Br_3_] – CHCl_3_


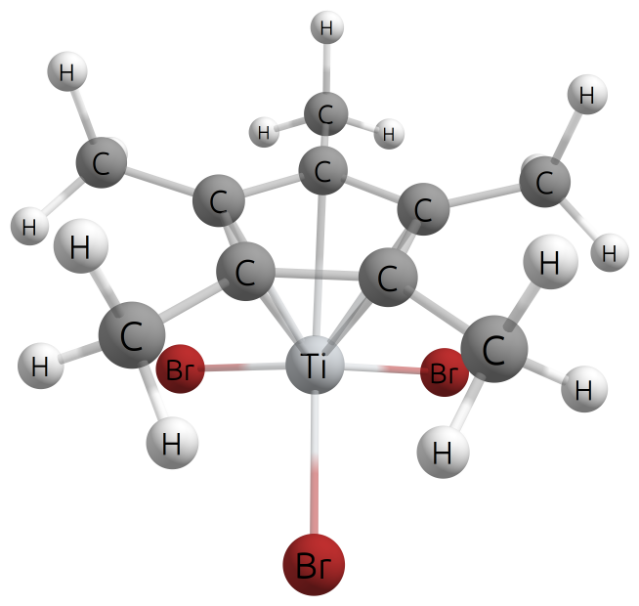


29

Ti -0.424684000 6.287964000 5.581949000

C -1.583767000 8.198168000 4.652781000

C -0.312452000 8.654681000 5.160260000

C -0.317699000 8.483922000 6.592825000

C -1.581368000 7.897684000 6.966978000

C -2.362525000 7.720320000 5.768039000

C -2.040157000 7.650244000 8.382220000

H -2.438886000 8.591558000 8.819674000

H -2.842333000 6.892049000 8.431668000

H -1.211824000 7.312960000 9.034177000

C 0.727426000 8.987738000 7.554405000

H 1.729156000 9.039733000 7.091305000

H 0.458909000 10.013060000 7.891311000

H 0.804522000 8.352928000 8.456748000

C -3.788628000 7.236800000 5.692075000

H -4.048777000 6.580693000 6.542544000

H -4.485477000 8.103220000 5.710189000

H -3.987179000 6.677592000 4.757939000

C -2.086951000 8.363820000 3.241881000

H -2.615424000 9.337876000 3.148759000

H -1.265175000 8.357989000 2.502877000

H -2.802862000 7.570475000 2.957438000

C 0.789665000 9.283193000 4.344201000

H 0.625322000 10.379175000 4.253445000

H 1.783065000 9.136533000 4.807753000

H 0.829112000 8.870388000 3.318438000

Br -1.593913000 4.480676000 6.723143000

Br 1.866732000 5.995542000 6.353108000

Br -0.294092000 5.460762000 3.293876000

[Ti(SiMe_3_-Cp)Cl_3_] – CH_2_Cl_2_


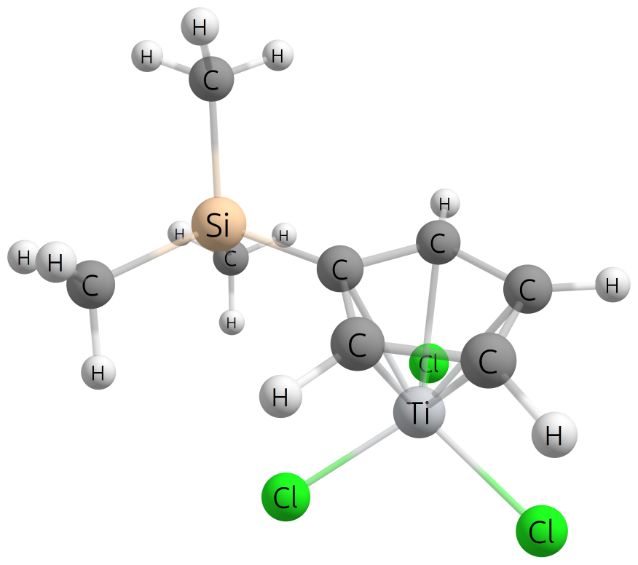


26

Ti -1.227637 -0.095039 -0.002049

C 0.230328 1.419203 1.151550

C -0.900526 2.170813 0.710333

C -0.890504 2.174902 -0.721435

C 0.240160 1.419802 -1.150063

C 0.957142 0.933253 0.005245

H -1.642299 2.639758 -1.368290

H 0.502963 1.215523 -2.193862

H 0.484438 1.222360 2.198807

H -1.664901 2.628133 1.349628

Si 2.612639 -0.039126 0.004814

C 2.455006 -1.647828 1.003379

H 2.117217 -1.456476 2.041801

H 1.730518 -2.341386 0.532445

H 3.440372 -2.156811 1.052464

C 3.911554 1.097276 0.809581

H 4.900379 0.592910 0.831025

H 4.024063 2.045831 0.245578

H 3.637846 1.350538 1.854418

C 3.080496 -0.410117 -1.797739

H 4.042631 -0.962112 -1.835689

H 2.310064 -1.034743 -2.293155

H 3.204020 0.521299 -2.387754

Cl -3.415663 0.282067 -0.402413

Cl -0.671839 -1.656692 -1.543739

Cl -1.274744 -1.247141 1.939397

[Ti(SiMe_3_-Cp)Br_3_] – CH_2_Cl_2_


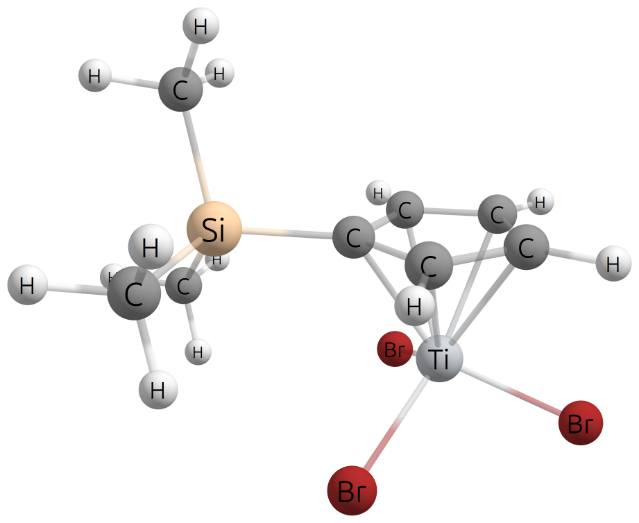


26

Ti 0.727314 0.015116 0.171569

C -0.814092 1.289039 1.505687

C 0.289554 0.926639 2.336775

C 0.284770 -0.498688 2.469999

C -0.811702 -1.002307 1.709108

C -1.518702 0.099763 1.099320

Br 0.178842 -1.693602 -1.462636

Br 3.033086 -0.524742 0.699240

Br 0.967462 2.044818 -1.132394

H 1.010539 -1.096272 3.030963

H -1.065364 -2.062243 1.595401

H -1.073380 2.313482 1.217429

H 1.028782 1.612567 2.767095

Si -3.192469 0.015713 0.162164

C -3.106867 0.971907 -1.475293

H -2.805196 2.027040 -1.316254

H -2.382921 0.510582 -2.175964

H -4.104790 0.974869 -1.961850

C -4.471867 0.824150 1.319689

H -5.479460 0.800482 0.854076

H -4.531565 0.288652 2.289453

H -4.219294 1.883797 1.529146

C -3.639680 -1.806408 -0.125286

H -4.631557 -1.878597 -0.618169

H -2.898507 -2.313261 -0.775068

H -3.700765 -2.364805 0.831571

[Ti(SiMe_3_-Cp)I_3_] – CH_2_Cl_2_


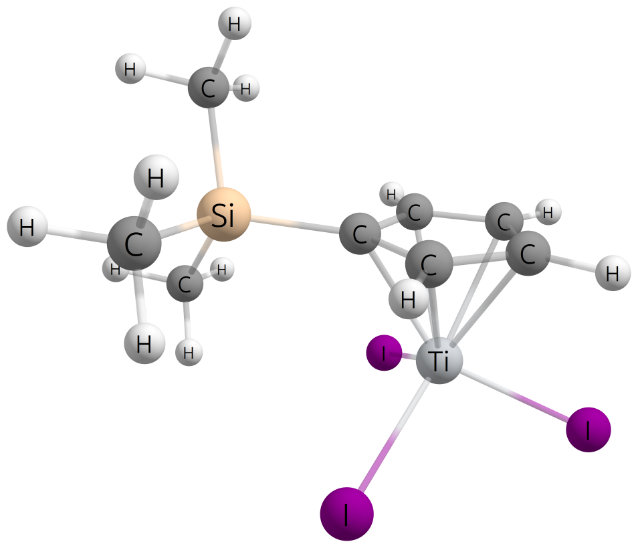


26

Ti 0.395176 -0.000600 0.337642

C -1.068833 1.233009 1.791156

C 0.061646 0.821881 2.559925

C 0.044300 -0.608437 2.630651

C -1.092080 -1.065750 1.898588

C -1.806284 0.069263 1.364152

H 0.778537 -1.238311 3.143889

H -1.376510 -2.115360 1.767701

H -1.336383 2.272359 1.573831

H 0.819025 1.480769 2.999821

Si -3.535320 0.044520 0.522495

C -3.505856 0.804463 -1.216571

H -3.109937 1.839791 -1.204833

H -2.881849 0.208409 -1.911471

H -4.538648 0.837734 -1.622864

C -4.673928 1.078710 1.646028

H -5.707507 1.082125 1.240719

H -4.711609 0.668433 2.675839

H -4.332887 2.132550 1.709182

C -4.132802 -1.757067 0.462288

H -5.148233 -1.802542 0.016191

H -3.465778 -2.393116 -0.154243

H -4.192209 -2.200356 1.477542

I 3.001621 -0.318167 0.804014

I -0.092218 -1.966504 -1.389736

I 0.431118 2.193369 -1.174121

[Ti(SnMe_3_-Cp)Cl_3_] – CH_2_Cl_2_


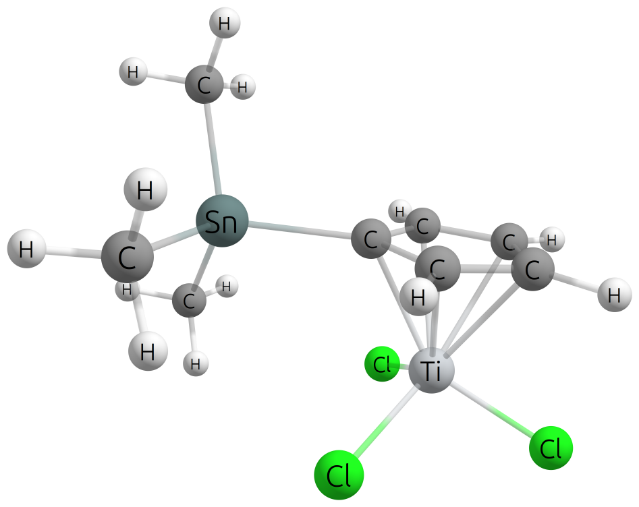


26

Ti -1.806475 -0.093366 0.005655

C -0.467431 1.523866 1.151359

C -1.642317 2.205805 0.707222

C -1.635155 2.200215 -0.723835

C -0.462614 1.506760 -1.150445

C 0.278754 1.069865 0.007486

H -2.414809 2.616090 -1.371657

H -0.193270 1.308969 -2.194034

H -0.203642 1.346671 2.199824

H -2.431908 2.621987 1.344348

C 1.975562 -1.932497 1.058514

H 1.576146 -1.754018 2.074326

H 1.269503 -2.573497 0.498932

H 2.960133 -2.433457 1.129669

C 3.641681 1.285610 1.034150

H 4.631626 0.790811 1.064211

H 3.729745 2.248246 0.495306

H 3.297971 1.474551 2.069127

C 2.728658 -0.309664 -2.113001

H 3.679044 -0.872628 -2.184598

H 1.925723 -0.882075 -2.614739

H 2.849103 0.671729 -2.610572

Cl -4.021052 0.108975 -0.394380

Cl -1.157814 -1.613734 -1.548478

Cl -1.751939 -1.239917 1.953066

Sn 2.207759 -0.022992 -0.000535

[Ti(SnMe_3_-Cp)Br_3_] – CH_2_Cl_2_


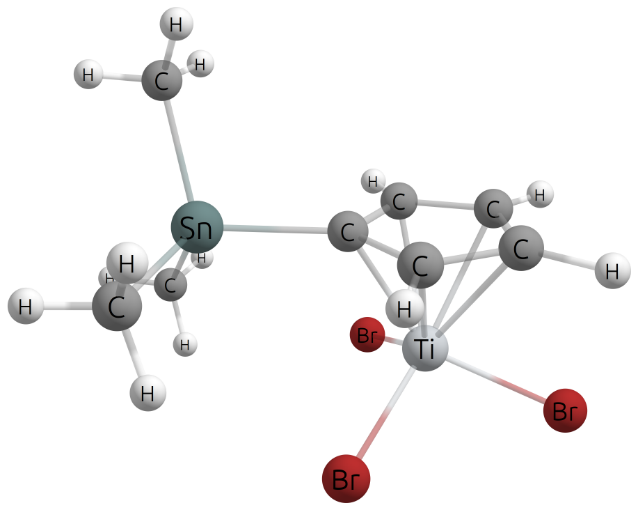


26

Ti 1.272773 -0.002708 0.190790

C -0.171664 1.193381 1.676011

C 0.955779 0.765449 2.442582

C 0.941068 -0.665915 2.477281

C -0.189205 -1.107768 1.725186

C -0.895311 0.039226 1.209982

H 1.684147 -1.305410 2.966171

H -0.458180 -2.155567 1.551327

H -0.426706 2.237251 1.463771

H 1.716022 1.412296 2.896054

C -2.636193 1.178466 -1.760167

H -2.310270 2.209738 -1.528311

H -1.897054 0.704261 -2.431905

H -3.624566 1.208639 -2.258191

C -4.280385 0.925842 1.449234

H -5.268271 0.955493 0.950214

H -4.357161 0.327494 2.377080

H -3.969140 1.957315 1.702448

C -3.279149 -2.096288 -0.300461

H -4.224507 -2.157786 -0.873188

H -2.463625 -2.556169 -0.889549

H -3.397522 -2.641952 0.655265

Sn -2.810743 0.011664 0.090540

Br 3.651197 -0.309006 0.588242

Br 0.762555 -1.769101 -1.399068

Br 1.268057 2.017720 -1.155557

[Ti((SiMe_3_)_2_-Cp)Cl_3_] – CH_2_Cl_2_


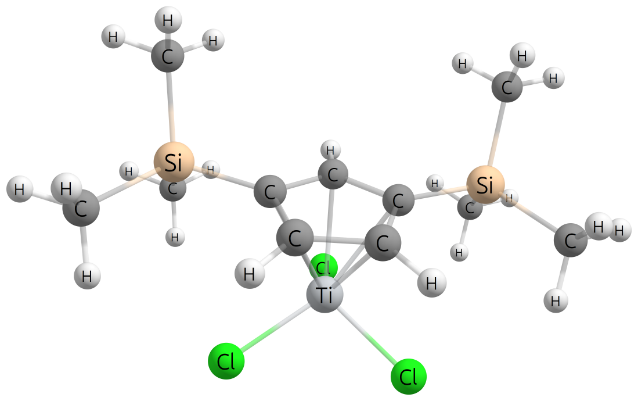


38

Ti -0.000445 -1.182042 -0.002412

C 0.003152 1.195576 0.270113

C -1.183194 0.866433 -0.477115

C -0.712568 0.317125 -1.725660

C 0.712783 0.315380 -1.726751

C 1.186936 0.865287 -0.479563

H -1.346856 -0.068808 -2.531814

H 1.345413 -0.075219 -2.532300

H 0.003787 1.621416 1.280508

C 3.386455 0.506361 1.731178

H 2.692958 0.898296 2.502766

H 3.303726 -0.598394 1.728667

H 4.419730 0.776349 2.034211

C 3.180447 3.124513 0.057967

H 4.218994 3.407798 0.329864

H 2.953310 3.572159 -0.931358

H 2.498110 3.580137 0.804643

C 4.154507 0.497550 -1.308247

H 5.214259 0.696515 -1.045056

H 4.023351 -0.599390 -1.395954

H 3.965321 0.950073 -2.303581

Si 3.005246 1.227717 0.015034

Cl -1.698751 -2.539203 -0.650711

Cl 1.823975 -2.445804 -0.467274

Cl -0.126504 -1.263597 2.254002

Si -3.003108 1.231270 0.010343

C -3.183306 3.128013 0.028105

H -4.221722 3.412359 0.299326

H -2.500090 3.593448 0.767966

H -2.959066 3.564334 -0.966866

C -4.146038 0.474879 -1.303584

H -4.007691 -0.622558 -1.375247

H -5.207051 0.670191 -1.042737

H -3.960733 0.913254 -2.305862

C -3.395895 0.532679 1.732606

H -3.281626 -0.568975 1.756370

H -2.729715 0.959222 2.509692

H -4.442472 0.780389 2.007968

[Ti((SiMe_3_)_3_-Cp)Cl_3_] – CH_2_Cl_2_


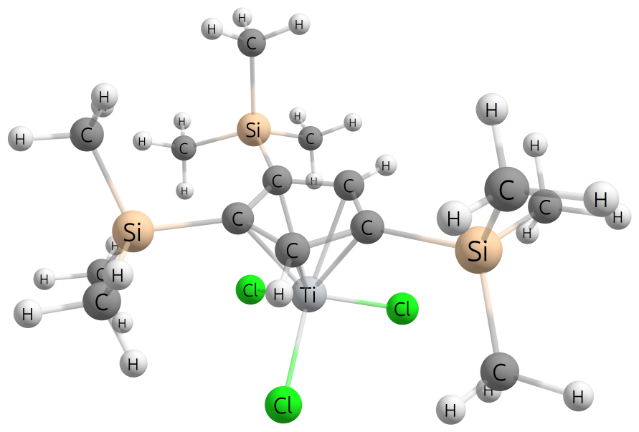


50

Ti -0.452910 0.105682 -1.268562

C -0.589548 -1.211496 0.720926

C -1.467238 -0.090251 0.921020

C -0.606841 1.057247 0.919405

C 0.770456 0.674133 0.727073

C 0.782269 -0.782998 0.607095

H -0.952318 2.090904 1.028557

H -0.926447 -2.251953 0.640668

C 1.532371 -3.629663 -0.365960

H 0.642560 -4.081623 0.117558

H 1.262947 -3.367044 -1.408455

H 2.321337 -4.409608 -0.402246

C 2.441334 -2.591449 2.417288

H 3.195975 -3.402283 2.495113

H 2.804276 -1.731734 3.016689

H 1.500956 -2.955671 2.879344

C 3.836315 -1.570893 -0.157424

H 4.530300 -2.436794 -0.114447

H 3.731147 -1.270130 -1.217217

H 4.312698 -0.740409 0.398926

Si 2.176454 -2.113838 0.589292

Cl -1.045296 2.172772 -2.001264

Cl 1.316798 -0.363683 -2.604137

Cl -2.034213 -1.247669 -2.175520

Si 2.168463 1.999565 0.828462

Si -3.352693 -0.110327 1.273562

C -3.564296 0.505705 3.064928

H -3.180752 1.539457 3.187767

H -4.638536 0.507541 3.345821

H -3.025665 -0.146380 3.782911

C -4.272300 1.051964 0.084004

H -3.852125 2.077934 0.107372

H -4.212275 0.683887 -0.959143

H -5.343438 1.115485 0.368721

C -3.991291 -1.891361 1.117077

H -3.838090 -2.285236 0.092547

H -3.480865 -2.571064 1.830254

H -5.078237 -1.926229 1.338866

C 3.247960 1.593646 2.345946

H 3.784720 0.629973 2.242054

H 4.009027 2.389128 2.488986

H 2.631905 1.542194 3.267068

C 1.316882 3.678593 1.112629

H 0.733695 3.701110 2.055743

H 2.086855 4.475586 1.176164

H 0.635261 3.936376 0.276556

C 3.232656 2.140379 -0.738695

H 2.618064 2.437824 -1.611315

H 4.002200 2.924653 -0.577412

H 3.751422 1.198034 -0.993712

[Ti(Cp)_2_F_2_] – CH_2_Cl_2_


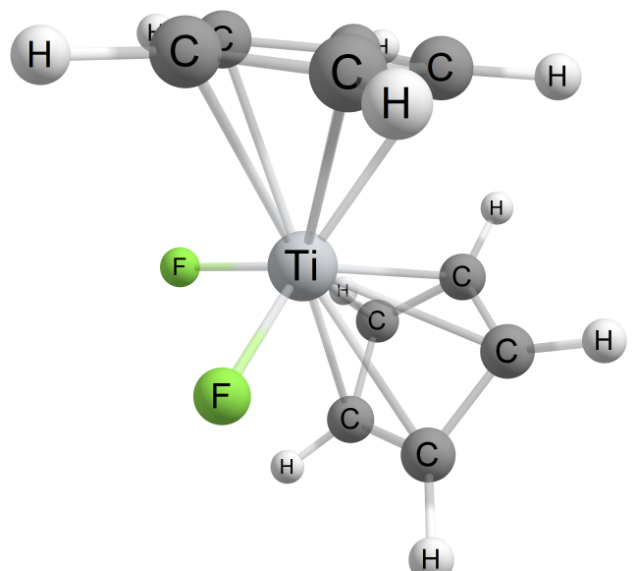


23

H 1.075826 -2.447835 0.901561

H -1.075781 -2.447941 -0.901292

H -1.304570 -1.871491 1.757507

H 1.304488 -1.871694 -1.757319

C 1.484351 -1.526208 0.474708

C -1.484325 -1.526264 -0.474567

C -1.608504 -1.225436 0.926605

C 1.608458 -1.225541 -0.926507

C 2.008573 -0.424991 1.207024

C -2.008504 -0.425129 -1.207040

H 2.015314 -0.321385 2.297887

H -2.015190 -0.321649 -2.297915

C -2.236983 0.049355 1.042946

C 2.236923 0.049237 -1.043029

Ti 0.000001 0.355142 -0.000002

C 2.464098 0.555241 0.267905

C -2.464090 0.555206 -0.268055

H -2.430691 0.584208 1.978875

H 2.430584 0.583981 -1.979030

H 2.870552 1.542127 0.516157

H -2.870532 1.542063 -0.516442

F -0.018290 1.560210 -1.369808

F 0.018291 1.560196 1.369819

[Ti(OCHMe_2_)_3_Cl] – CHCl3


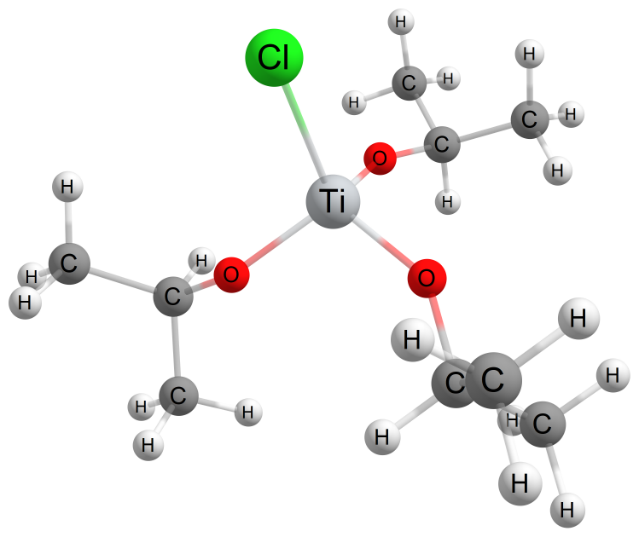


35

Ti 0.036910 -0.053024 0.454493

O -0.348168 -1.651290 -0.252931

O -1.192902 1.174641 0.000367

O 1.645650 0.480185 -0.127631

C -1.462812 2.492595 -0.500457

H -0.487775 2.950532 -0.787867

C -1.154508 -2.603044 -0.960295

H -1.019166 -2.397258 -2.047492

C 2.884894 0.128858 -0.760940

H 2.789593 -0.919012 -1.129420

C -0.632834 -4.017775 -0.656214

H -0.747089 -4.250032 0.422286

H -1.196914 -4.773588 -1.239378

H 0.439669 -4.104109 -0.919875

C -2.637539 -2.414380 -0.600298

H -2.805431 -2.615716 0.477597

H -2.962388 -1.378036 -0.817003

H -3.271328 -3.108815 -1.188064

C -2.350653 2.387887 -1.752036

H -3.329526 1.931248 -1.499758

H -2.535023 3.393079 -2.182532

H -1.862479 1.763887 -2.526739

C -2.096870 3.331547 0.621946

H -3.068614 2.895389 0.931354

H -1.432387 3.363442 1.507659

H -2.272480 4.371076 0.278337

C 4.013449 0.190862 0.282790

H 4.976153 -0.127849 -0.165636

H 4.130906 1.224420 0.667961

H 3.788261 -0.475350 1.138670

C 3.119979 1.059931 -1.961922

H 3.206487 2.113684 -1.626497

H 4.054916 0.783161 -2.489976

H 2.280961 0.989808 -2.682210

Cl 0.092727 -0.225330 2.743873

[Ti(OCHMe_2_)_2_Cl_2_] – CHCl_3_


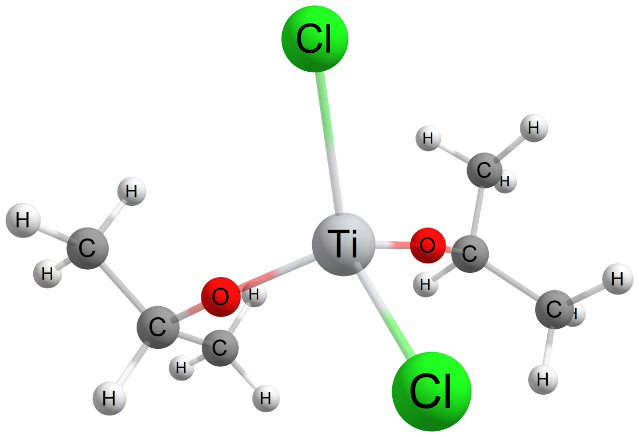


25

Ti -0.077711 -0.722557 -0.032715

O -1.601337 0.098565 -0.373029

O 1.237405 0.439305 0.072652

C 2.193856 1.511997 0.069831

H 1.638149 2.430592 -0.224484

C -2.591156 1.123452 -0.559679

H -3.192802 0.804027 -1.440421

C -3.499655 1.172515 0.678959

H -2.922328 1.479714 1.574524

H -4.319918 1.901307 0.522300

H -3.948048 0.179158 0.874752

C -1.908732 2.463159 -0.877191

H -1.298147 2.806654 -0.017401

H -1.250674 2.367696 -1.763134

H -2.671071 3.238031 -1.094558

C 2.748162 1.688643 1.491894

H 3.293701 0.778907 1.813675

H 3.447706 2.547951 1.523220

H 1.928615 1.879339 2.212066

C 3.274477 1.210750 -0.980695

H 3.836938 0.294435 -0.710058

H 2.819992 1.060554 -1.979397

H 3.989356 2.055431 -1.044950

Cl -0.239295 -1.792187 1.957842

Cl 0.355055 -2.163907 -1.726201

[Ti(OCHMe_2_)Cl_3_] – CHCl_3_


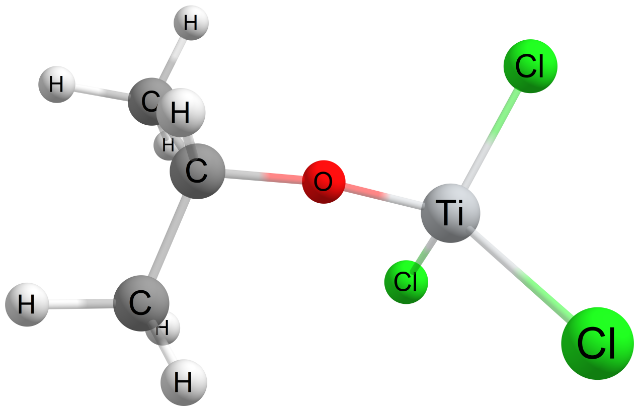


15

Ti -0.646452 0.002394 -0.008520

O 1.039420 0.107450 -0.410809

C 2.470669 0.031707 -0.608929

H 2.609592 -0.062847 -1.708090

C 3.105808 1.340015 -0.118925

H 2.957228 1.462736 0.972589

H 4.194346 1.327772 -0.326576

H 2.662085 2.211132 -0.638687

C 3.001714 -1.227592 0.090058

H 2.846006 -1.161522 1.185507

H 2.487079 -2.132104 -0.288262

H 4.087104 -1.336180 -0.105512

Cl -0.817733 -0.035066 2.216909

Cl -1.710950 1.792327 -0.818041

Cl -1.436376 -1.879969 -0.915944

[Ti(OCMe_3_)] – CHCl_3_


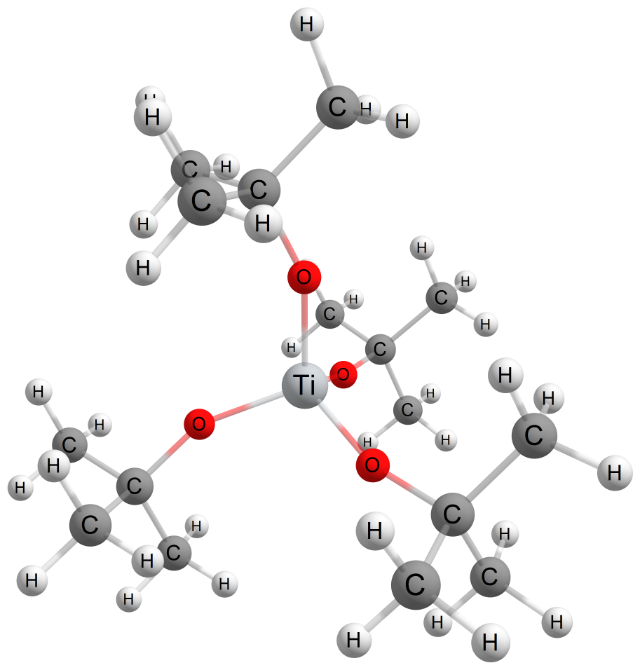


57

Ti -0.018509 -0.013658 -0.083573

O -1.512379 0.018060 -1.115178

O -0.191954 -1.283196 1.202310

O 1.408605 -0.400740 -1.136107

O 0.220513 1.599571 0.698560

C -2.878680 -0.111727 -1.521408

C 2.583854 -0.104132 -1.904689

C 0.167698 -2.500894 1.874019

C 0.134487 2.721586 1.585476

C -3.261643 1.166880 -2.306225

H -2.602491 1.289692 -3.188617

H -4.312032 1.119880 -2.658906

H -3.149126 2.062588 -1.663107

C -2.989299 -1.362316 -2.427720

H -2.327869 -1.259032 -3.311042

H -2.683244 -2.270051 -1.870282

H -4.029301 -1.505860 -2.784994

C -0.934062 -2.798489 2.919572

H -1.917624 -2.918607 2.423178

H -0.710394 -3.729616 3.478848

H -1.012877 -1.965708 3.646583

C 0.251192 -3.635990 0.825137

H -0.725887 -3.766149 0.318225

H 1.011134 -3.394276 0.056023

H 0.526969 -4.599029 1.300733

C 3.747683 0.188506 -0.927308

H 3.935774 -0.688495 -0.275880

H 4.682736 0.418830 -1.477277

H 3.500587 1.053819 -0.280869

C 2.896518 -1.347265 -2.772609

H 3.077076 -2.232357 -2.130520

H 2.044259 -1.574332 -3.443683

H 3.797968 -1.180598 -3.396553

C -0.434214 3.918669 0.786094

H -0.519325 4.821170 1.424869

H 0.225440 4.159077 -0.071411

H -1.440319 3.674859 0.390445

C 1.561791 3.032244 2.098429

H 2.235298 3.269083 1.250766

H 1.556097 3.898966 2.790157

H 1.976988 2.157776 2.638170

C -0.801212 2.354200 2.762285

H -1.816285 2.111649 2.388996

H -0.412102 1.471025 3.306308

H -0.886872 3.195807 3.479110

C -3.766164 -0.268692 -0.262857

H -3.459307 -1.161016 0.318106

H -3.669679 0.620347 0.392297

H -4.834452 -0.381846 -0.538332

C 1.536579 -2.291480 2.565574

H 2.313216 -2.039506 1.816282

H 1.478142 -1.461080 3.297109

H 1.854478 -3.207159 3.104110

C 2.293587 1.126934 -2.797403

H 1.441565 0.920723 -3.475585

H 2.035271 2.005789 -2.173336

H 3.176414 1.386360 -3.416209

[Ti(OCH(Me)Et)_4_] – CHCl_3_


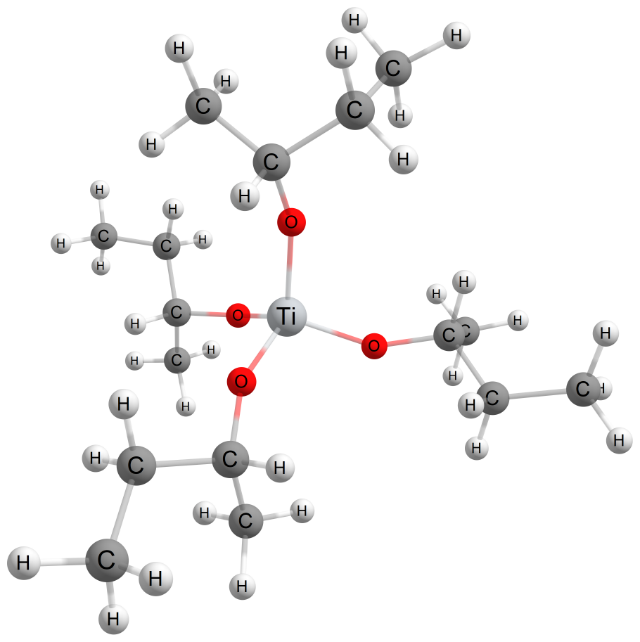


57

Ti 0.031016 -0.066262 -0.243227

O 1.465157 -0.391289 -1.328329

O 0.249275 1.527455 0.579602

O -1.461891 -0.074600 -1.284453

O -0.112358 -1.326657 1.058332

C 2.887483 -0.330881 -1.421223

H 3.272626 0.324206 -0.600933

C -2.803802 -0.534733 -1.445129

H -3.010850 -1.300566 -0.658436

C -0.003919 2.907801 0.839997

H -1.008278 2.983400 1.322342

C -0.081119 -1.614640 2.456806

H -0.052202 -0.644126 3.007510

C 3.470882 -1.750788 -1.215202

H 3.104872 -2.400077 -2.040473

H 3.026157 -2.154823 -0.281379

C 3.267292 0.308772 -2.769703

H 2.912004 -0.321803 -3.610919

H 2.797974 1.308039 -2.864457

H 4.364613 0.434703 -2.864181

C 1.052801 3.422595 1.847819

H 2.052607 3.364598 1.364460

H 1.074038 2.705620 2.695551

C -0.037728 3.689314 -0.485932

H 0.952427 3.650187 -0.985331

H -0.790634 3.250906 -1.170117

H -0.303823 4.752226 -0.319461

C -3.775230 0.651816 -1.227097

H -3.577337 1.413279 -2.012997

H -3.503747 1.124099 -0.259146

C -2.935950 -1.203360 -2.825791

H -2.745248 -0.466378 -3.633079

H -2.199187 -2.024791 -2.925398

H -3.948055 -1.630955 -2.972104

C 1.204366 -2.392825 2.797257

H 1.250558 -2.619818 3.882205

H 1.256261 -3.347459 2.237100

H 2.097146 -1.792201 2.532602

C -1.389324 -2.336943 2.873327

H -1.371653 -2.460808 3.978281

H -2.232122 -1.647407 2.652706

C 5.007558 -1.815363 -1.124474

H 5.346664 -2.847470 -0.902220

H 5.496802 -1.505358 -2.070246

H 5.394742 -1.158216 -0.317126

C 0.797024 4.844962 2.381471

H 1.547501 5.117076 3.150821

H 0.854985 5.609877 1.580706

H -0.205511 4.927886 2.851849

C -5.268574 0.272838 -1.213447

H -5.896671 1.155315 -0.976422

H -5.609982 -0.120142 -2.192540

H -5.483683 -0.502730 -0.448280

C -1.644788 -3.694231 2.191324

H -0.880192 -4.449526 2.464875

H -2.632337 -4.103380 2.486163

H -1.636115 -3.590887 1.087837

[Ti(OCH_2_CMe_3_)_4_] – CHCl_3_


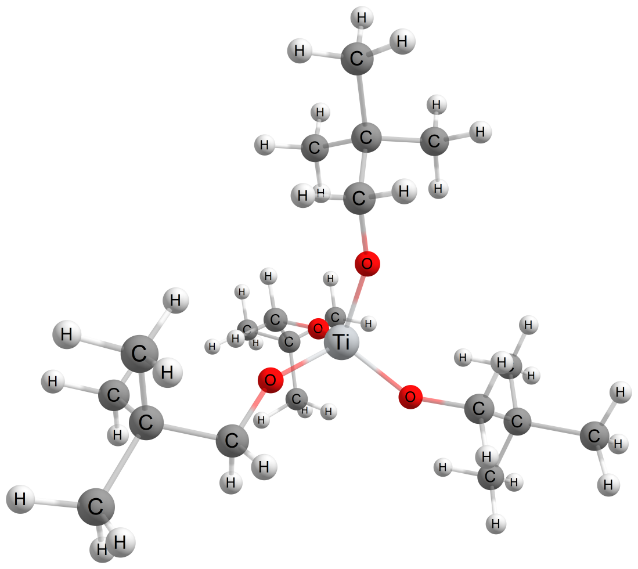


69

Ti 0.023502 0.046371 -0.332029

O -0.262699 -0.866979 1.224652

O 1.610637 -0.465797 -1.033513

O -1.338895 -0.323555 -1.484092

O 0.055325 1.833838 -0.033480

C 0.332545 -1.553347 2.308465

H 1.308287 -1.991024 1.988589

C -2.262330 0.290100 -2.367702

H -2.253507 1.395003 -2.213663

C 2.411840 -1.048401 -2.045399

H 2.171611 -0.573241 -3.024954

C 0.689034 3.083176 0.155467

H 1.795812 2.950744 0.109332

C -0.572529 -2.690800 2.874741

C 3.939942 -0.905023 -1.771533

C -3.717916 -0.239288 -2.187431

C 0.312312 3.771120 1.503634

H 0.405155 3.757737 -0.685989

H -1.936585 0.100546 -3.417527

H 0.551596 -0.828319 3.128237

H 2.158389 -2.130566 -2.133305

C 4.322902 0.595184 -1.702098

H 4.091680 1.113123 -2.656450

H 3.767565 1.110541 -0.893491

H 5.407728 0.718611 -1.505006

C 4.304167 -1.607683 -0.439038

H 3.752034 -1.160006 0.410749

H 4.055054 -2.688964 -0.474125

H 5.389642 -1.517789 -0.227695

C 4.689089 -1.585411 -2.946585

H 5.786970 -1.512875 -2.805847

H 4.431125 -2.662277 -3.023961

H 4.442976 -1.106830 -3.917450

C 0.747802 2.874609 2.690973

H 0.248932 1.886399 2.648683

H 1.844833 2.703148 2.682709

H 0.488334 3.348047 3.660275

C 1.064702 5.125619 1.563721

H 0.777428 5.788432 0.720785

H 0.831617 5.662192 2.506216

H 2.164765 4.982351 1.522892

C -1.217288 4.015443 1.556803

H -1.508573 4.499644 2.511668

H -1.546631 4.677057 0.728299

H -1.774768 3.062070 1.471406

C -4.216940 0.076843 -0.754243

H -3.568832 -0.398230 0.008578

H -4.220130 1.170558 -0.563317

H -5.251585 -0.294802 -0.603947

C -4.611801 0.485287 -3.226725

H -4.277542 0.278799 -4.265015

H -5.665509 0.149149 -3.140739

H -4.597711 1.585363 -3.078281

C -3.752371 -1.768777 -2.436055

H -4.779843 -2.168108 -2.309420

H -3.418329 -2.015443 -3.465792

H -3.086643 -2.299989 -1.727581

C -1.911726 -2.091504 3.374533

H -2.445998 -1.576522 2.552134

H -2.576810 -2.884438 3.774948

H -1.742420 -1.351174 4.184409

C 0.184045 -3.348973 4.057076

H 0.393207 -2.616211 4.864391

H -0.415806 -4.171299 4.498328

H 1.153815 -3.779361 3.730297

C -0.843227 -3.745011 1.771474

H -1.349149 -3.286245 0.899262

H 0.102770 -4.203405 1.413829

H -1.490462 -4.561587 2.153156

[Ti(OCHMe_2_)_3_(OCH_2_CH_2_NMe_2_)] – CHCl_3_


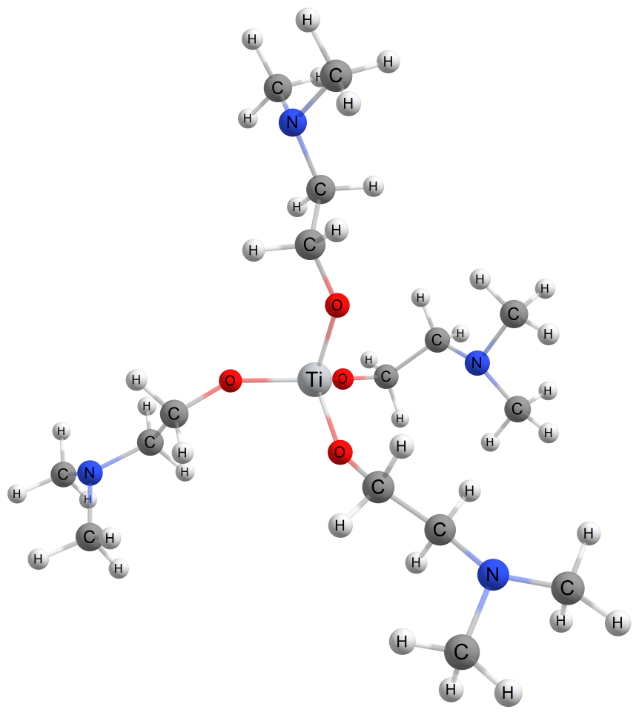


65

Ti 0.344931 0.091483 0.316763

O -0.067116 0.173464 2.089335

O 2.155694 0.247559 0.185003

O -0.133938 -1.473716 -0.462049

O -0.391446 1.468838 -0.623707

C -1.014261 0.197397 3.131991

H -0.578412 0.734918 4.007137

C -0.894932 -2.160781 -1.436421

H -1.434138 -1.441410 -2.094376

C 3.280224 -0.373529 -0.413014

H 3.826061 0.364468 -1.043398

C -0.065914 2.591282 -1.424429

H 0.998084 2.551721 -1.751813

C -2.324286 0.909933 2.741745

H -2.913820 1.105507 3.678067

H -2.050955 1.901102 2.327119

C 4.242837 -0.898481 0.676786

H 3.691932 -1.634655 1.322248

H 4.498520 -0.038489 1.328455

C -1.924589 -3.092362 -0.759263

H -1.381608 -3.781124 -0.058017

H -2.575612 -2.451770 -0.132152

C -0.297033 3.906039 -0.644313

H -1.354751 3.924970 -0.266954

H 0.356118 3.877578 0.251466

H -0.695899 2.565707 -2.341919

H 2.947455 -1.200250 -1.081338

H -0.201279 -2.744136 -2.082923

H -1.224747 -0.843431 3.473114

N 5.487244 -1.452242 0.127510

N -2.761518 -3.815706 -1.725521

N -3.118841 0.187757 1.735846

N 0.040700 5.100139 -1.429647

C -0.985601 5.453858 -2.413664

H -0.631313 6.292393 -3.047072

H -1.202517 4.599149 -3.083350

H -1.956159 5.769784 -1.942896

C 0.399502 6.247712 -0.593669

H 0.732477 7.091282 -1.232620

H -0.449224 6.623190 0.040104

H 1.235058 5.982859 0.084823

C -3.946914 1.079665 0.919143

H -3.307027 1.824395 0.406786

H -4.476735 0.493092 0.141310

H -4.724913 1.632623 1.510939

C -3.909191 -0.899488 2.319869

H -4.705760 -0.531151 3.021287

H -4.407139 -1.482670 1.519328

H -3.259936 -1.595559 2.885740

C -2.071964 -4.944908 -2.355400

H -1.812924 -5.762768 -1.629297

H -2.712032 -5.387660 -3.145460

H -1.130432 -4.614909 -2.835721

C -4.051098 -4.223197 -1.164202

H -4.679408 -4.683097 -1.954457

H -3.960842 -4.971725 -0.330840

H -4.593187 -3.341494 -0.767342

C 6.584283 -1.444276 1.097572

H 6.403662 -2.111749 1.983420

H 7.521234 -1.786202 0.611759

H 6.752207 -0.417097 1.479123

C 5.314524 -2.777325 -0.473876

H 6.259983 -3.101686 -0.954303

H 5.024952 -3.565499 0.273335

H 4.532362 -2.756873 -1.257200

[Ti(NEt_2_)_4_] – C_6_H_6_


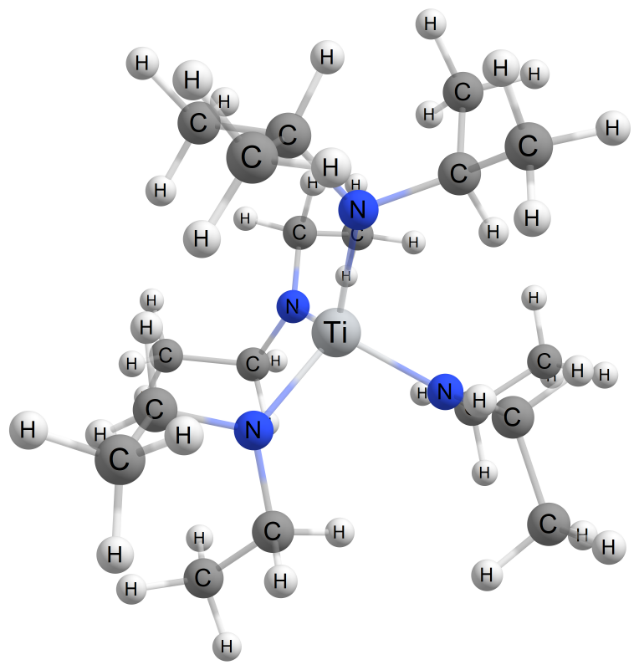


67

C -1.554737 -2.625042 -0.179747

C 1.536556 1.190415 -2.388733

N 1.925227 -0.555885 0.537674

N 0.271647 0.650382 -1.854026

H 1.333430 2.104778 -2.995454

H 2.165667 1.531993 -1.542530

N -1.422292 -1.173865 0.137239

Ti 0.134220 0.056083 0.001823

C -0.809814 0.584851 -2.854234

H -1.576733 -0.122367 -2.486799

C -2.684467 -0.548501 0.644536

H -2.430672 0.508182 0.857756

H 0.103724 3.098867 -0.507559

N -0.161811 1.693668 1.011300

H -0.347172 0.370190 2.632104

C -0.461958 1.460628 2.436613

C -0.224263 3.086244 0.549667

H 0.509179 3.717636 1.107729

C 2.410107 -1.901795 0.196483

C 2.909875 0.284893 1.247889

H 3.273793 -1.847946 -0.510024

H 1.612109 -2.426979 -0.367765

H 3.122673 -0.128251 2.264783

H 2.441426 1.272449 1.411501

H -1.534418 1.683224 2.662153

H -2.639771 -2.865924 -0.200749

H -0.428217 0.152784 -3.810635

C -1.493522 1.937462 -3.164946

H -0.774182 2.693478 -3.540756

H -2.272280 1.805643 -3.945553

H -1.981123 2.354497 -2.260944

C 2.355250 0.197323 -3.246038

H 1.782526 -0.160988 -4.125536

H 3.279850 0.680316 -3.626726

H 2.649413 -0.686771 -2.646868

C 2.824376 -2.783827 1.398707

H 3.071041 -3.810406 1.057083

H 3.718597 -2.384971 1.918104

H 2.005947 -2.853833 2.143723

C 4.262715 0.496909 0.526856

H 4.887875 1.208193 1.105510

H 4.844990 -0.440988 0.424840

H 4.120148 0.916855 -0.489338

C -1.025347 -2.980274 -1.591409

H -1.571877 -2.420878 -2.374849

H 0.052411 -2.745797 -1.706194

H -1.151729 -4.064262 -1.793483

C -0.936110 -3.552975 0.898668

H -1.334898 -3.321348 1.904379

H -1.165613 -4.617086 0.677683

H 0.163996 -3.450786 0.942831

C -3.204925 -1.157330 1.974512

H -4.058139 -0.561879 2.361403

H -3.564680 -2.198605 1.845003

H -2.413269 -1.162271 2.749287

C -3.822220 -0.504341 -0.412347

H -4.174243 -1.517691 -0.695060

H -4.699324 0.044634 -0.010906

H -3.491386 0.011861 -1.334189

C 0.410591 2.229308 3.456473

H 0.269413 3.326205 3.380900

H 0.130689 1.932581 4.488365

H 1.488451 2.013978 3.319648

C -1.615157 3.756049 0.648249

H -1.572899 4.788646 0.243331

H -2.372799 3.191629 0.066621

H -1.975844 3.827504 1.694570

[Ti(CH_2_C(Me_2_)(C_6_H_5_)]_4_ – C_6_H_6_


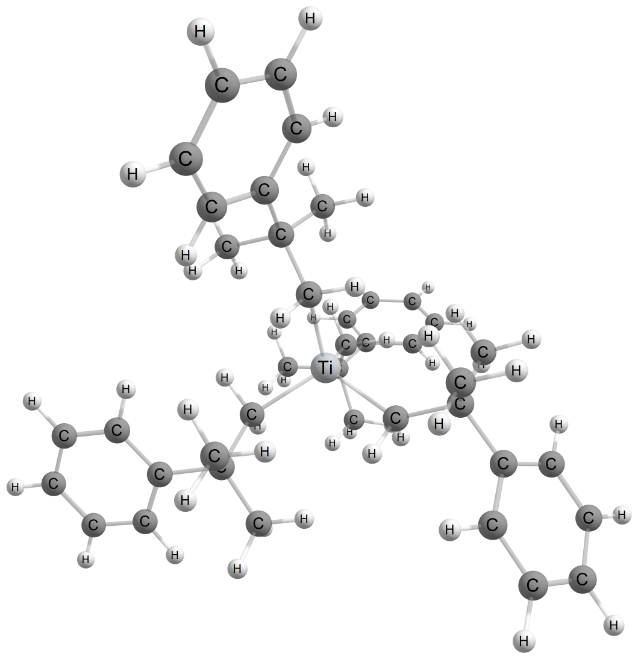


93

Ti 0.027502 0.028904 0.002560

C -1.269751 -0.136662 -1.651764

H -2.006568 0.610038 -1.265625

H -0.666460 0.409742 -2.415761

C -1.210101 0.208259 1.699939

H -1.964326 -0.537352 1.346368

H -0.578616 -0.339340 2.440681

C 1.263476 -1.674808 0.162278

H 2.013358 -1.349952 -0.601459

H 0.600862 -2.405072 -0.360352

C 1.314632 1.685611 -0.196776

H 0.753527 2.429550 0.418946

H 2.106391 1.279644 0.479104

C -1.918210 1.417492 2.392790

C -2.007154 -1.341821 -2.319414

C 1.994844 -2.354798 1.365336

C 1.956933 2.391085 -1.435028

C 2.965355 -3.441156 0.806725

C 2.450571 -4.556781 0.095997

C 4.371000 -3.367419 0.959450

C 3.293778 -5.549142 -0.432911

H 1.365681 -4.655031 -0.054418

C 5.222441 -4.358529 0.428090

H 4.827055 -2.528247 1.499567

C 4.690467 -5.455758 -0.270483

H 2.857005 -6.400499 -0.976989

H 6.310670 -4.266798 0.566080

H 5.353614 -6.229783 -0.684700

C 3.050622 3.383158 -0.930717

C 2.931644 4.788657 -1.053587

C 4.222503 2.887291 -0.301761

C 3.932181 5.657323 -0.570389

H 2.048940 5.230495 -1.532257

C 5.224862 3.747944 0.178491

H 4.360294 1.802956 -0.179614

C 5.085328 5.143861 0.047426

H 3.804181 6.744776 -0.683353

H 6.120870 3.325089 0.658010

H 5.867004 5.820834 0.423112

C -3.066006 -0.786871 -3.322523

C -4.175136 -0.041475 -2.843483

C -2.974959 -0.977573 -4.722121

C -5.144186 0.485732 -3.714531

H -4.288106 0.138112 -1.764362

C -3.942036 -0.447851 -5.601699

H -2.140462 -1.546230 -5.151109

C -5.032868 0.285785 -5.104928

H -5.990931 1.057073 -3.304515

H -3.836884 -0.615595 -6.684502

H -5.788370 0.697799 -5.790412

C -2.930671 0.865174 3.443900

C -2.769401 1.051208 4.838278

C -4.064451 0.124217 3.018748

C -3.692871 0.521854 5.763556

H -1.912444 1.615196 5.227116

C -4.990139 -0.402850 3.936030

H -4.232225 -0.052415 1.946085

C -4.809294 -0.207197 5.319612

H -3.533188 0.685837 6.840251

H -5.857962 -0.970680 3.567319

H -5.530791 -0.619343 6.040717

C -0.850299 2.325249 3.056698

H -0.222487 1.769773 3.781459

H -0.172996 2.744978 2.288558

H -1.315019 3.178836 3.589642

C -2.688721 2.269780 1.339159

H -1.985026 2.701540 0.599007

H -3.433599 1.671140 0.779671

H -3.230336 3.106875 1.825275

C -2.729486 -2.200418 -1.237993

H -1.992324 -2.638194 -0.534697

H -3.446468 -1.604675 -0.640219

H -3.293487 -3.033717 -1.704632

C -0.967394 -2.244206 -3.033709

H -0.381069 -1.685648 -3.790144

H -0.248619 -2.656146 -2.300030

H -1.451887 -3.102375 -3.541223

C 0.964852 -3.040362 2.311946

H 0.266708 -2.295684 2.742573

H 0.356528 -3.800887 1.784646

H 1.481061 -3.547304 3.152320

C 2.754263 -1.274465 2.180049

H 3.480240 -0.715409 1.557085

H 2.036138 -0.539546 2.593109

H 3.307203 -1.715711 3.033720

C 2.618773 1.339375 -2.376793

H 3.380303 0.728911 -1.853756

H 1.856001 0.643968 -2.782763

H 3.118219 1.834892 -3.234340

C 0.849454 3.125312 -2.235222

H 0.105563 2.399692 -2.615446

H 0.302700 3.860036 -1.611466

H 1.266404 3.662613 -3.110588
